# Supplementary material for: Novel Flavonoid Glycosides of Quercetin from Leaves and Flowers of Gaiadendron punctatum G.Don. (Violeta de Campo), used by the Saraguro Community in Southern Ecuador, Inhibit α-Glucosidase Enzyme
Source: Molecules. 2019 Nov 22;24(23):4267. doi: 10.3390/molecules24234267 (PMC6930599; doi:10.3390/molecules24234267)
Supplement: Supplementary file 1 [file molecules-24-04267-s001.pdf]

Supplementary Materials

Novel Flavonoid Glycosides of quercetin from leaves and flowers of Gaiadendron punctatum G.Don. (violeta de campo), used by the Saraguro community in Southern Ecuador.

Héctor Cedeño[1], Sandra Espinosa[1], José Miguel Andrade[1], Luis Cartuche[1] and Omar Malagón[1].

[1]Departamento de Química, Universidad Técnica Particular de Loja, Loja 1101608, Ecuador

Figure S1. ^{1}H NMR spectrum of compound LR 29 – 13 or nicotiflorin in methanol – d_4.
Figure S2. ^{13}C NMR spectrum of compound LR 29 – 13 or nicotiflorin in methanol – d_4.
Figure S3. DEPT NMR spectrum of compound LR 29 – 13 or nicotiflorin in methanol – d_4.
Figure S4. COSY NMR spectrum of compound LR 29 – 13 or nicotiflorin in methanol – d_4.
Figure S5. HSQC NMR spectrum of compound LR 29 – 13 or nicotiflorin in methanol – d_4.
Figure S6. HMBC spectrum of compound LR 29 – 13 or nicotiflorin in methanol – d_4.
Figure S7. ^{1}H NMR spectrum of compound LR 27 – 98 or rutin in methanol – d_4.
Figure S8. ^{13}C NMR spectrum of compound LR 27 – 98 or rutin in methanol – d_4.
Figure S9. ^{1}H NMR spectrum of compound LR 28 – 90 or rutin in methanol – d_4.
Figure S10. ^{13}C NMR spectrum of compound LR 28 – 90 or rutin in methanol – d_4.
Figure S11. DEPT NMR spectrum of compound LR 28 – 90 or rutin in methanol – d_4.
Figure S12. COSY NMR spectrum of compound LR 28 – 90 or rutin in methanol – d_4.
Figure S13. HSQC NMR spectrum of compound LR 28 – 90 or rutin in methanol – d_4.
Figure S14. HMBC NMR spectrum of compound LR 28 – 90 or rutin in methanol – d_4.
Figure S15. Comparison of ^{1}H NMR spectrum of compound LR 27 – 98 and LR 28- 90 in methanol – d_4.
Figure S16. ^{1}H NMR spectrum of compound LR 665 or artabotryside A in methanol – d_4.
Figure S17. ^{13}C NMR spectrum of compound LR 665 or artabotryside A in methanol – d_4.
Figure S18. DEPT NMR spectrum of compound LR 665 or artabotryside A in methanol – d_4.
Figure S19. COSY NMR spectrum of compound LR 665 or artabotryside A in methanol – d_4.
Figure S20. HSQC NMR spectrum of compound LR 665 or artabotryside A in methanol – d_4.
Figure S21. HMBC spectrum of compound LR 665 or artabotryside A in methanol – d_4.
Figure S22. IR spectrum of compound LR 24-61 or hecpatrin.
Figure S23. MS spectrum of compound LR 24 – 61 or hecpatrin.
Figure S24. ^{1}H NMR spectrum of compound LR 24 – 61 or hecpatrin in methanol – d_4.
Figure S25. ^{13}C NMR spectrum of compound LR 24 – 61 or hecpatrin in methanol – d_4.
Figure S26. DEPT NMR spectrum of compound LR 24 – 61 or hecpatrin in methanol – d_4.

Figure S27. COSY NMR spectrum of compound LR 24 – 61 or hecpatrin in methanol – *d*$_4$.
Figure S28. HSQC NMR spectrum of compound LR 24 – 61 or hecpatrin in methanol – *d*$_4$.
Figure S29. HMBC NMR spectrum of compound LR 24 – 61 or hecpatrin in methanol – *d*$_4$.
Figure S30. NOESY NMR spectrum of compound LR 24 – 61 or hecpatrin in methanol – *d*$_4$.
Figure S31. ROESY NMR spectrum of compound LR 24 – 61 or hecpatrin in methanol – *d*$_4$.
Figure S32. TOCSY NMR spectrum of compound LR 24 – 61 or hecpatrin in methanol – *d*$_4$.
Figure S33. Cromatogram of compound LR 24 – 61 or hecpatrin in methanol – *d*$_4$.
Figure S34. IR spectrum of compound LR 24-57 or gaiadendrin.
Figure S35. MS spectrum of compound LR 24 – 57 or gaiadendrin.
Figure S36. ^{1}H NMR spectrum of compound LR 24 – 57 or gaiadendrin in methanol – *d*$_4$.
Figure S37. ^{13}C NMR spectrum of compound LR 24 – 57 or gaiadendrin in methanol – *d*$_4$.
Figure S38. DEPT NMR spectrum of compound LR 24 – 57 or gaiadendrin in methanol – *d*$_4$.
Figure S39. COSY NMR spectrum of compound LR 24 – 57 or gaiadendrin in methanol – *d*$_4$.
Figure S40. HSQC NMR spectrum of compound LR 24 – 57 or gaiadendrin in methanol – *d*$_4$.
Figure S41. HMBC spectrum of compound LR 24 – 57 or gaiadendrin in methanol – *d*$_4$.
Figure S42. NOESY spectrum of compound LR 24 – 57 or gaiadendrin in deuterium oxide.
Figure S43. ROESY spectrum of compound LR 24 – 57 or gaiadendrin in deuterium oxide.
Figure S44. TOCSY spectrum of compound LR 24 – 57 or gaiadendrin in deuterium oxide.
Figure S45. Cromatogram of compound LR 24 – 57 or gaiadendrin.
Figure S46. IR spectrum of compound LR 29-15 or puchikrin.
Figure S47. MS spectrum of compound LR 29 – 15 or puchikrin.
Figure S48. ^{1}H NMR spectrum of compound LR 29 – 15 or puchikrin in methanol – *d*$_4$.
Figure S49. ^{13}C NMR spectrum of compound LR 29 – 15 or puchikrin in methanol – *d*$_4$.
Figure S50. DEPT NMR spectrum of compound LR 29 – 15 or puchikrin in methanol – *d*$_4$.
Figure S51. COSY NMR spectrum of compound LR 29 – 15 or puchikrin in methanol – *d*$_4$.
Figure S52. HSQC NMR spectrum of compound LR 29 – 15 or puchikrin in methanol – *d*$_4$.

Figure S53. HMBC NMR spectrum of compound LR 29 – 15 or puchikrin in methanol – d_4.
Figure S54. NOESY NMR spectrum of compound LR 29 – 15 or puchikrin in deuterium oxide.
Figure S55. ROESY NMR spectrum of compound LR 29 – 15 or puchikrin in deuterium oxide.
Figure S56. TOCSY NMR spectrum of compound LR 29 – 15 or puchikrin in deuterium oxide.
Figure S57. Cromatogram of compound LR 29 – 15 or puchikrin.
Figure S58. Comparison of ^{1}H NMR spectrum of compound LR 24 – 57 and LR 29- 15 in deuterium oxide at 20°C.
Figure S59. Comparison of ^{1}H NMR spectrum of compound LR 24 – 57 and LR 29- 15 in deuterium oxide at 30°C.
Figure S60. Comparison of ^{1}H NMR spectrum of compound LR 24 – 57 and LR 29- 15 in deuterium oxide at 40°C.

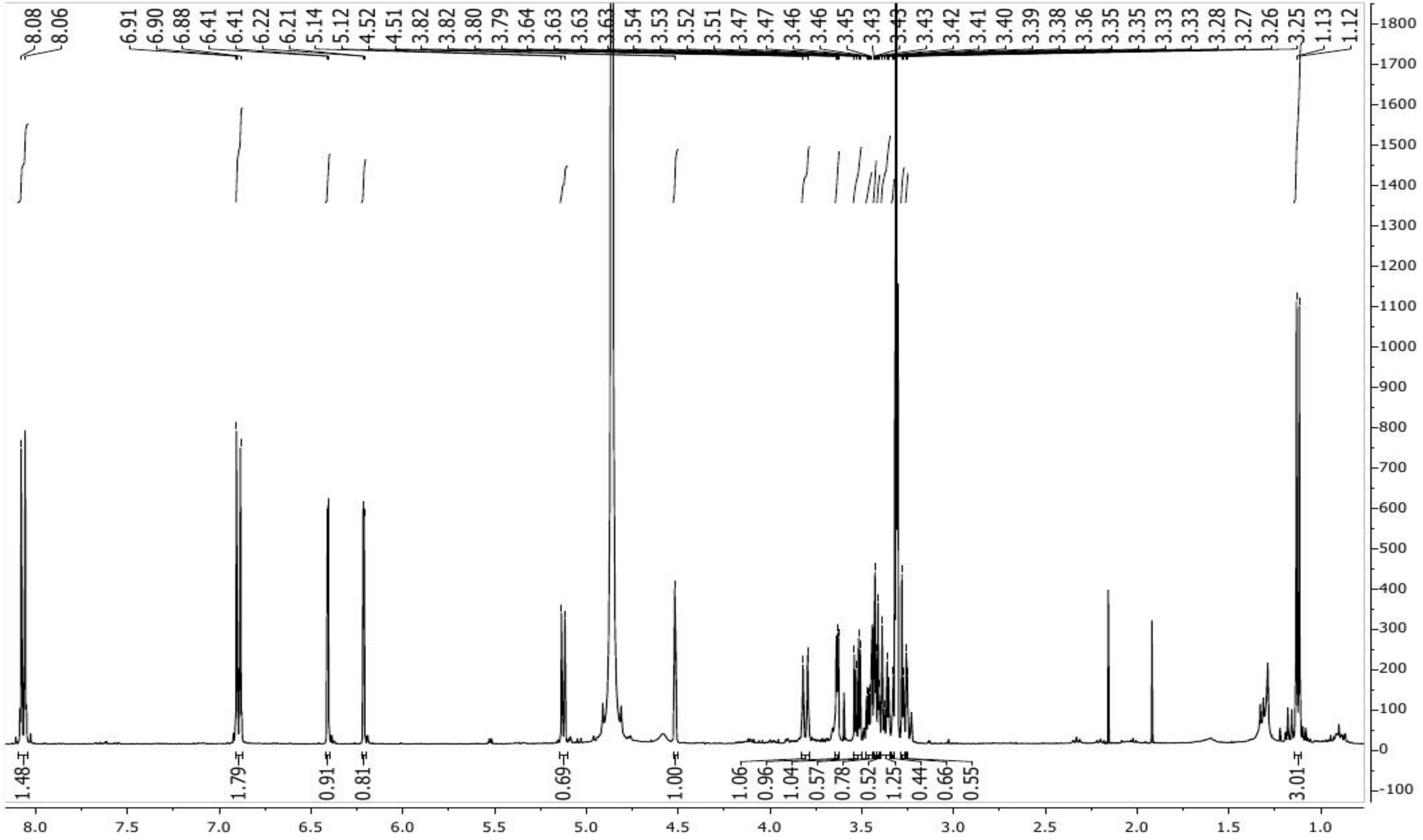

Figure S1. ^{1}H NMR spectrum of compound LR 29 – 13 or nicotiflorin in methanol – d_4.

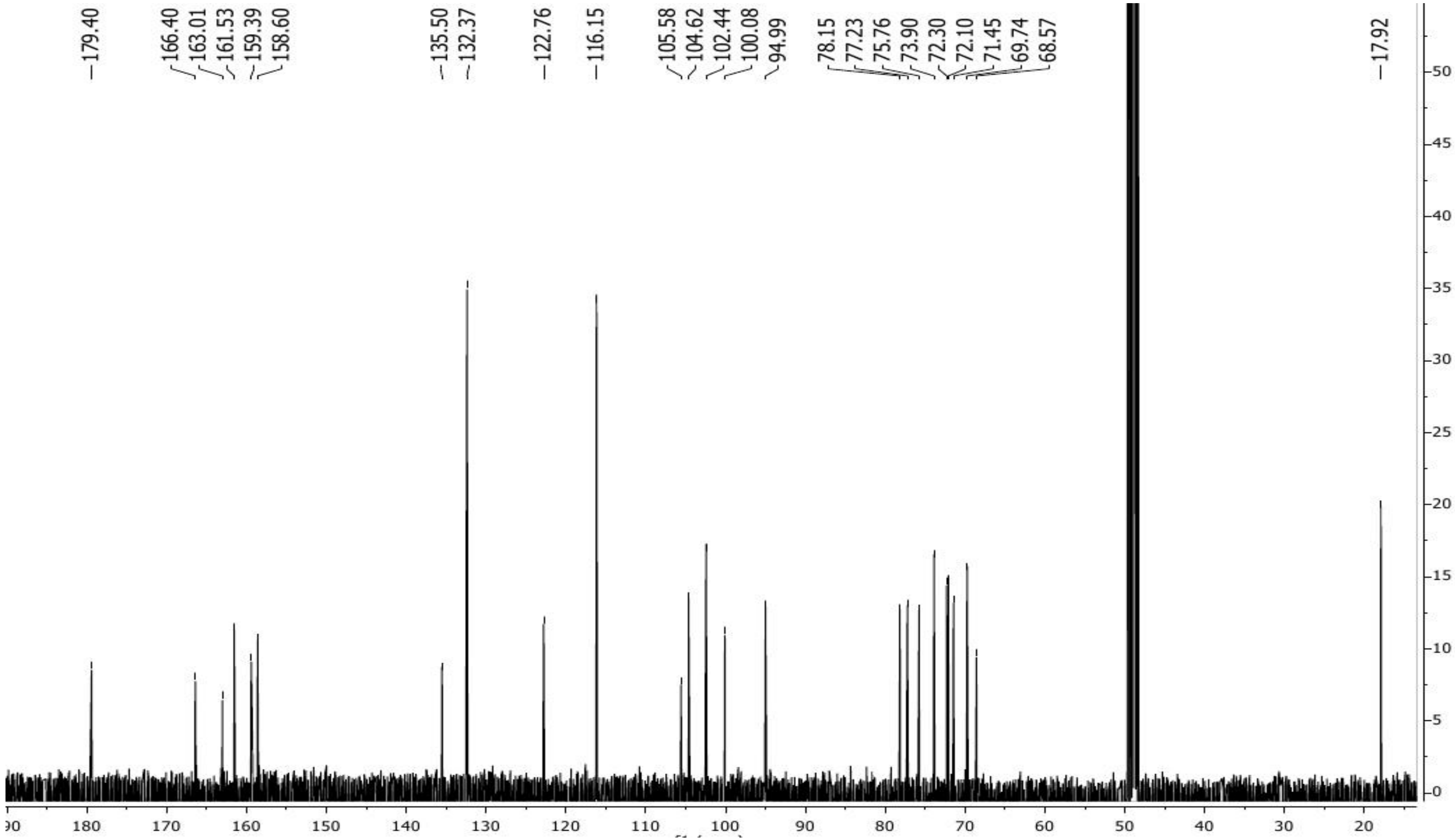

Figure S2. ^{13}C NMR spectrum of compound LR 29 – 13 or nicotiflorin in methanol – d_4.

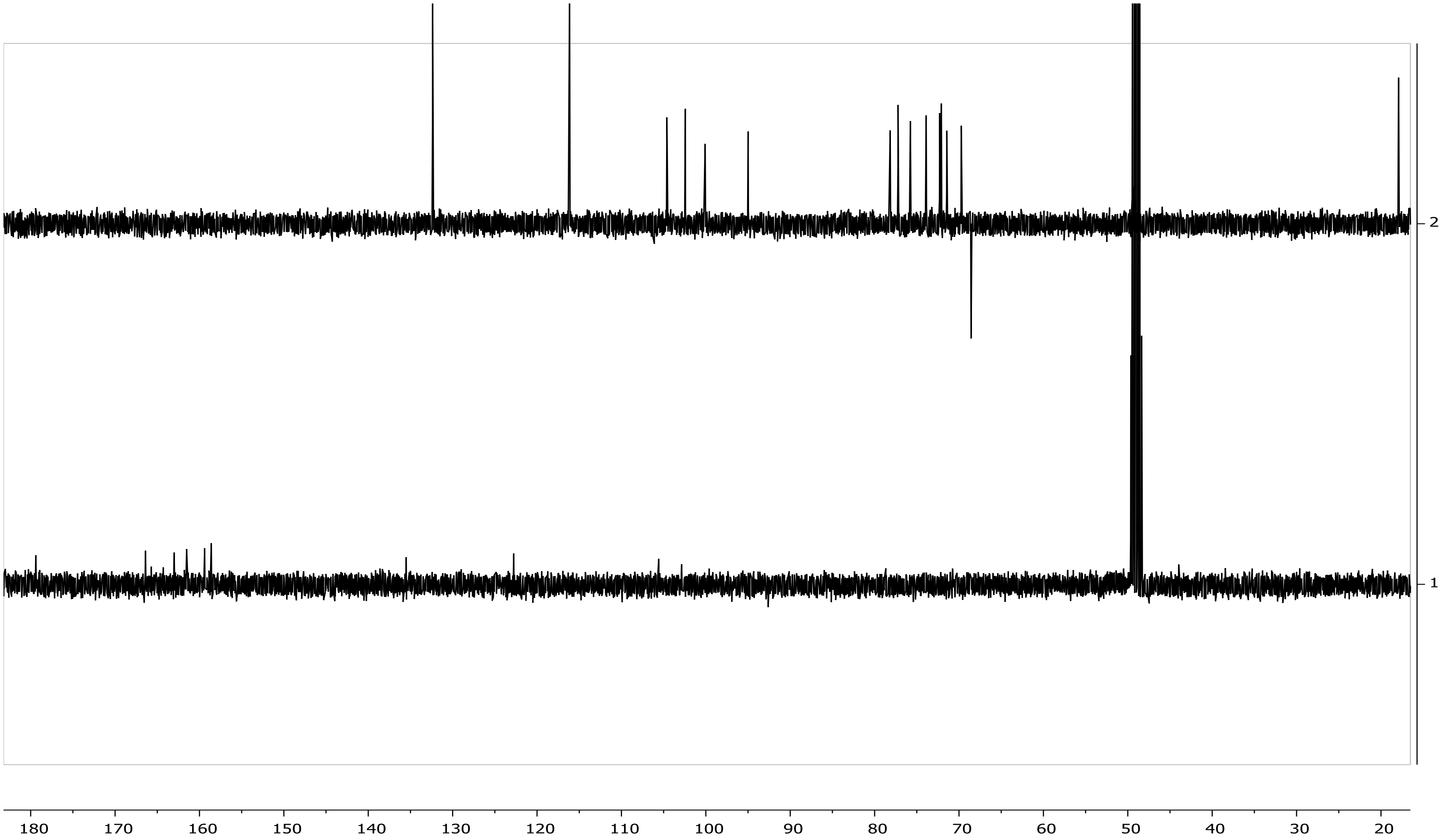

Figure S3. DEPT NMR spectrum of compound LR 29 – 13 or nicotiflorin in methanol – *d_4*.

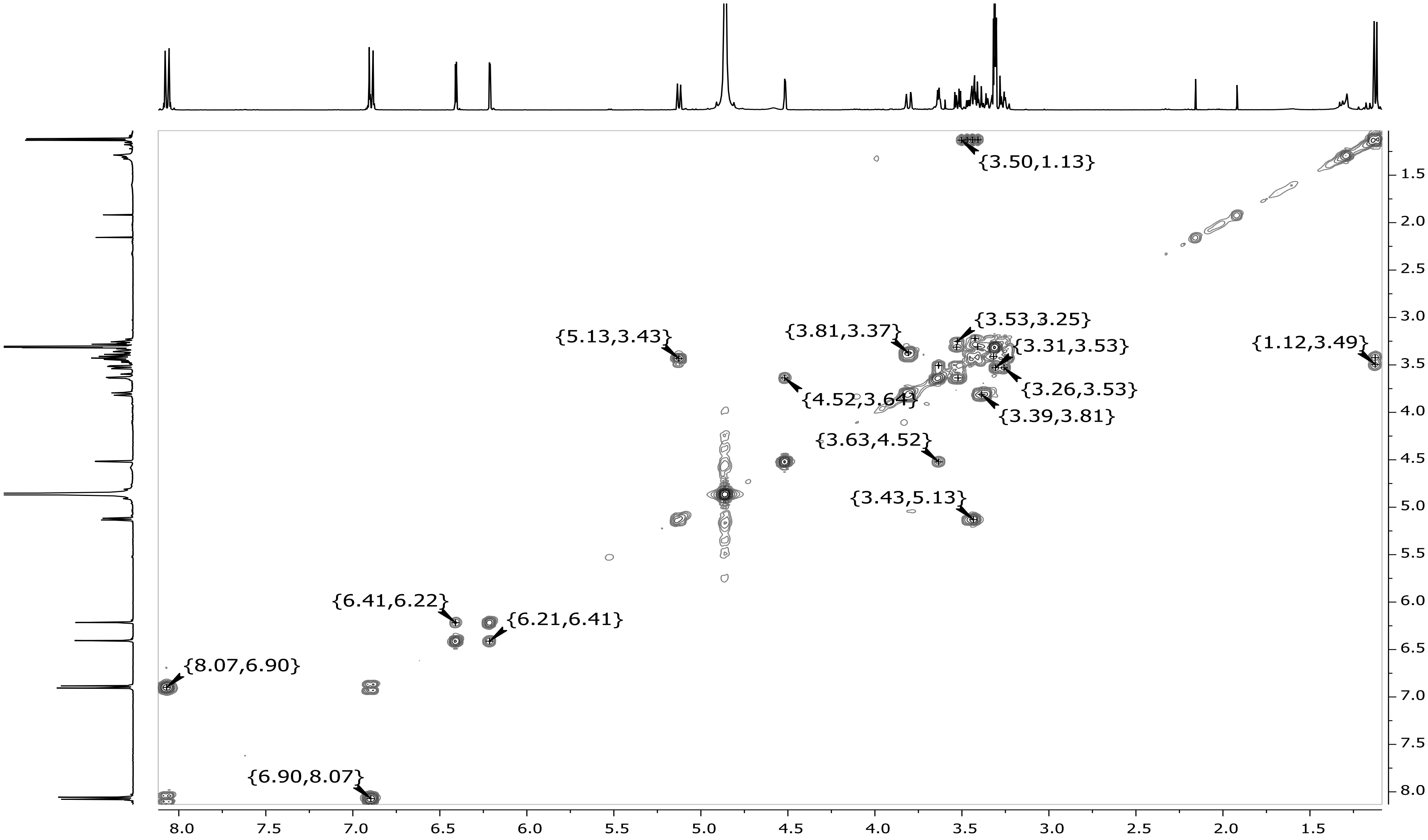

Figure S4. COSY NMR spectrum of compound LR 29 – 13 or nicotiflorin in methanol – d_4.

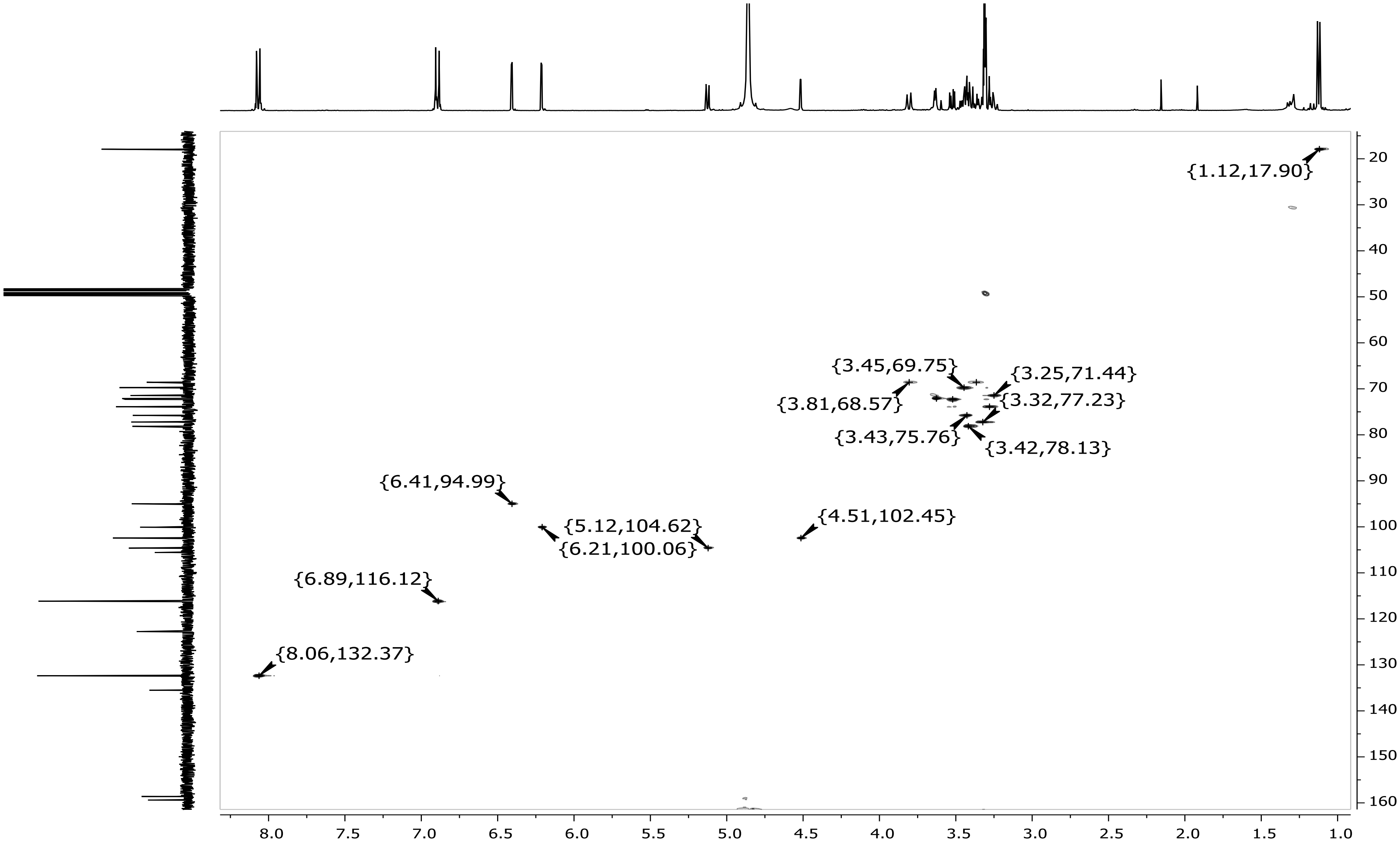

Figure S5. HSQC NMR spectrum of compound LR 29 – 13 or nicotiflorin in methanol – d_4.

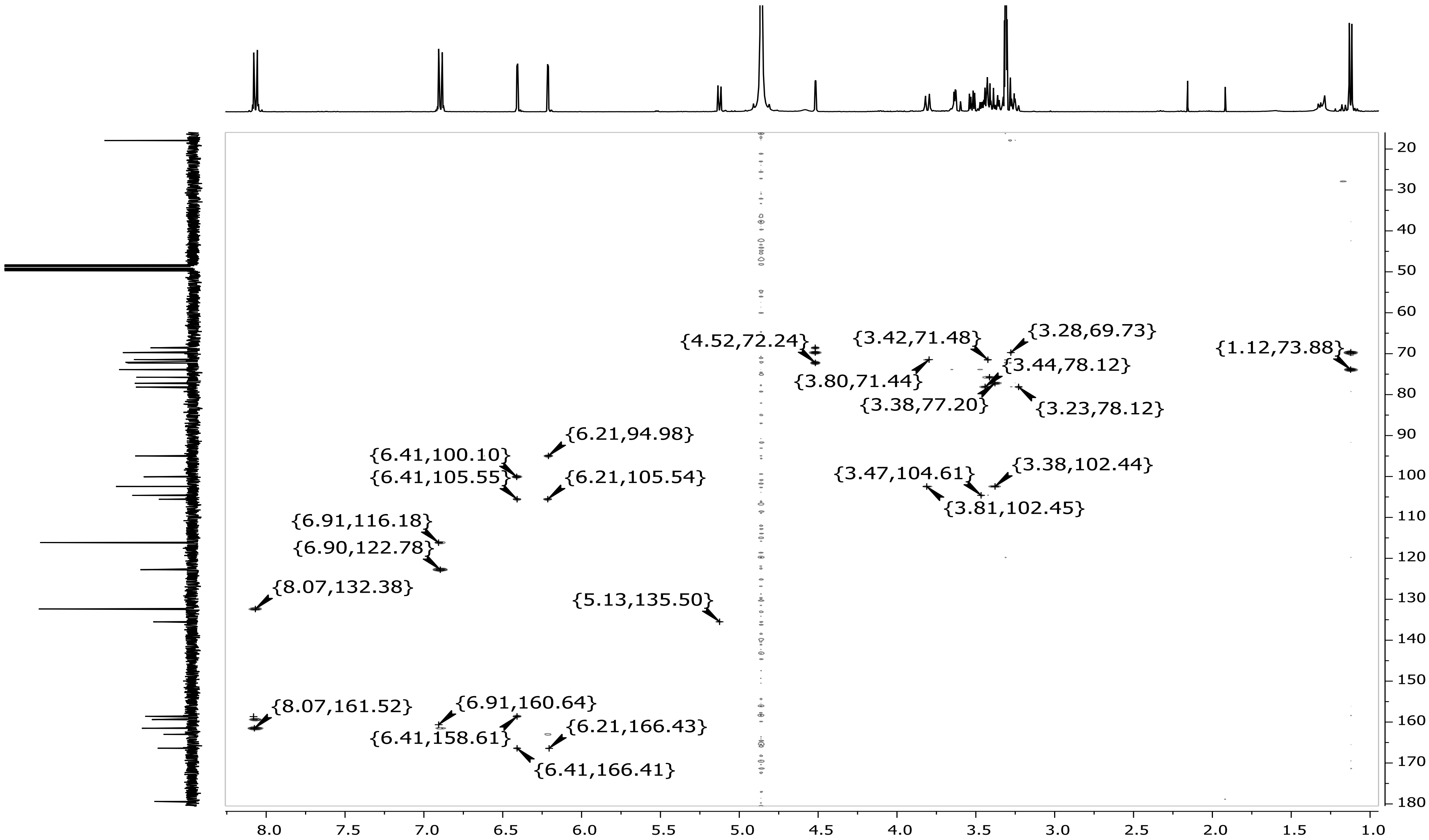

Figure S6. HMBC spectrum of compound LR 29 – 13 or nicotiflorin in methanol – d_4.

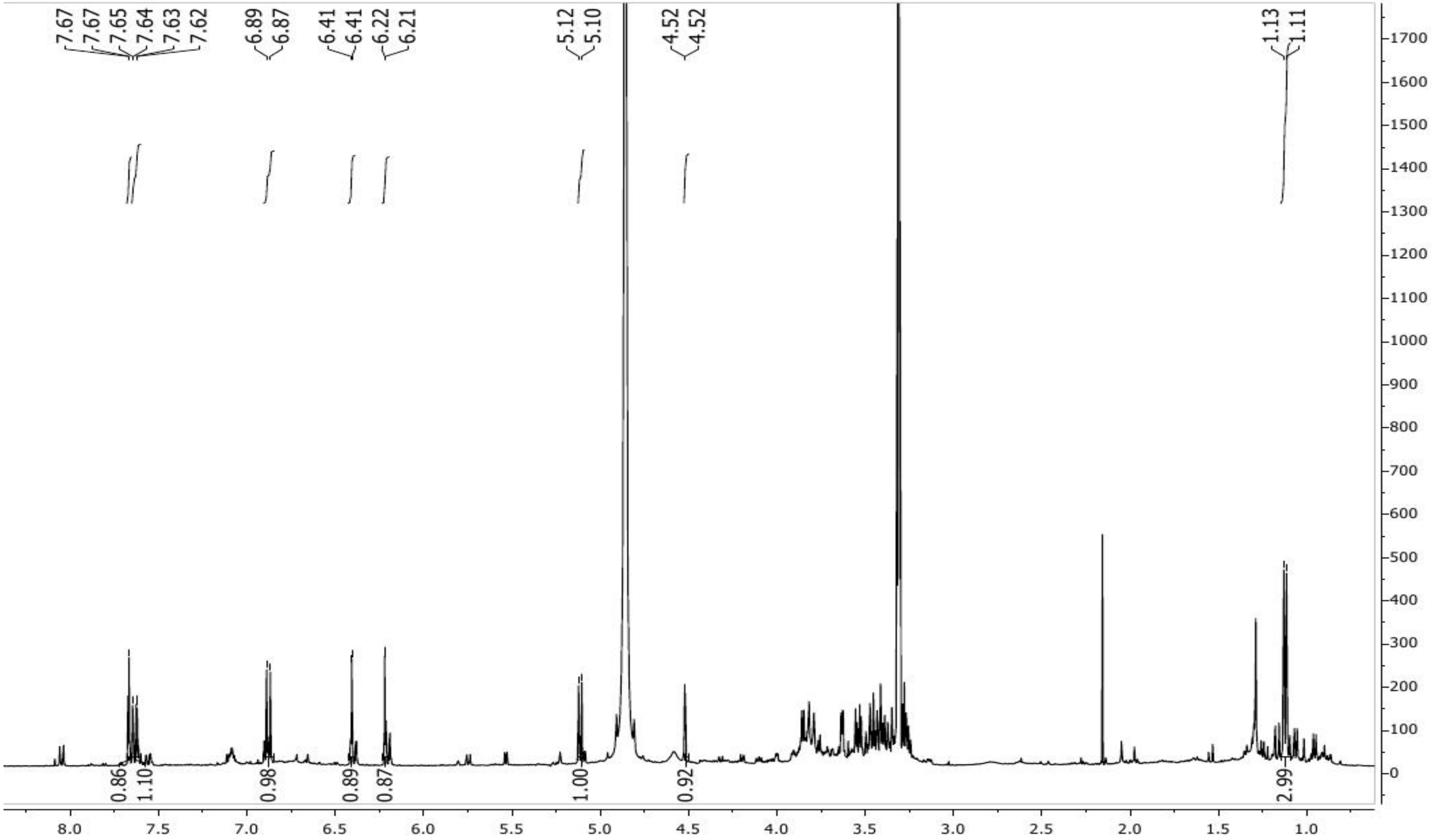

Figure S7. ^{1}H NMR spectrum of compound LR 27 – 98 or rutin in methanol – d_4.

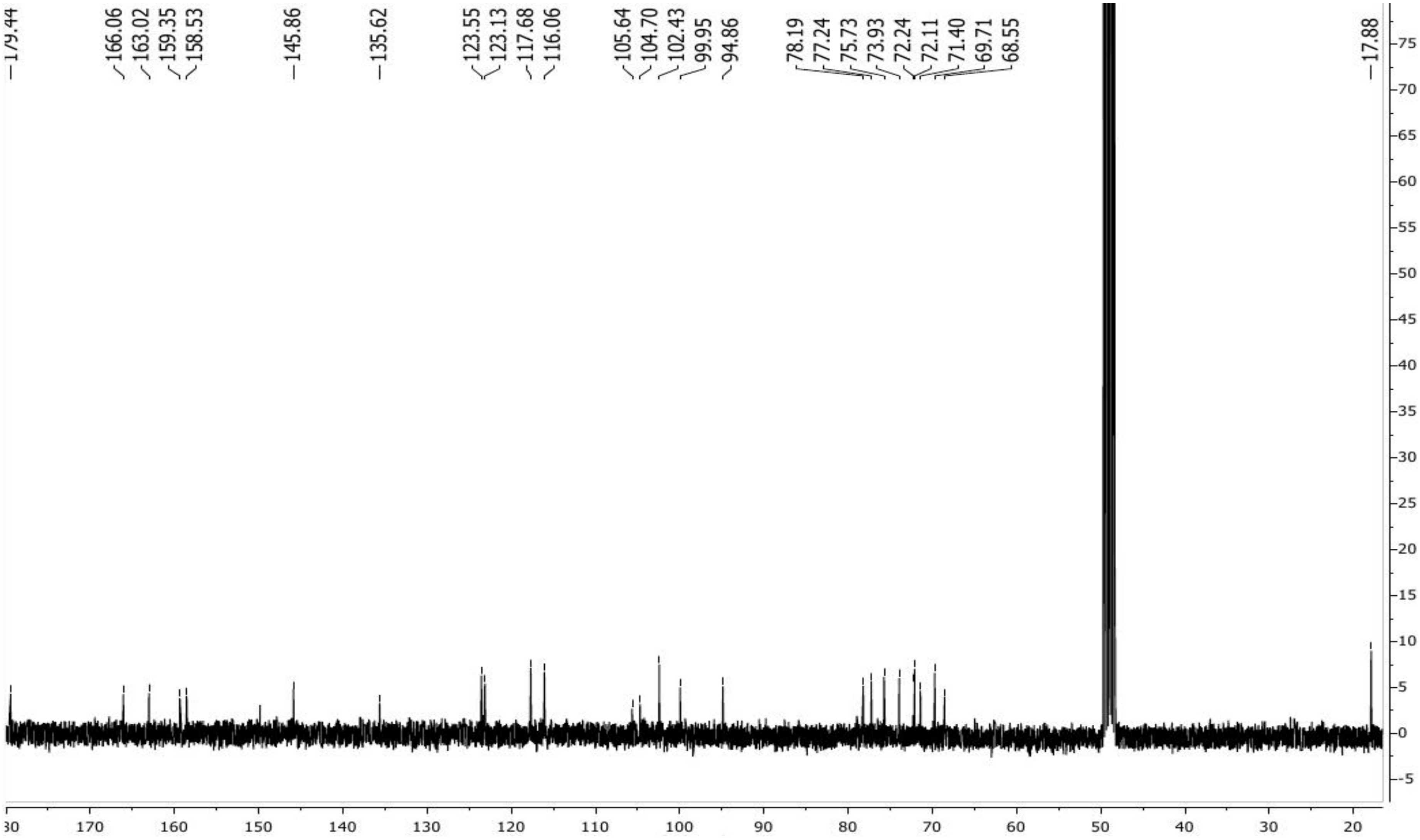

Figure S8. ^{13}C NMR spectrum of compound LR 27 – 98 or rutin in methanol – *d*$_4$.

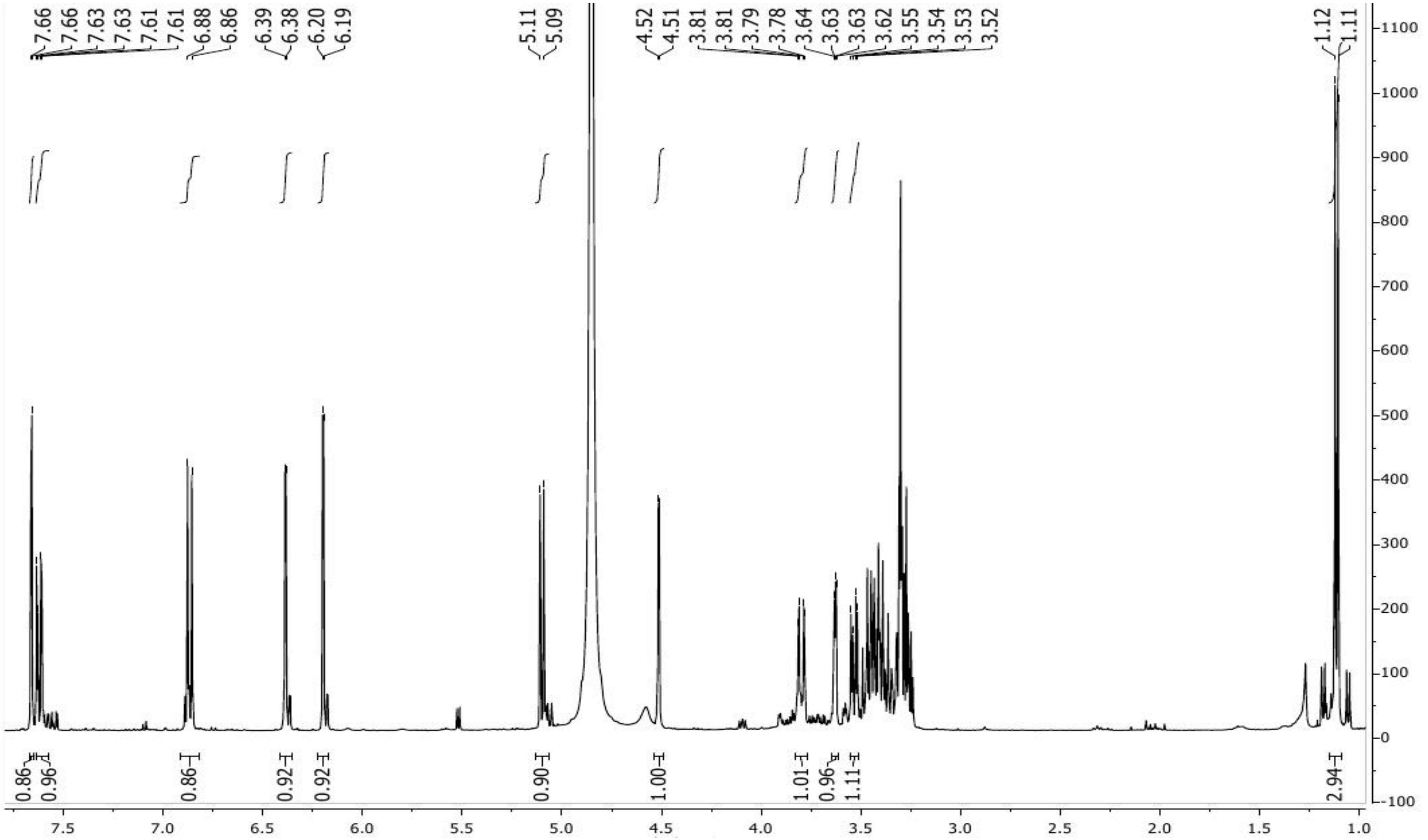

Figure S9. ^{1}H NMR spectrum of compound LR 28 – 90 or rutin in methanol – d_4.

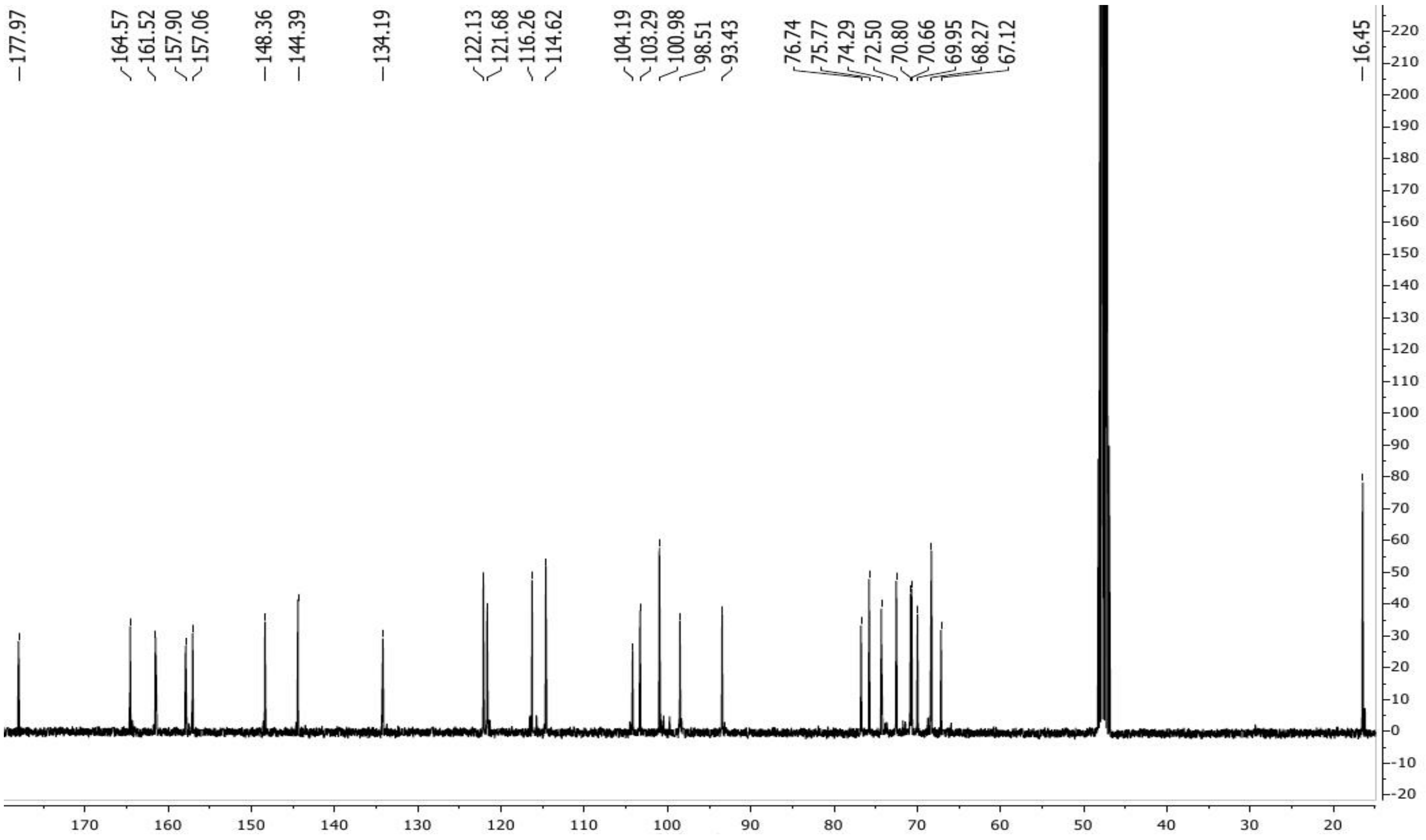

Figure S10. ^{13}C NMR spectrum of compound LR 28 – 90 or rutin in methanol – d_4.

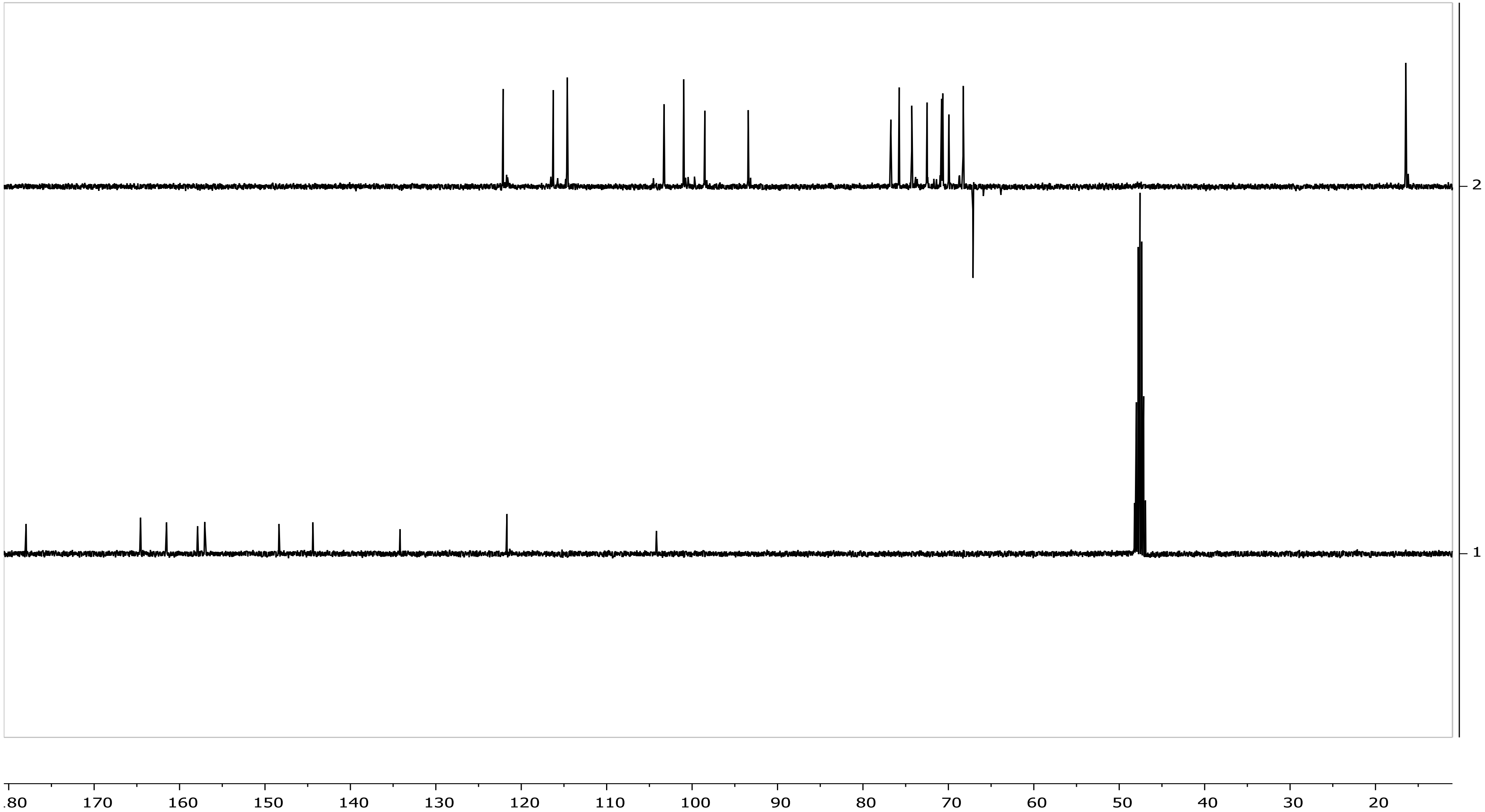

Figure S11. DEPT NMR spectrum of compound LR 28 – 90 or rutin in methanol – *d*$_4$.

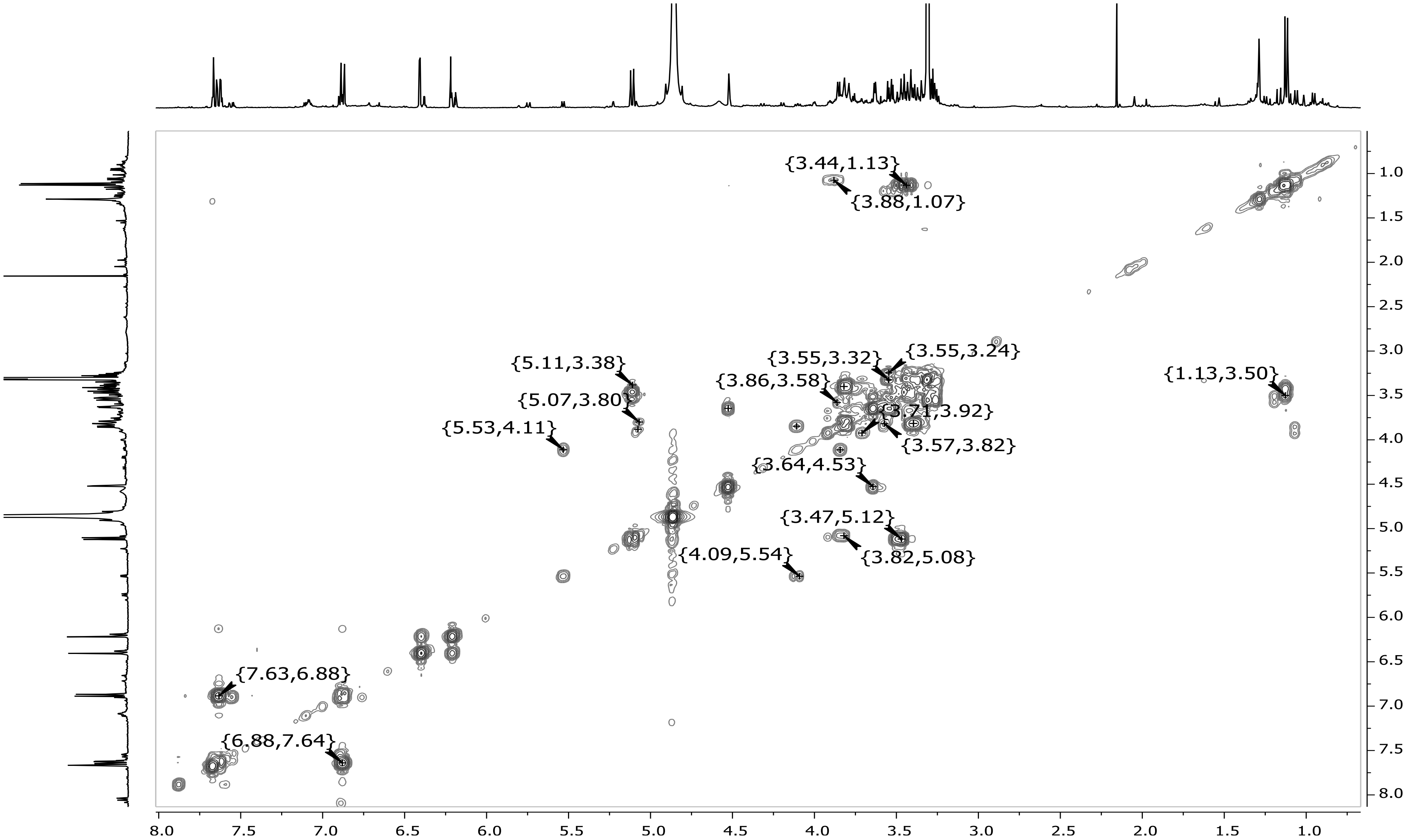

Figure S12. COSY NMR spectrum of compound LR 28 – 90 or rutin in methanol – *d*4.

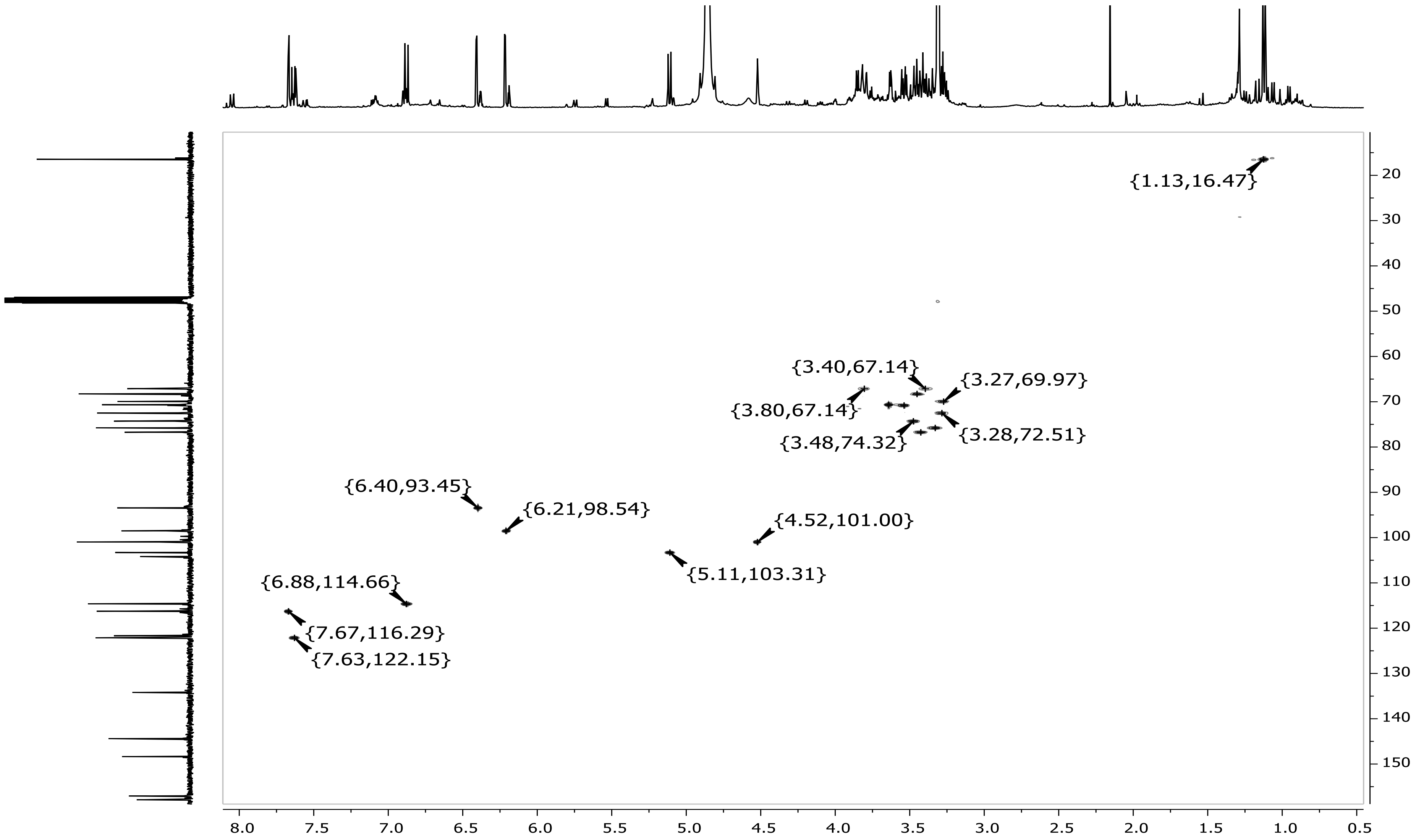

Figure S13. HSQC NMR spectrum of compound LR 28 – 90 or rutin in methanol – d_4.

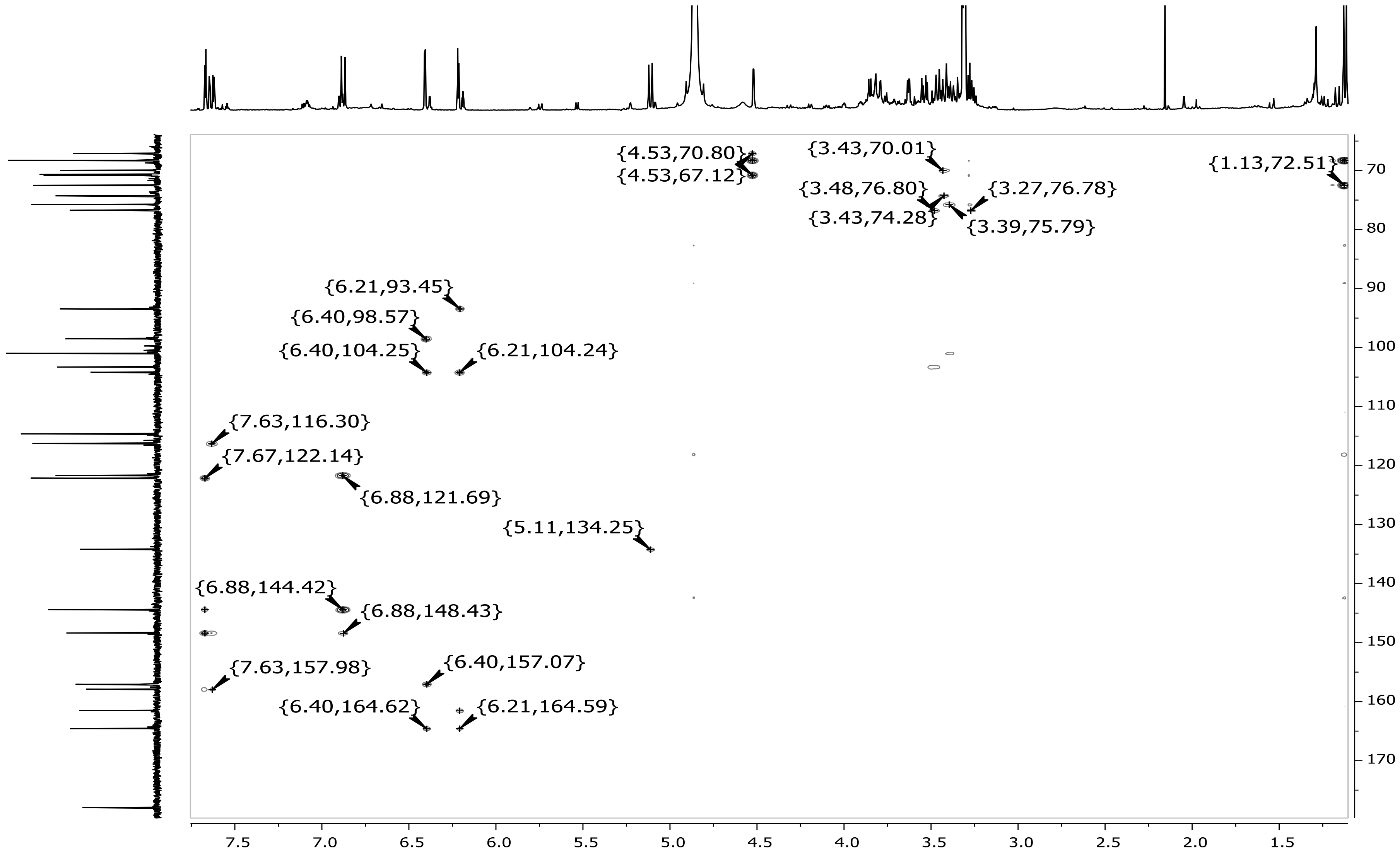

Figure S14. HMBC NMR spectrum of compound LR 28 – 90 or rutin in methanol – d_4.

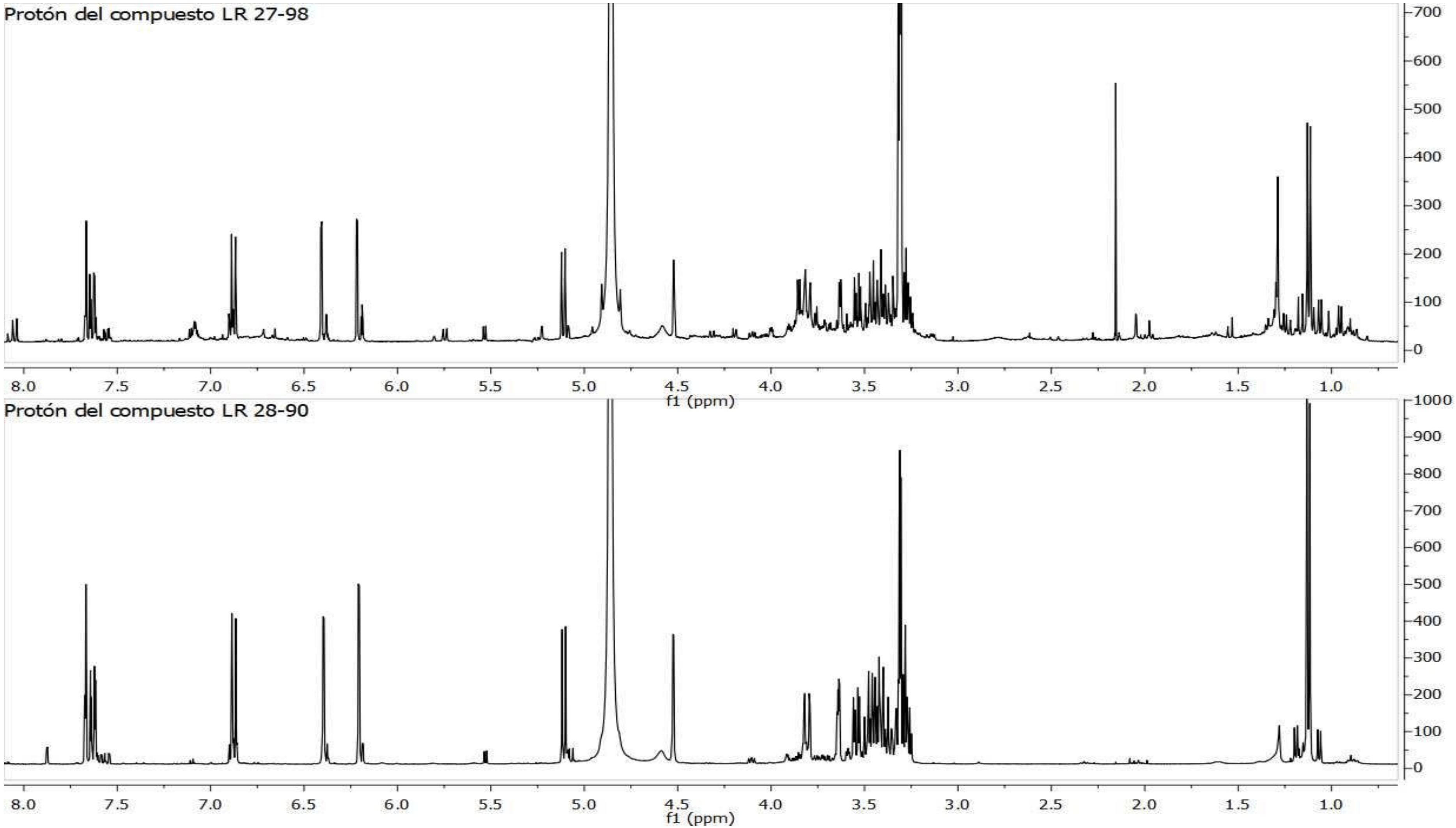

Figure S15. Comparison of ^{1}H NMR spectrum of compound LR 27 – 98 and LR 28- 90 in methanol – *d₄*.

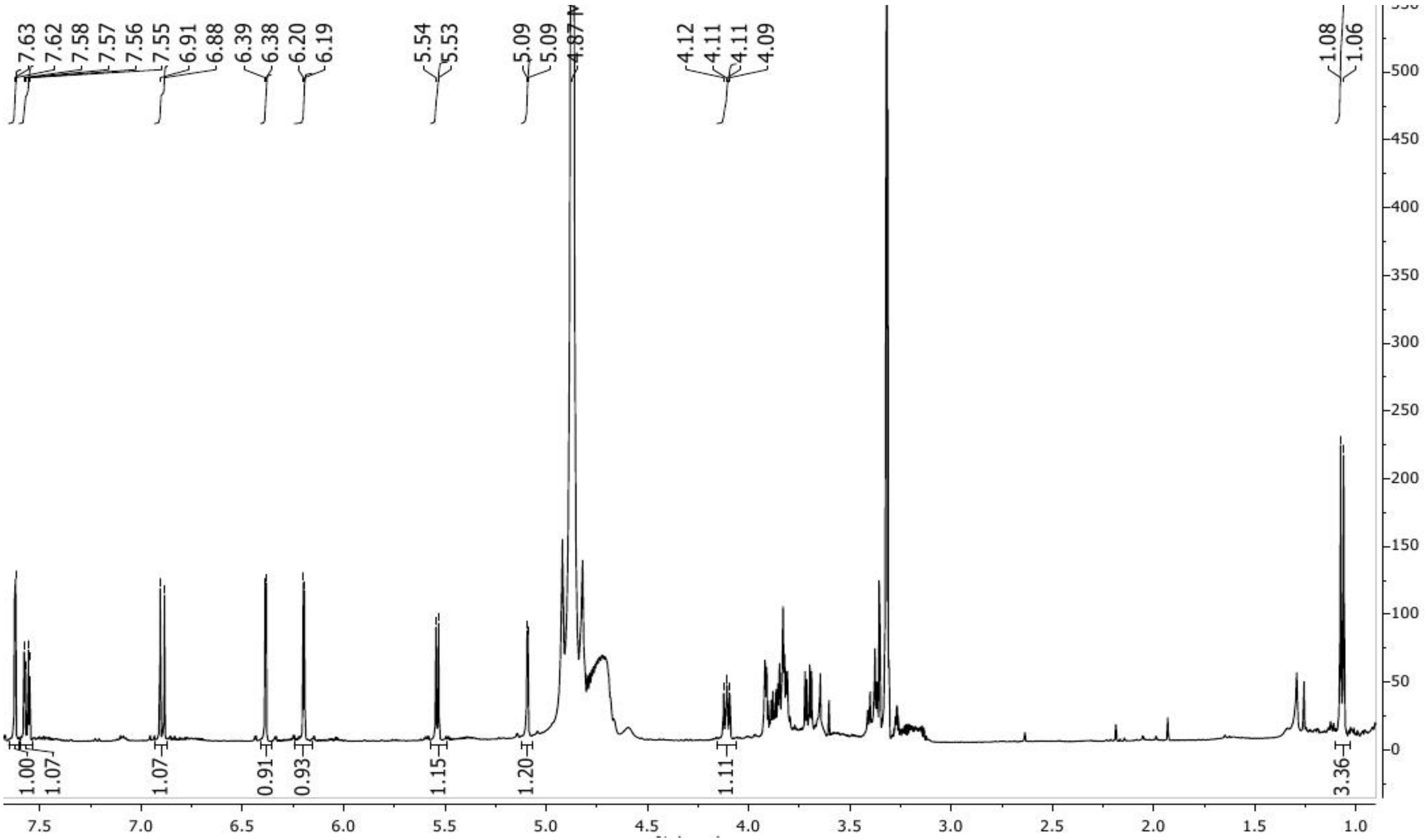

Figure S16. ^{1}H NMR spectrum of compound LR 665 or artabotryside A in methanol – d_4.

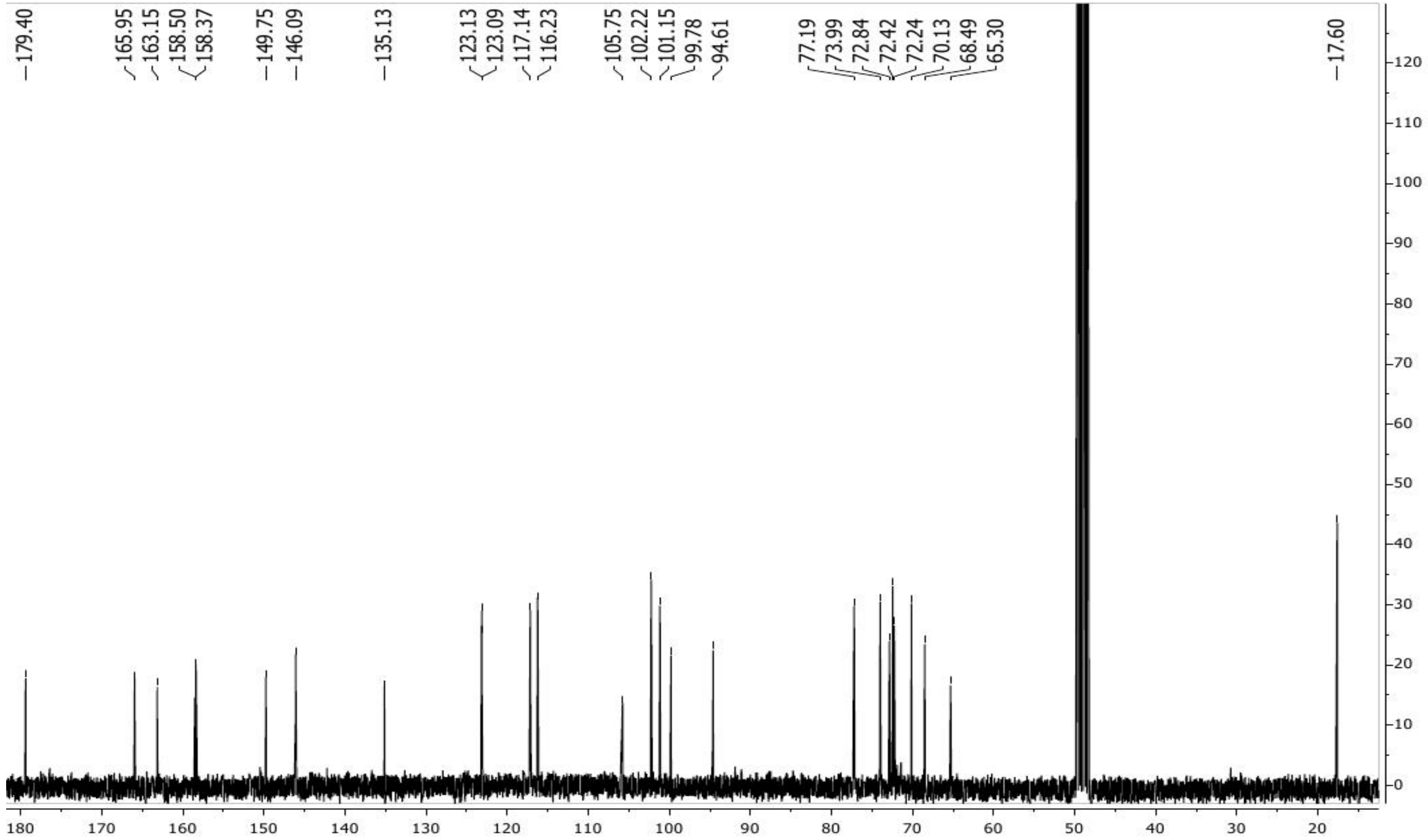

Figure S17. ^{13}C NMR spectrum of compound LR 665 or artabotryside A in methanol – d_4.

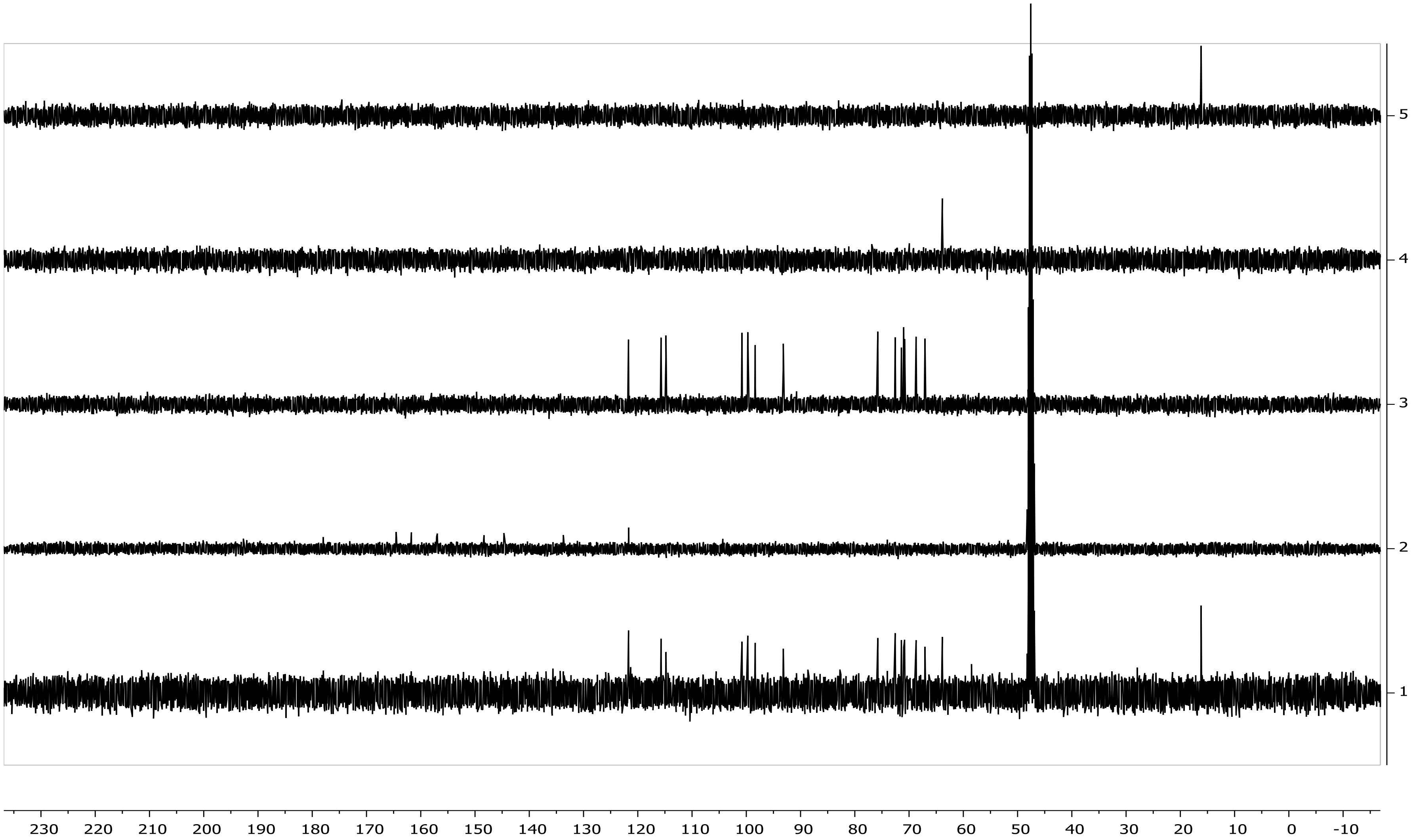

Figure S18. DEPT NMR spectrum of compound LR 665 or artabotryside A in methanol – d_4.

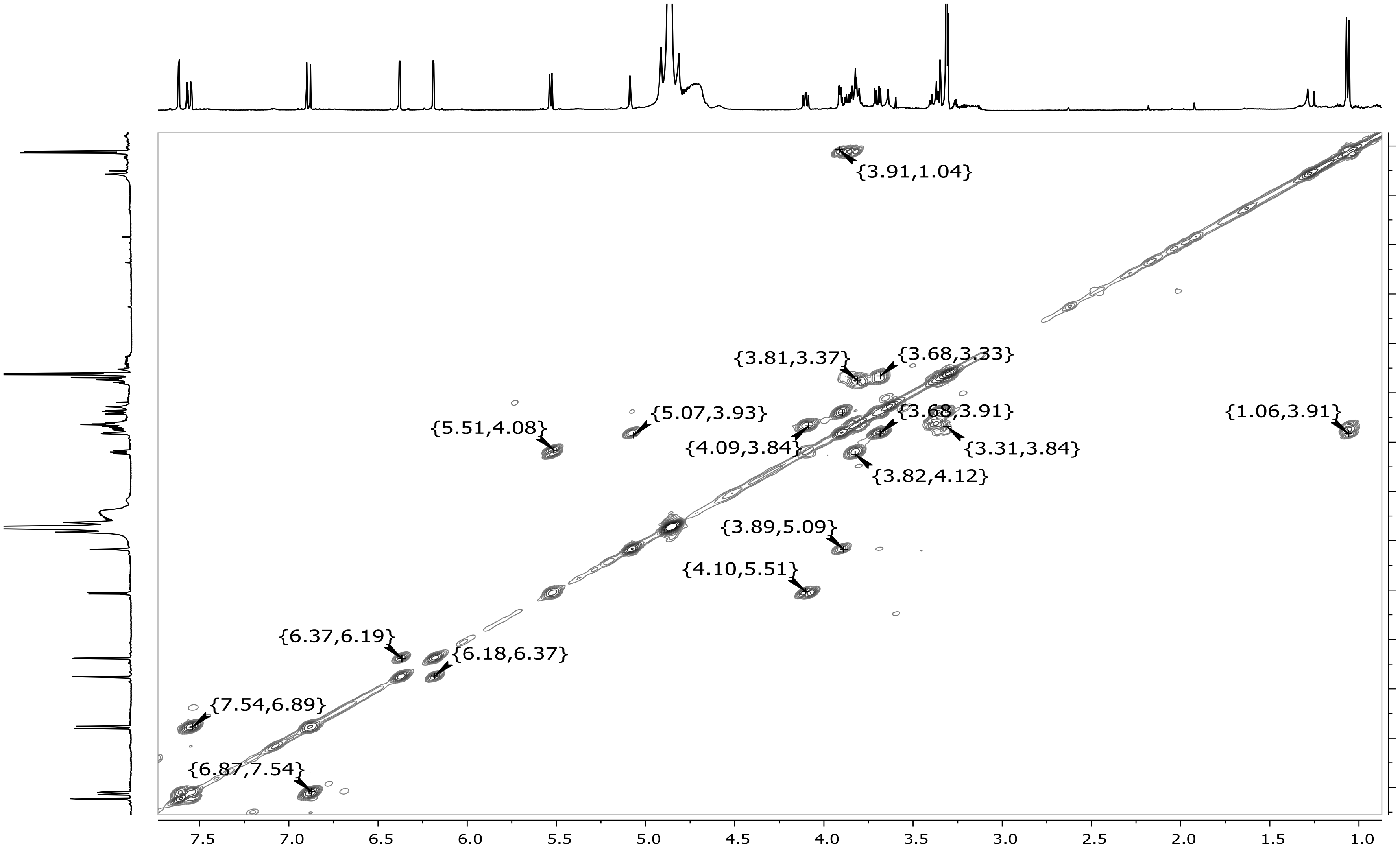

Figure S19. COSY NMR spectrum of compound LR 665 or artabotryside A in methanol – *d*$_4$.

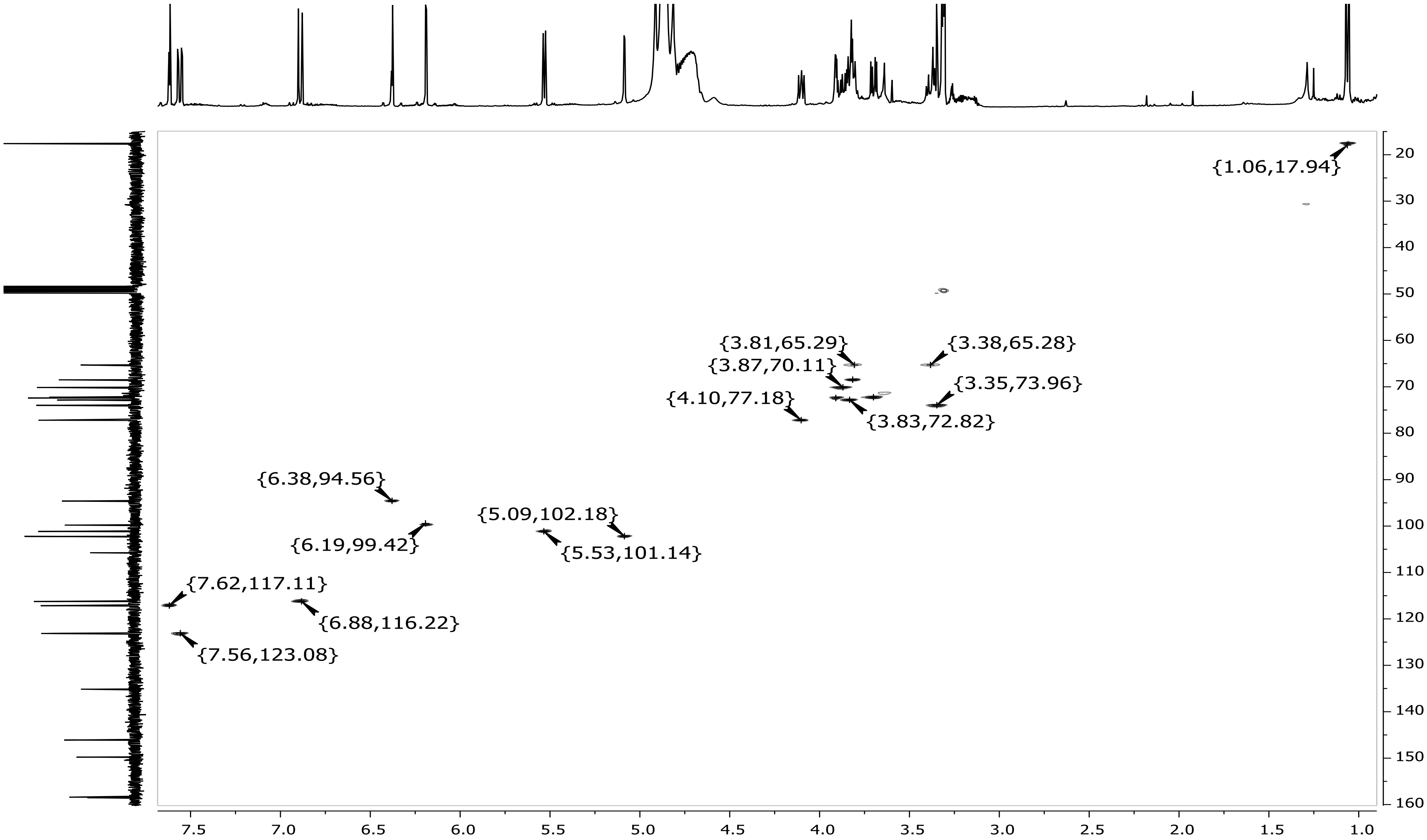

Figure S20. HSQC NMR spectrum of compound LR 665 or artabotryside A in methanol – d_4

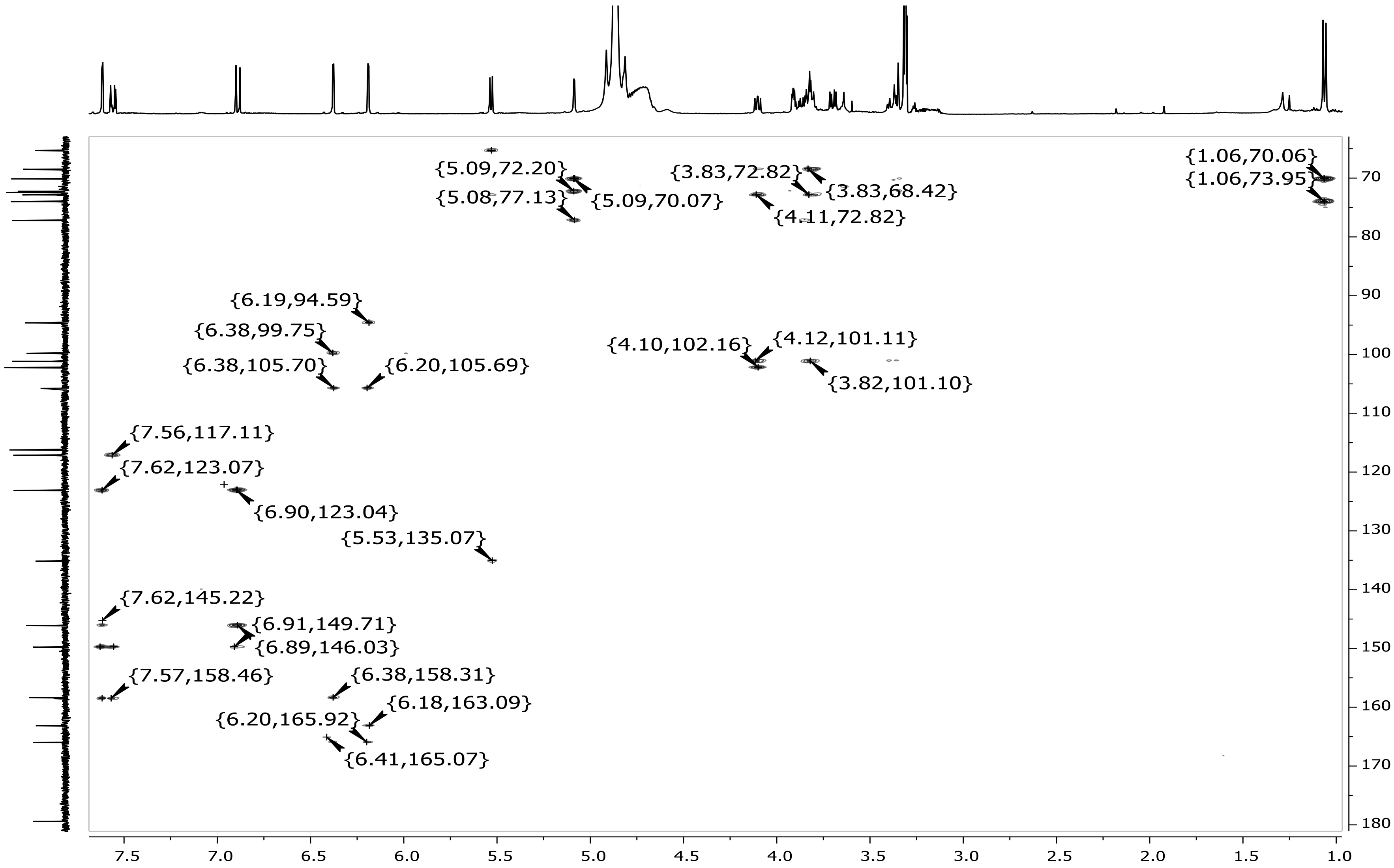

Figure S21. HMBC spectrum of compound LR 665 or artabotryside A in methanol – *d*4.

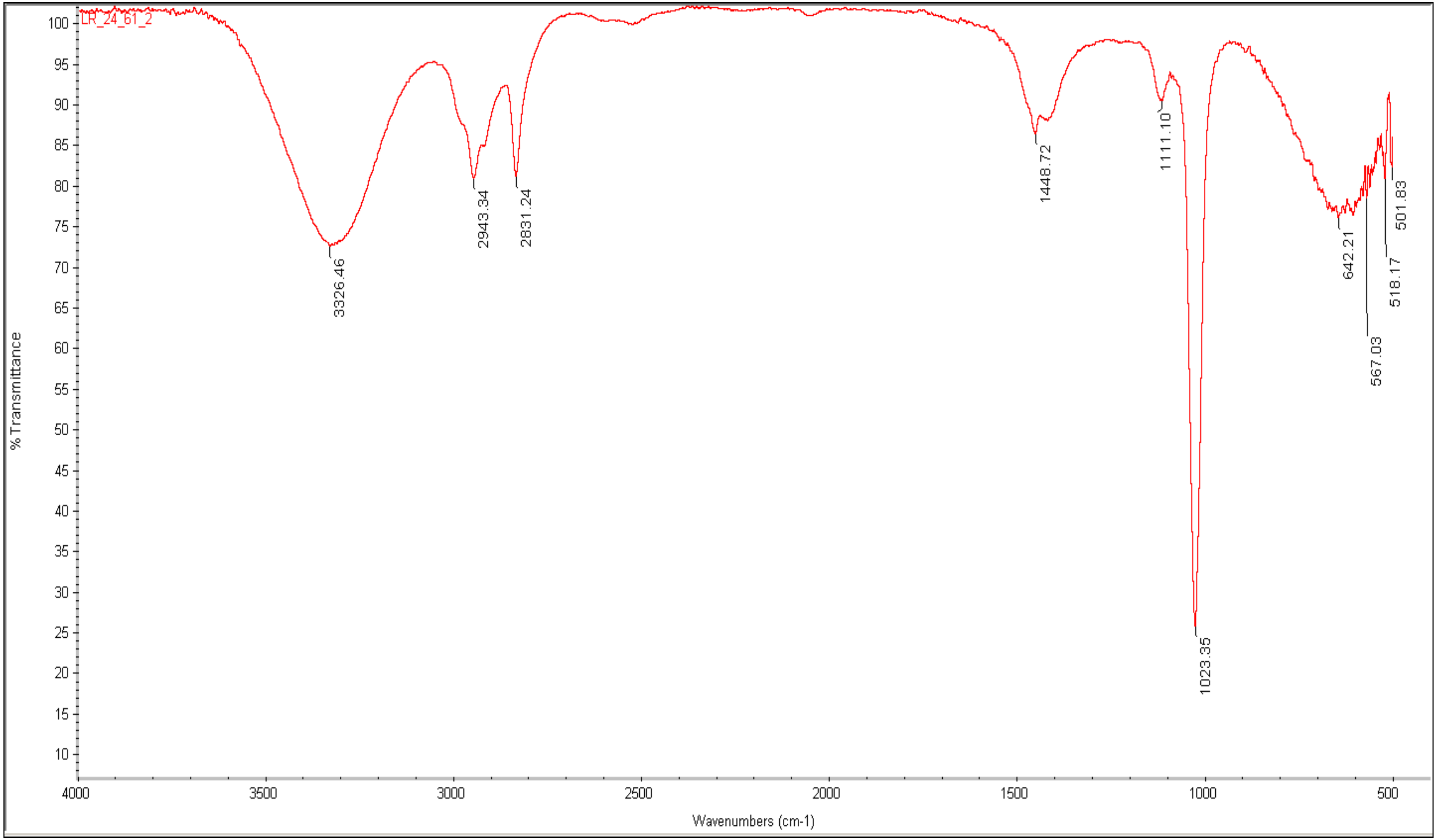

Figure S22. IR spectrum of compound LR 24-61 or hecpatrin

Mass Spectrum Deconvolution Report

Analysis Info

		Acquisition Date	4/8/2019 12:31:59 PM
Analysis Name	D:\Data\bruker enero18\Metodo 2018\PAULO CEDENO\LR_24_61_MS.d		
Method	paulo.m	Operator	BDAL@DE
Sample Name	LR_24_61_MS	Instrument	amaZon speed
Comment			

Acquisition Parameter

Ion Source Type	ESI	Ion Polarity	Positive	Alternating Ion Polarity	off
Mass Range Mode	UltraScan	Scan Begin	100 m/z	Scan End	1000 m/z
Accumulation Time	72845 µs	RF Level	63 %	Trap Drive	54.1
SPS Target Mass	400 m/z	Averages	5 Spectra	n/a	n/a

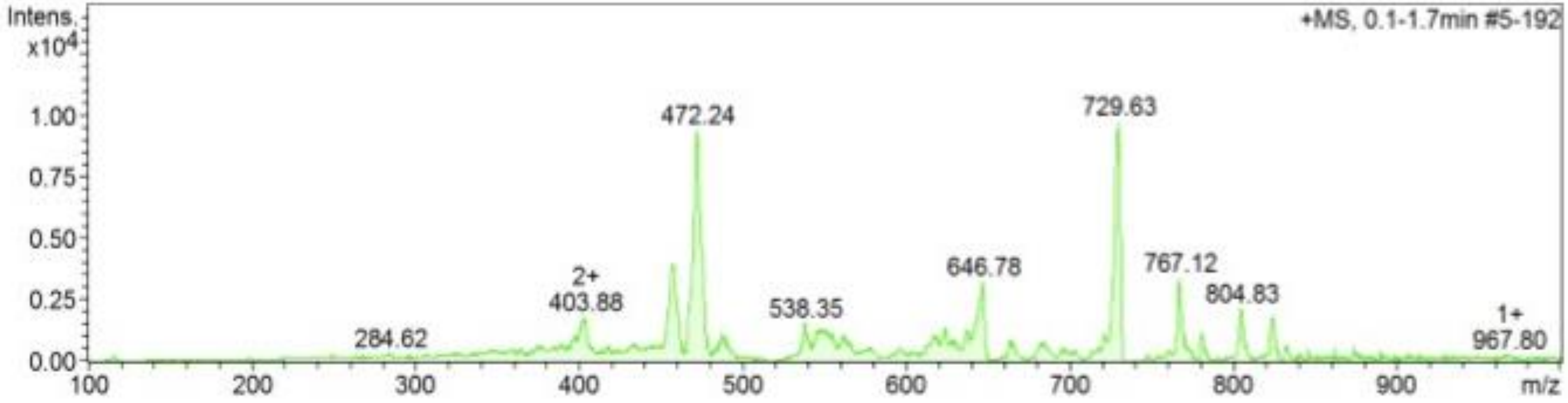

Figure S23. MS spectrum of compound LR 24 – 61 or hecpatrin

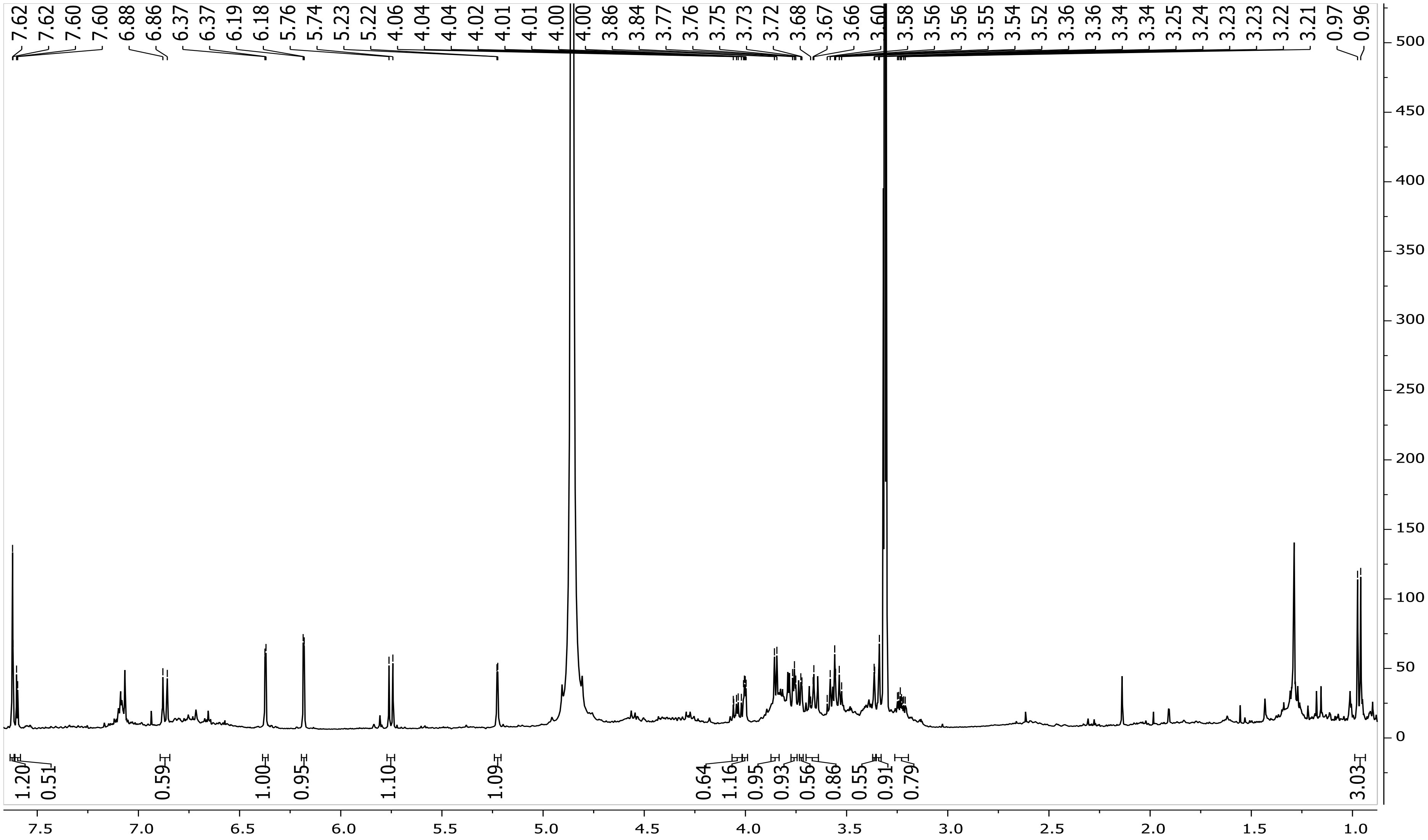

Figure S24. ^{1}H NMR spectrum of compound LR 24 – 61 or hecpatrin in methanol – d_4.

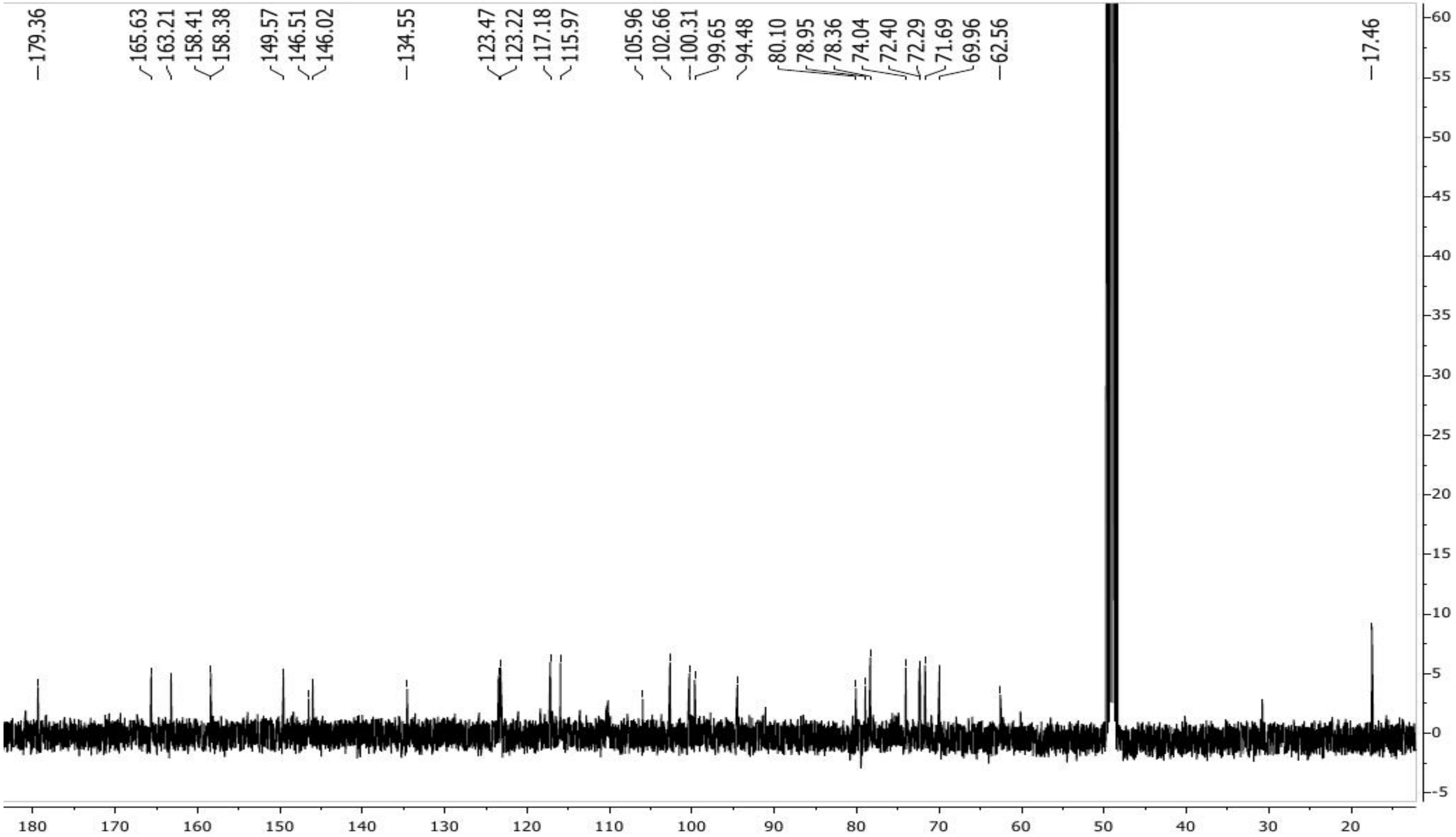

Figure S25. ^{13}C NMR spectrum of compound LR 24 – 61 or hecpatrin in methanol – d_4.

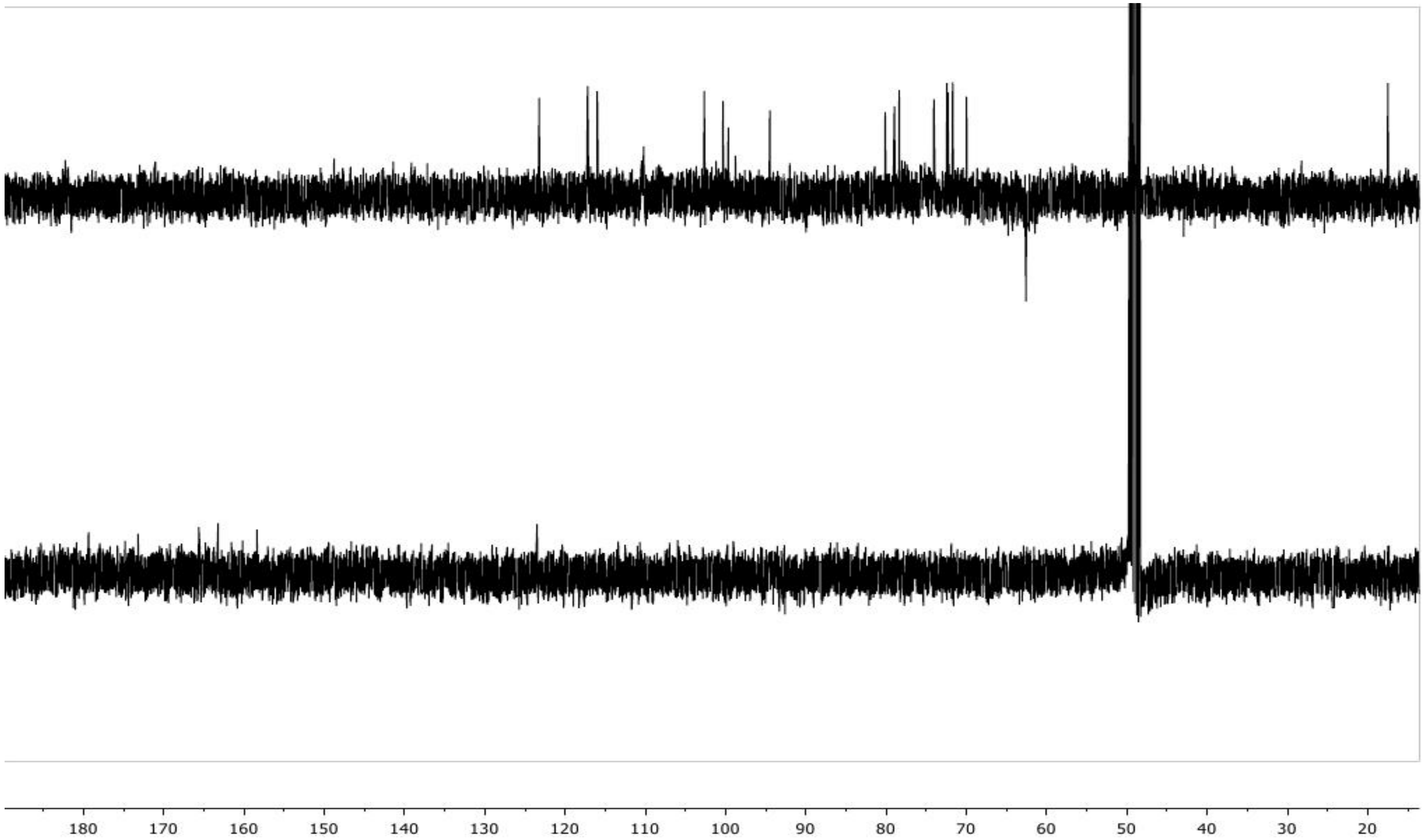

Figure S26. DEPT NMR spectrum of compound LR 24 – 61 or hecpatrin in methanol – d_4.

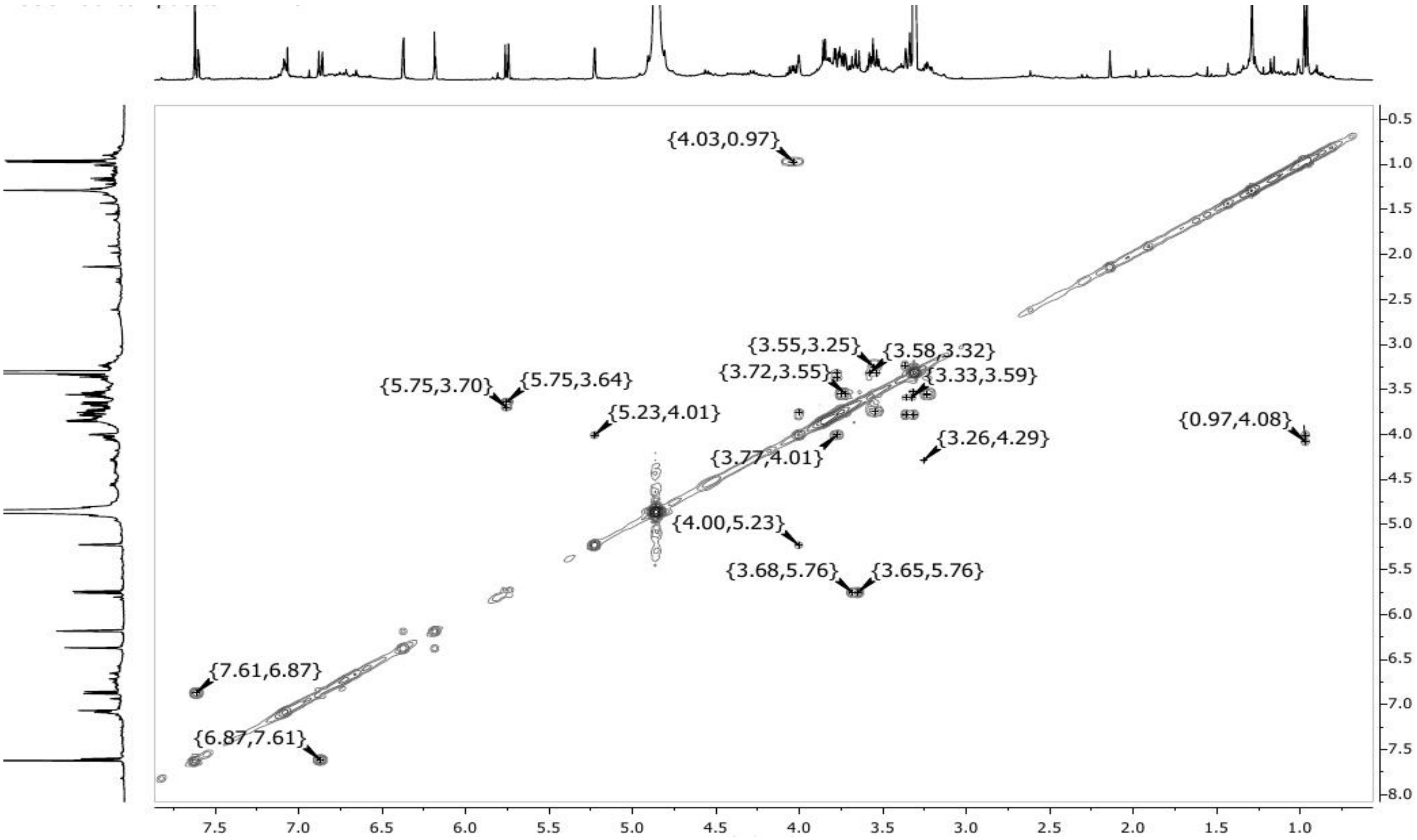

Figure S27. COSY NMR spectrum of compound LR 24 – 61 or hecpatrin in methanol – d_4.

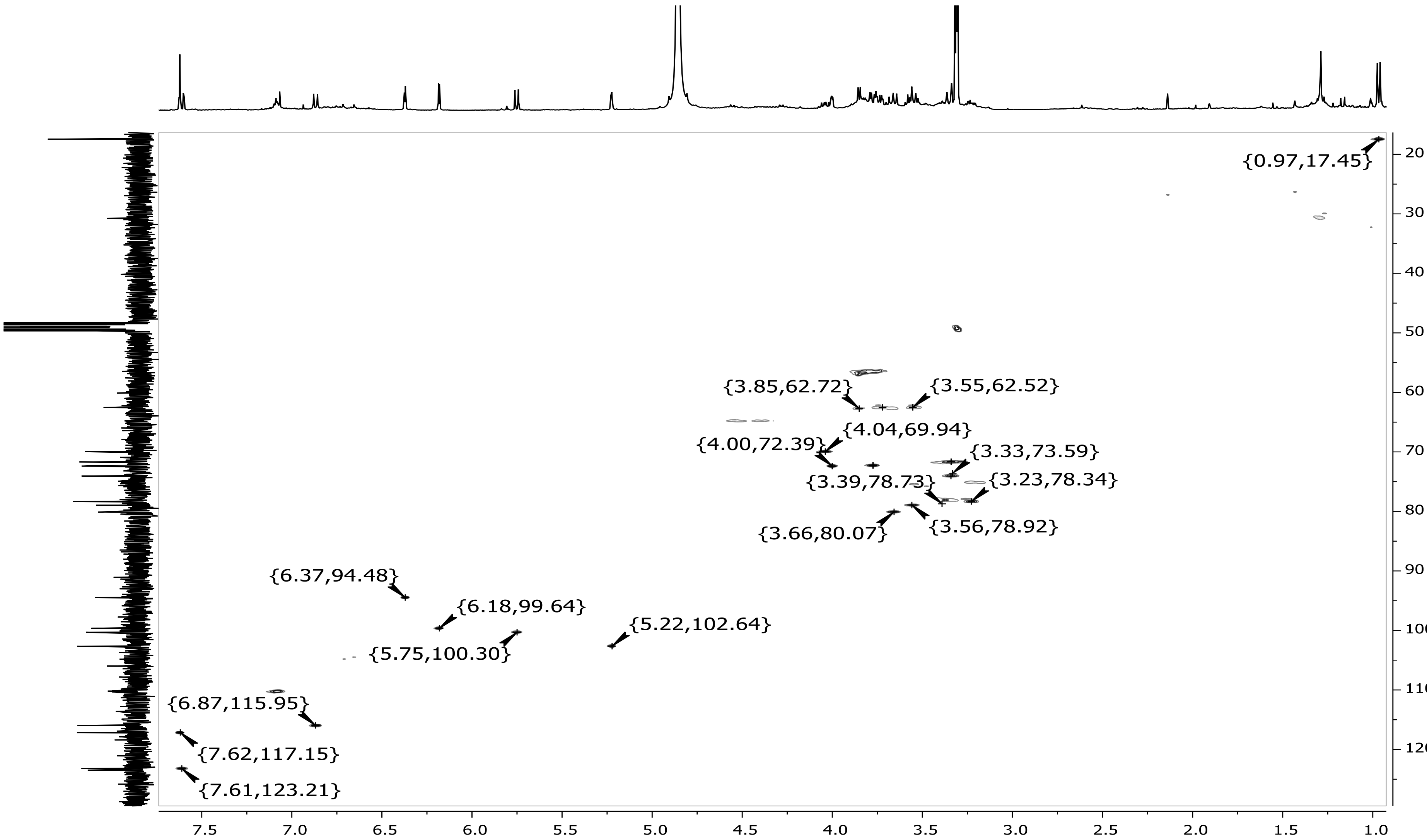

Figure S28. HSQC NMR spectrum of compound LR 24 – 61 or hecpatrin in methanol – d_4.

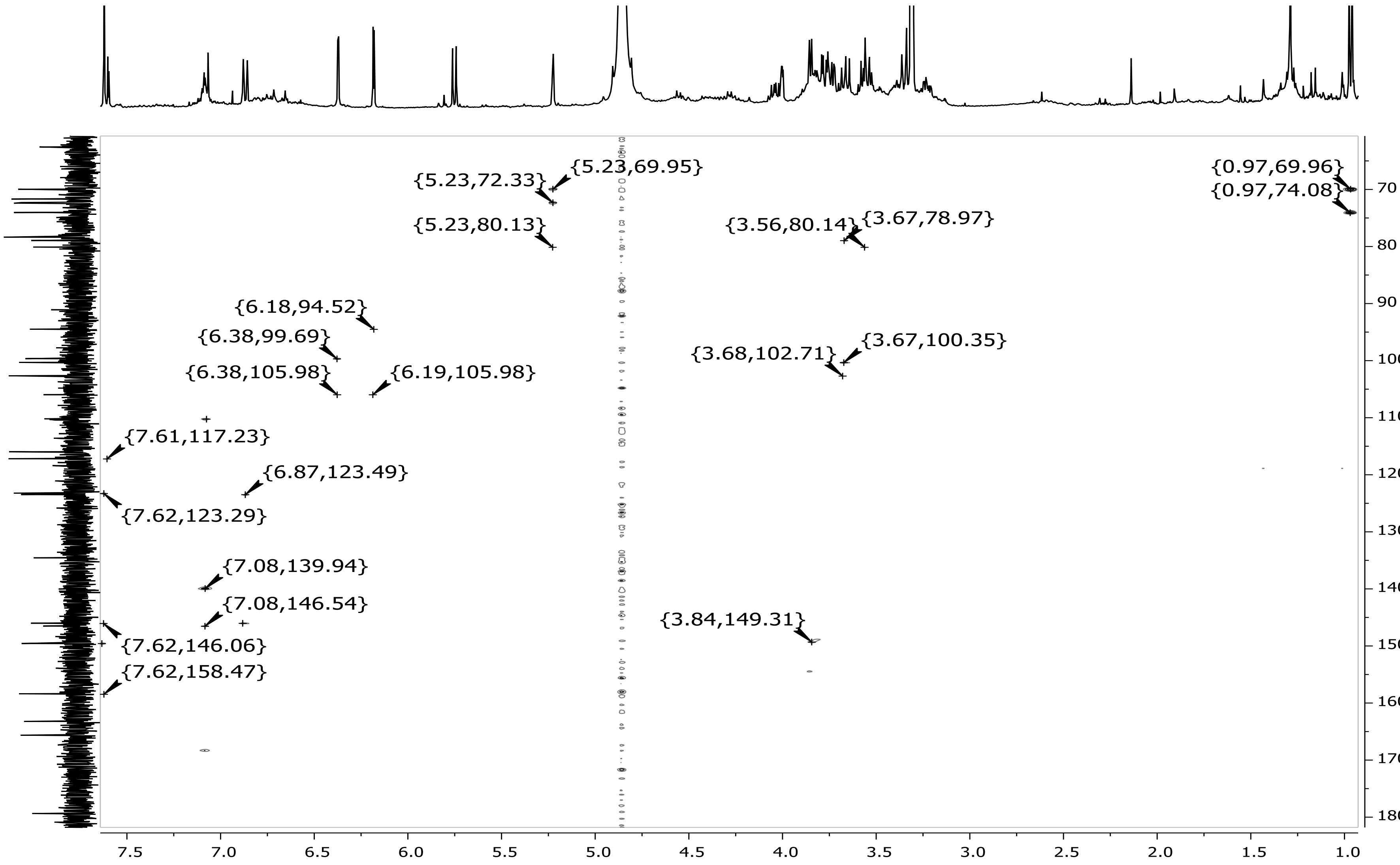

Figure S29. HMBC NMR spectrum of compound LR 24 – 61 or hecpatrin in methanol – d_4.

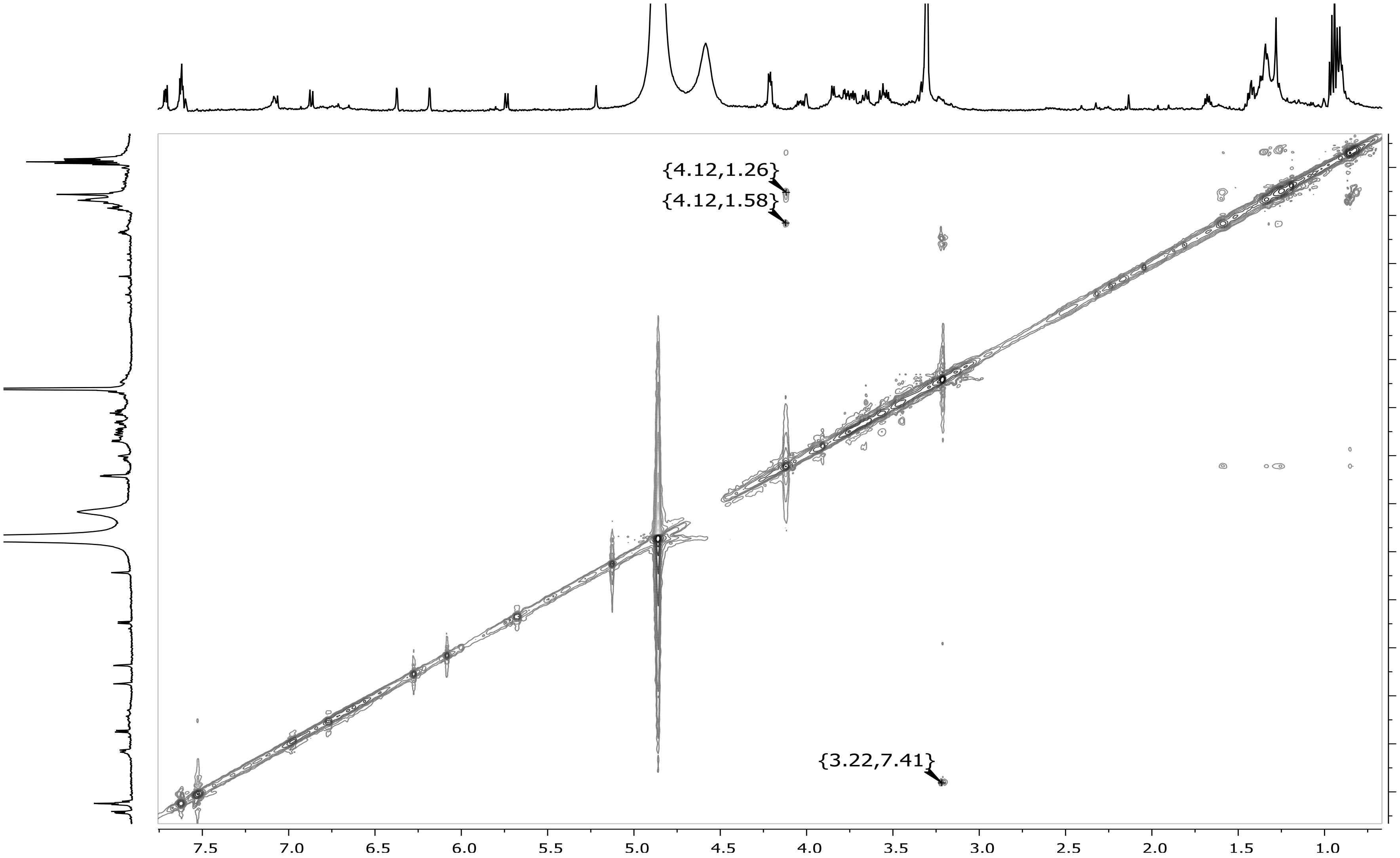

Figure S30. NOESY NMR spectrum of compound LR 24 – 61 or hecpatrin in methanol – d_4.

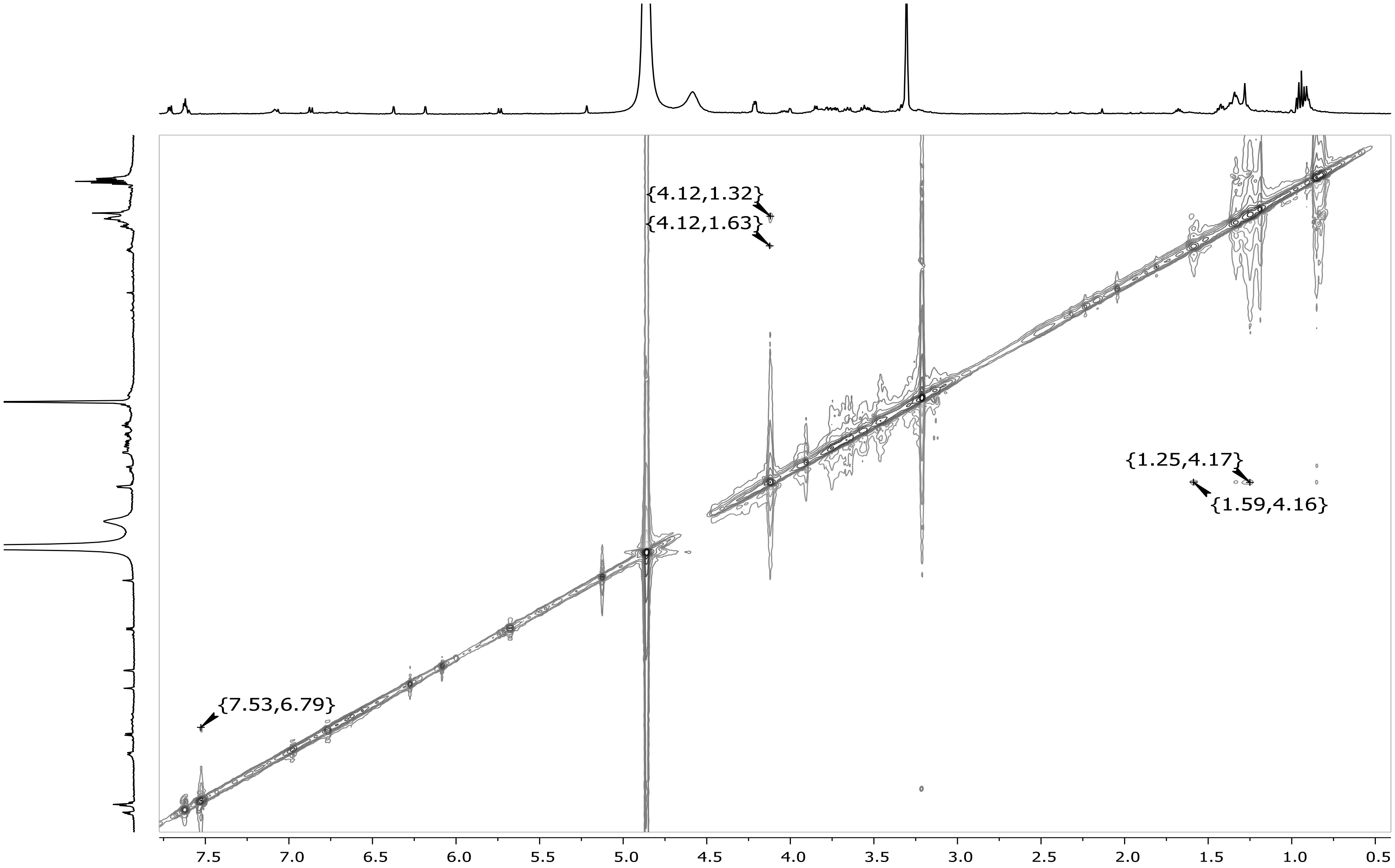

Figure S31. ROESY NMR spectrum of compound LR 24 – 61 or hecpatrin in methanol – d_4.

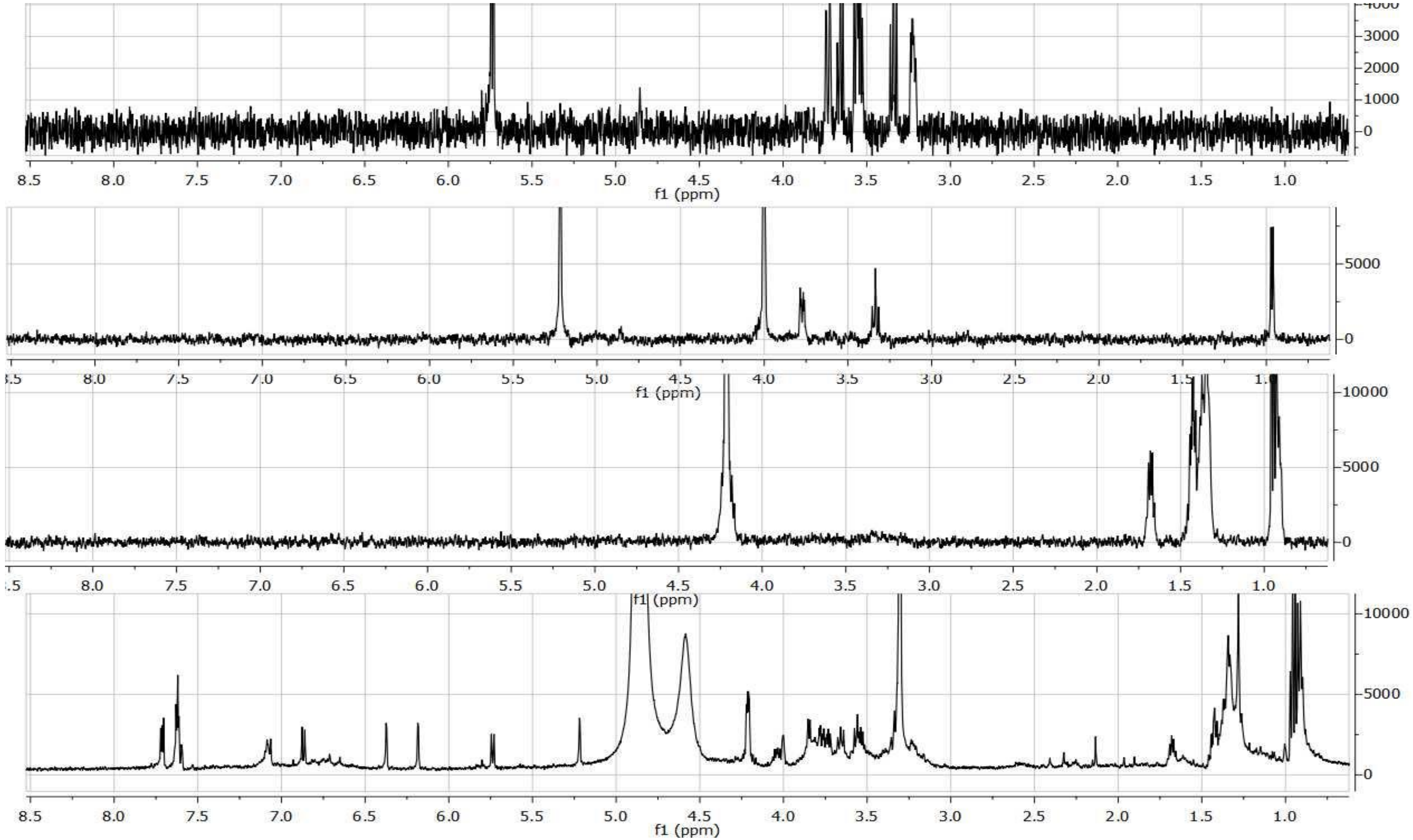

Figure S32. TOCSY NMR spectrum of compound LR 24 – 61 or hecpatrin in methanol – *d*$_4$.

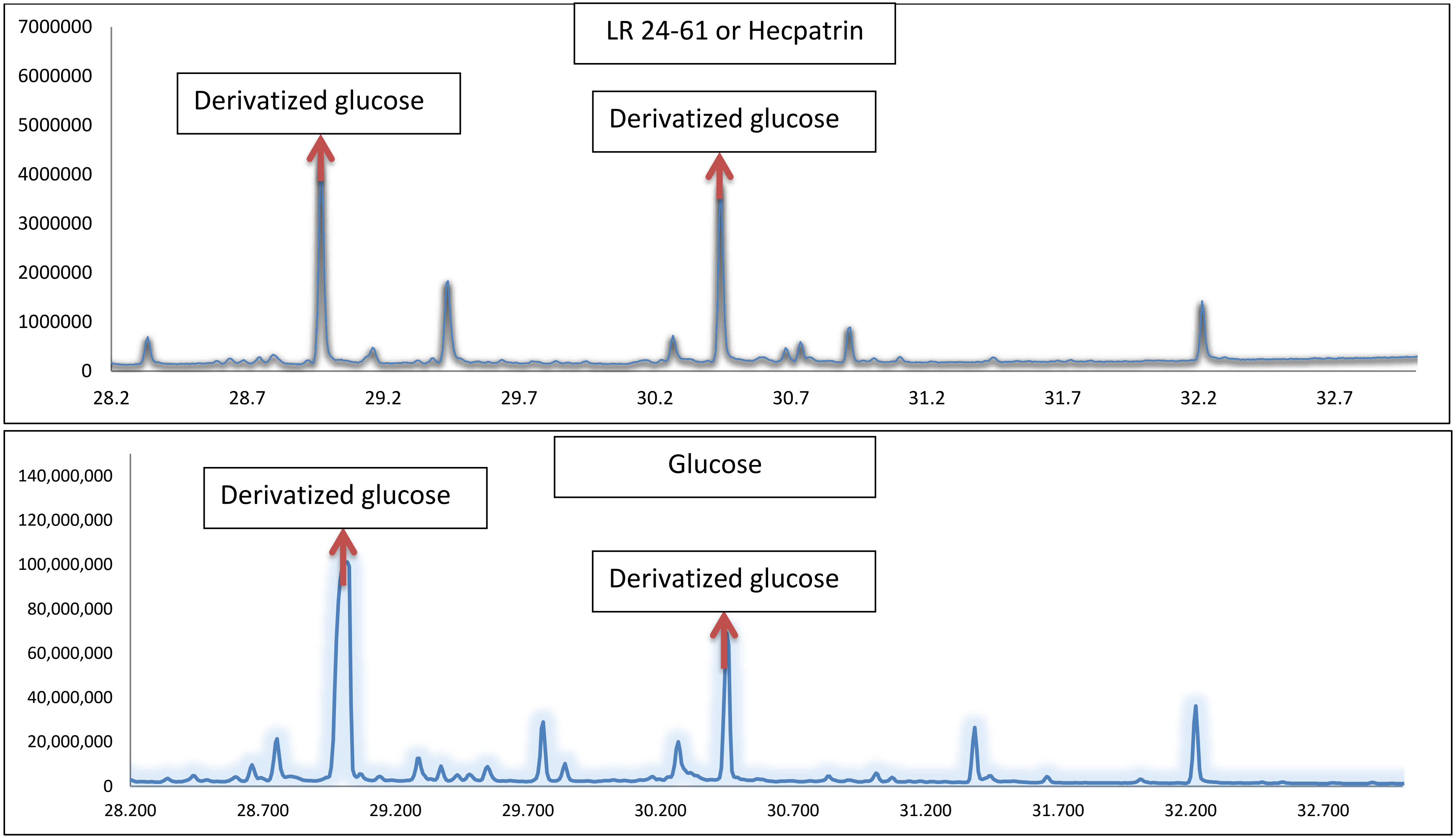

Figure S33. Cromatogram of compound LR 24 – 61 or hecpatrin in methanol – d_4.

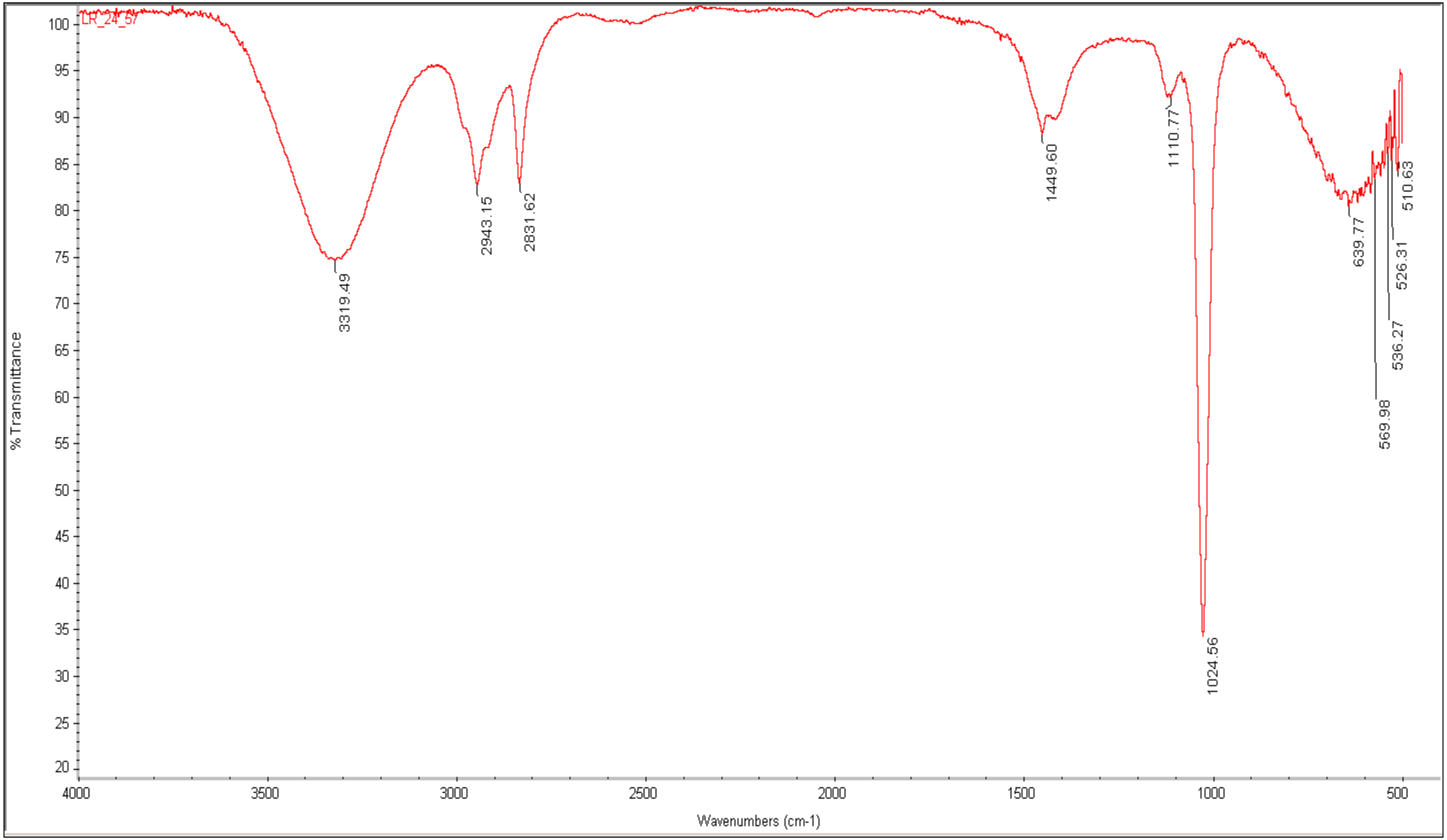

Figure S34. IR spectrum of compound LR 24-57 or gaiadendrin.

Mass Spectrum Deconvolution Report

Analysis Info

Analysis Name	D:\Data\bruker enero18\Metodo 2018\PAULO CEDENO\LR_24_57_MS.d	Acquisition Date	4/8/2019 1:03:44 PM
Method	paulo.m	Operator	BDAL@DE
Sample Name	LR_24_57_MS	Instrument	amaZon speed
Comment			

Acquisition Parameter

Ion Source Type	ESI	Ion Polarity	Positive	Alternating Ion Polarity	off
Mass Range Mode	UltraScan	Scan Begin	100 m/z	Scan End	1000 m/z
Accumulation Time	200000 µs	RF Level	63 %	Trap Drive	54.1
SPS Target Mass	400 m/z	Averages	5 Spectra	n/a	n/a

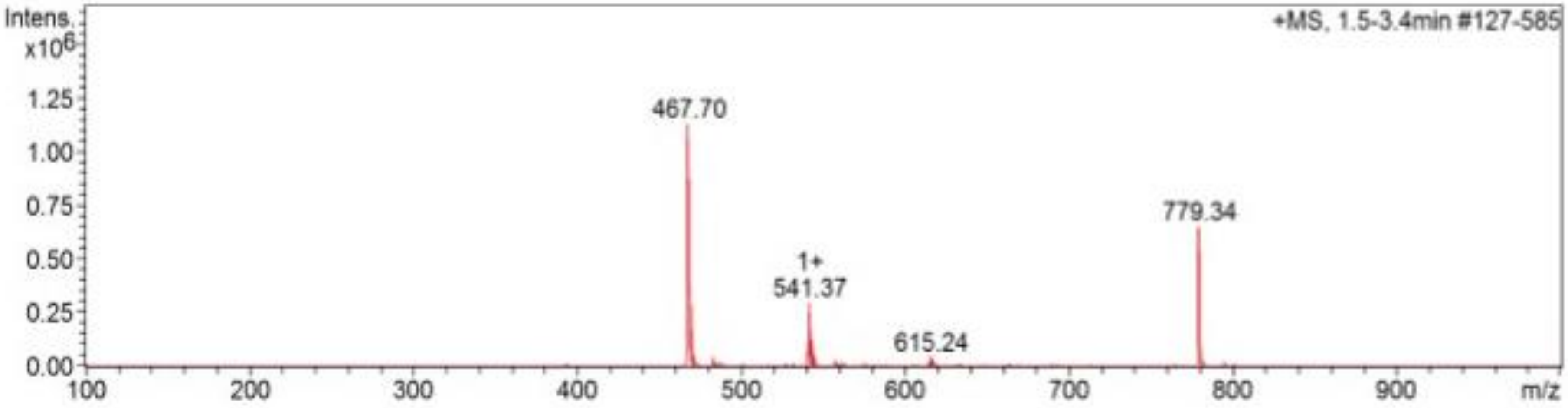

Figure S35. MS spectrum of compound LR 24 – 57 or gaiadendrin

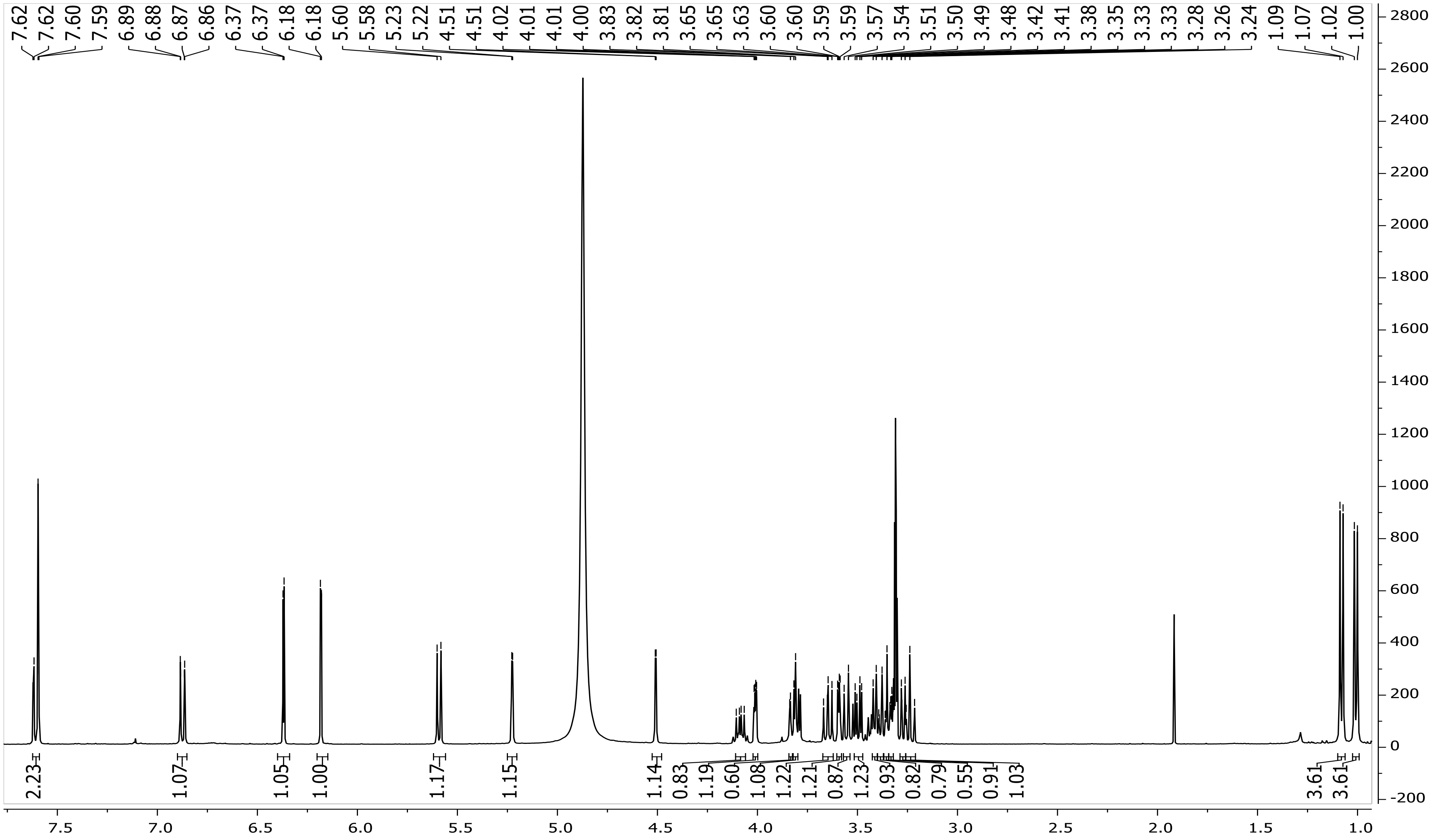

Figure S36. ^{1}H NMR spectrum of compound LR 24 – 57 or gaiadendrin in methanol – d_4.

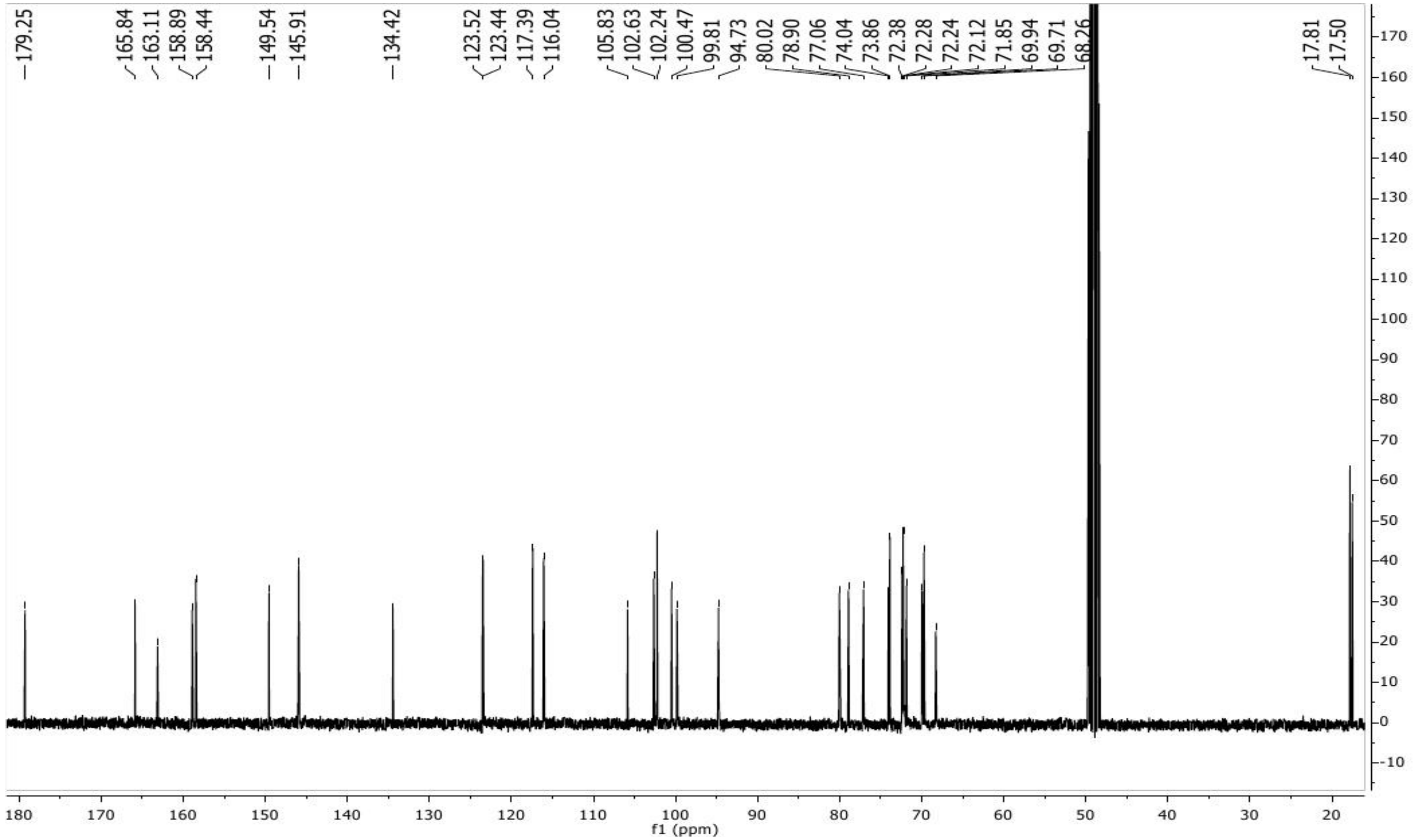

Figure S37. ^{13}C NMR spectrum of compound LR 24 – 57 or gaiadendrin in methanol – d_4

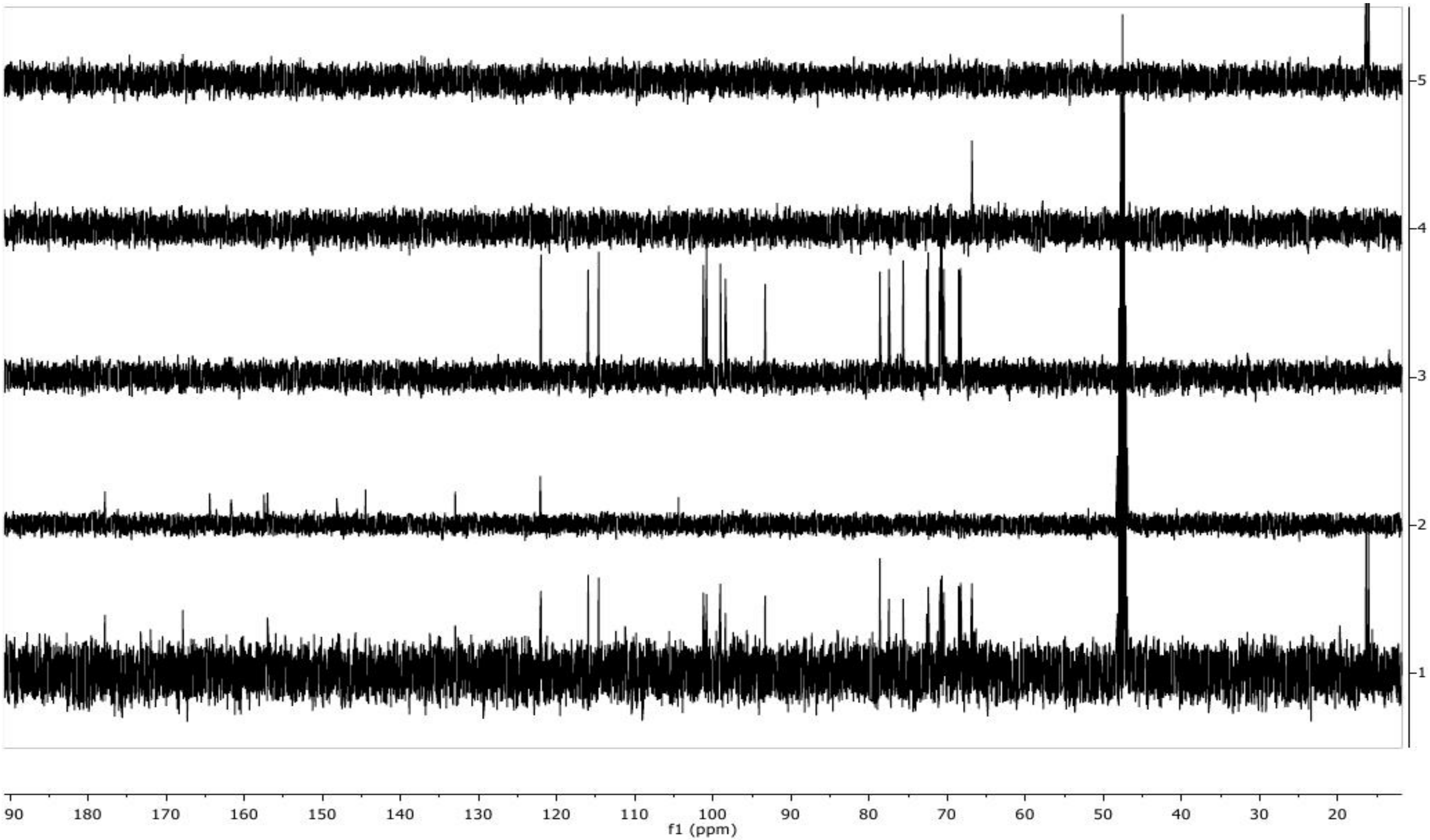

Figure S38. DEPT NMR spectrum of compound LR 24 – 57 or gaiadendrin in methanol – *d*$_4$

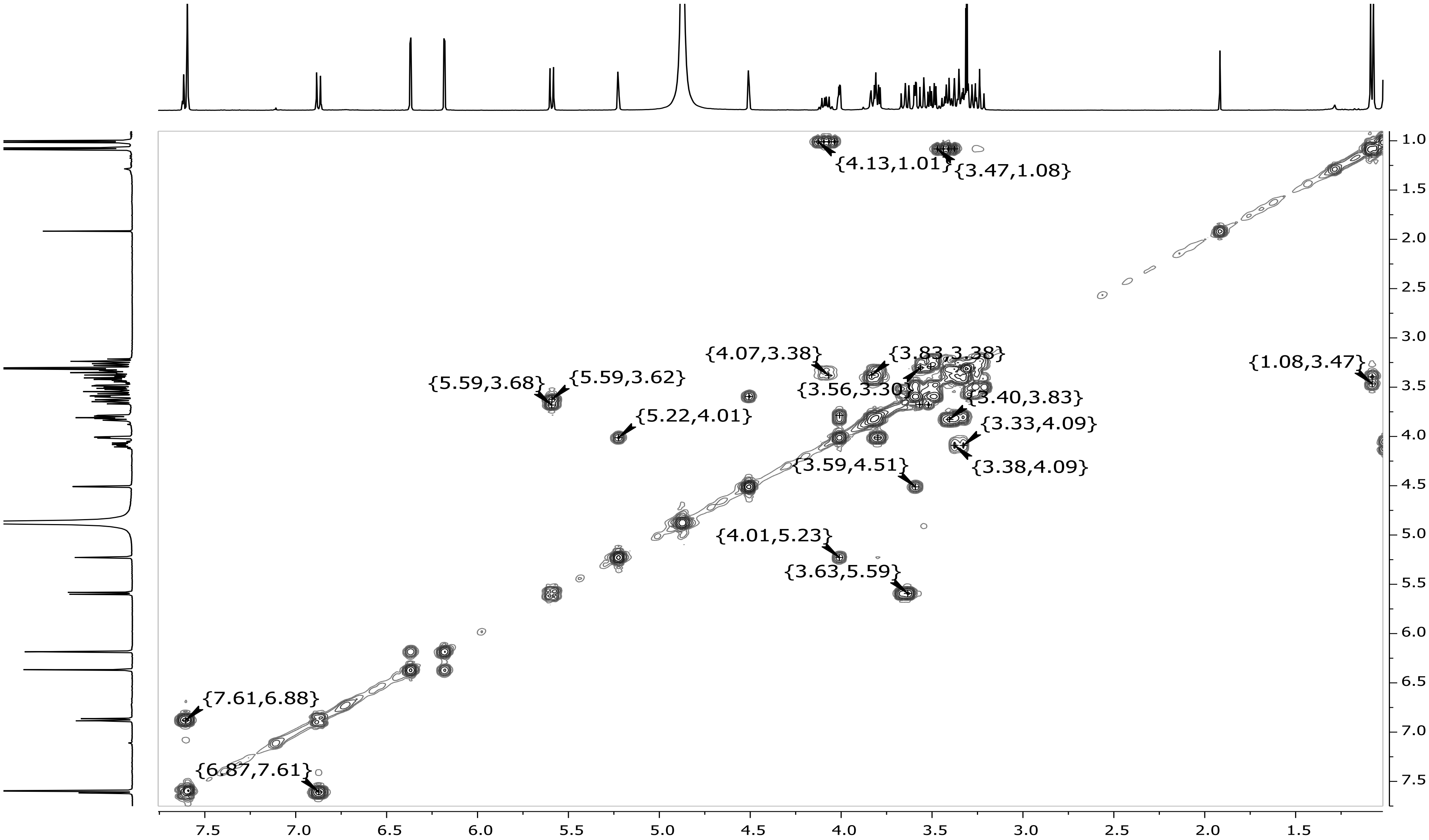

Figure S39. COSY NMR spectrum of compound LR 24 – 57 or gaiadendrin in methanol – d_4

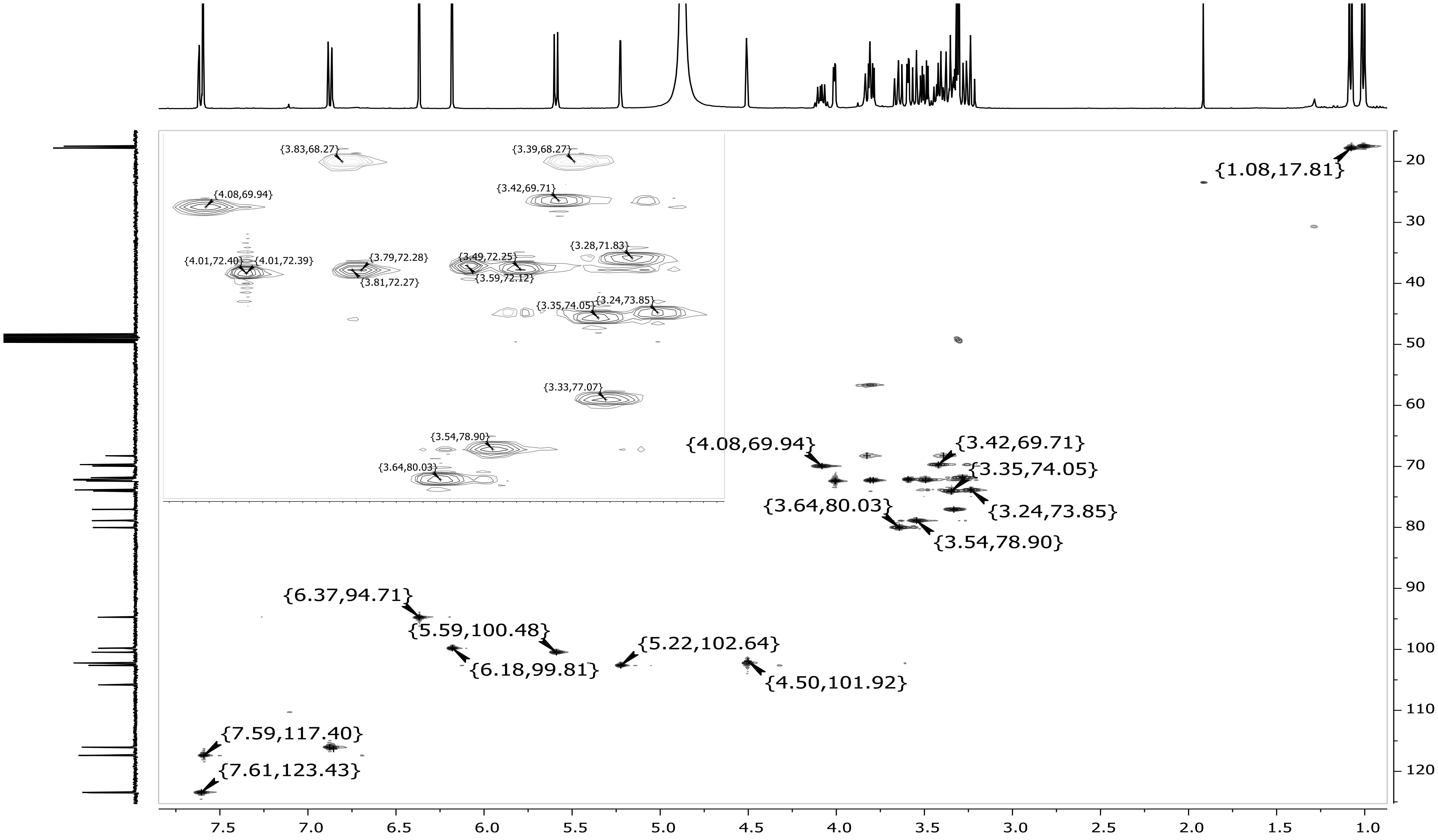

Figure S40. HSQC NMR spectrum of compound LR 24 – 57 or gaiadendrin in methanol – d_4

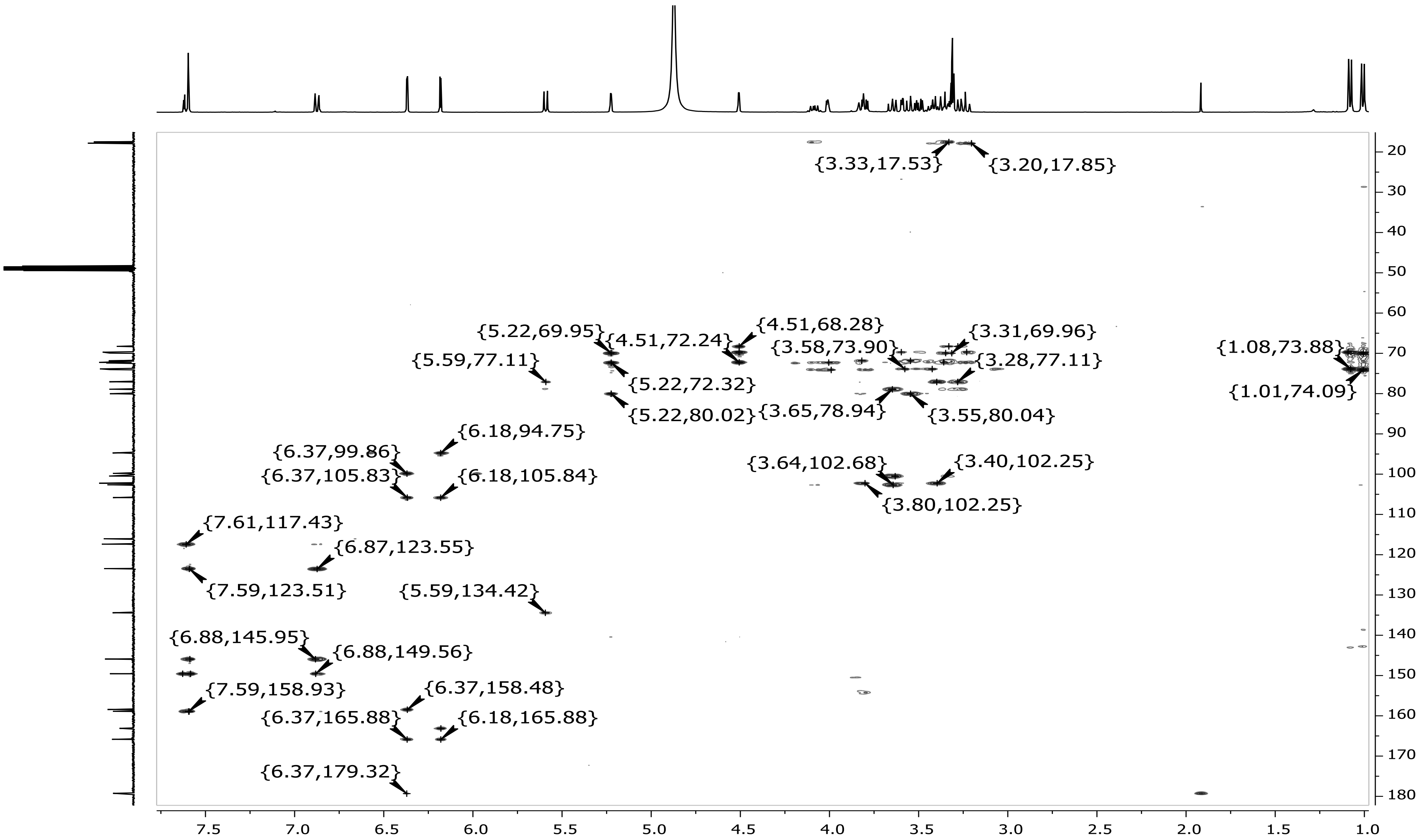

Figure S41. HMBC spectrum of compound LR 24 – 57 or gaiadendrin in methanol – d_4.

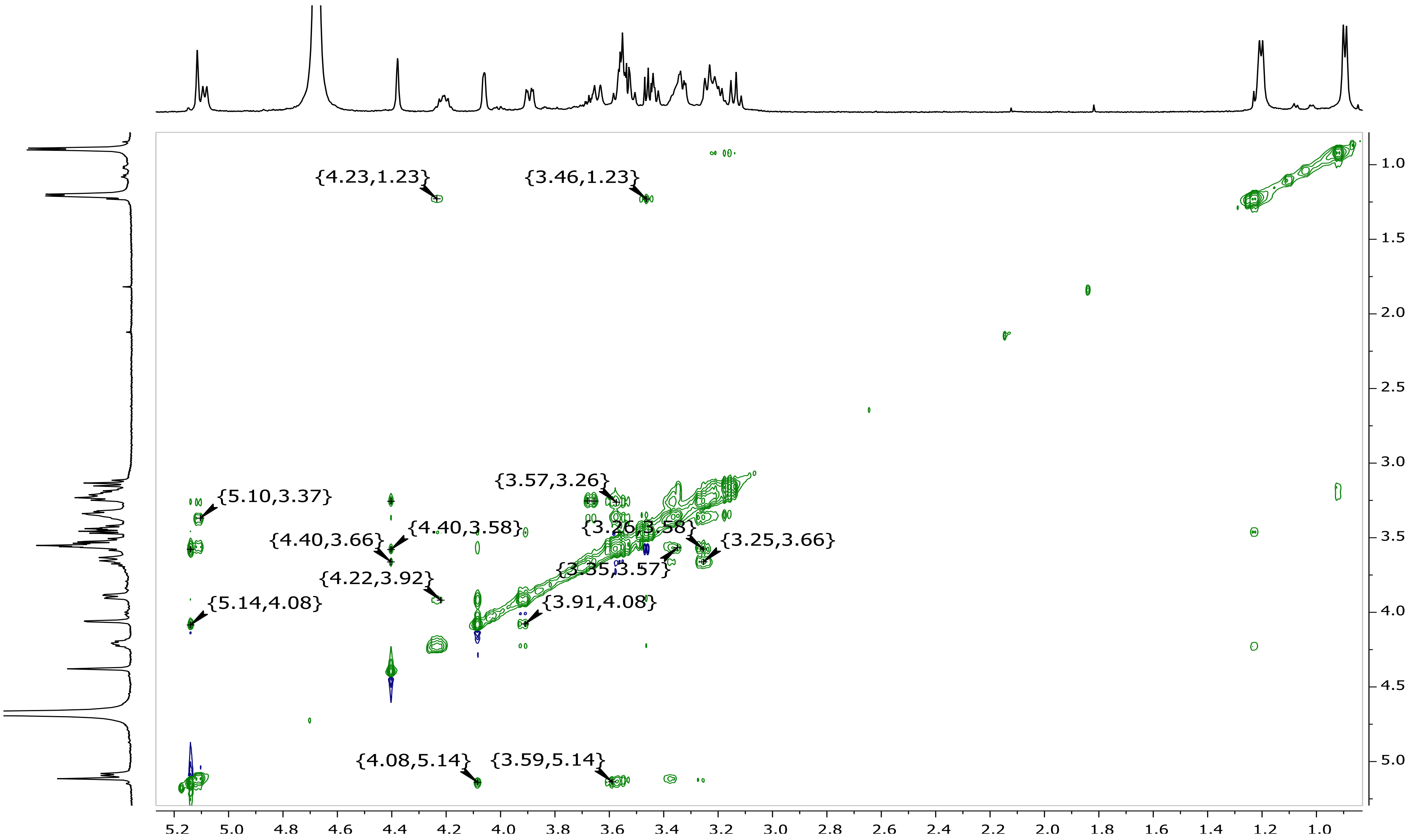

Figure S42. NOESY spectrum of compound LR 24 – 57 or gaiadendrin in deuterium oxide.

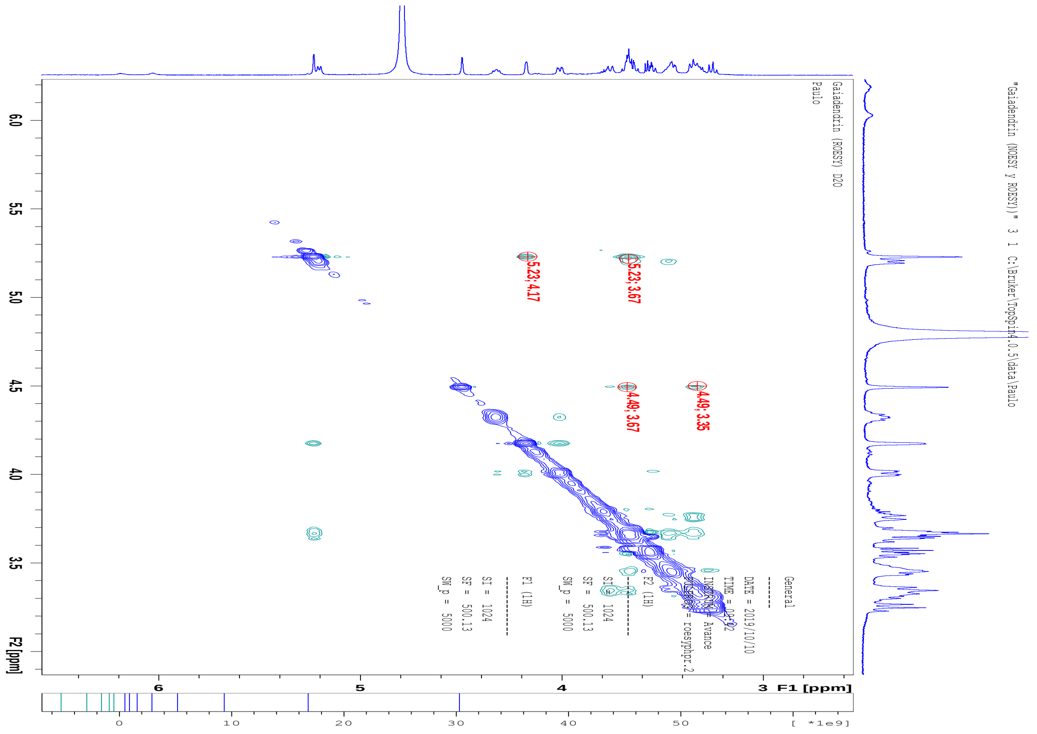

Figure S43. ROESY spectrum of compound LR 24 – 57 or gaiadendrin in deuterium oxide.

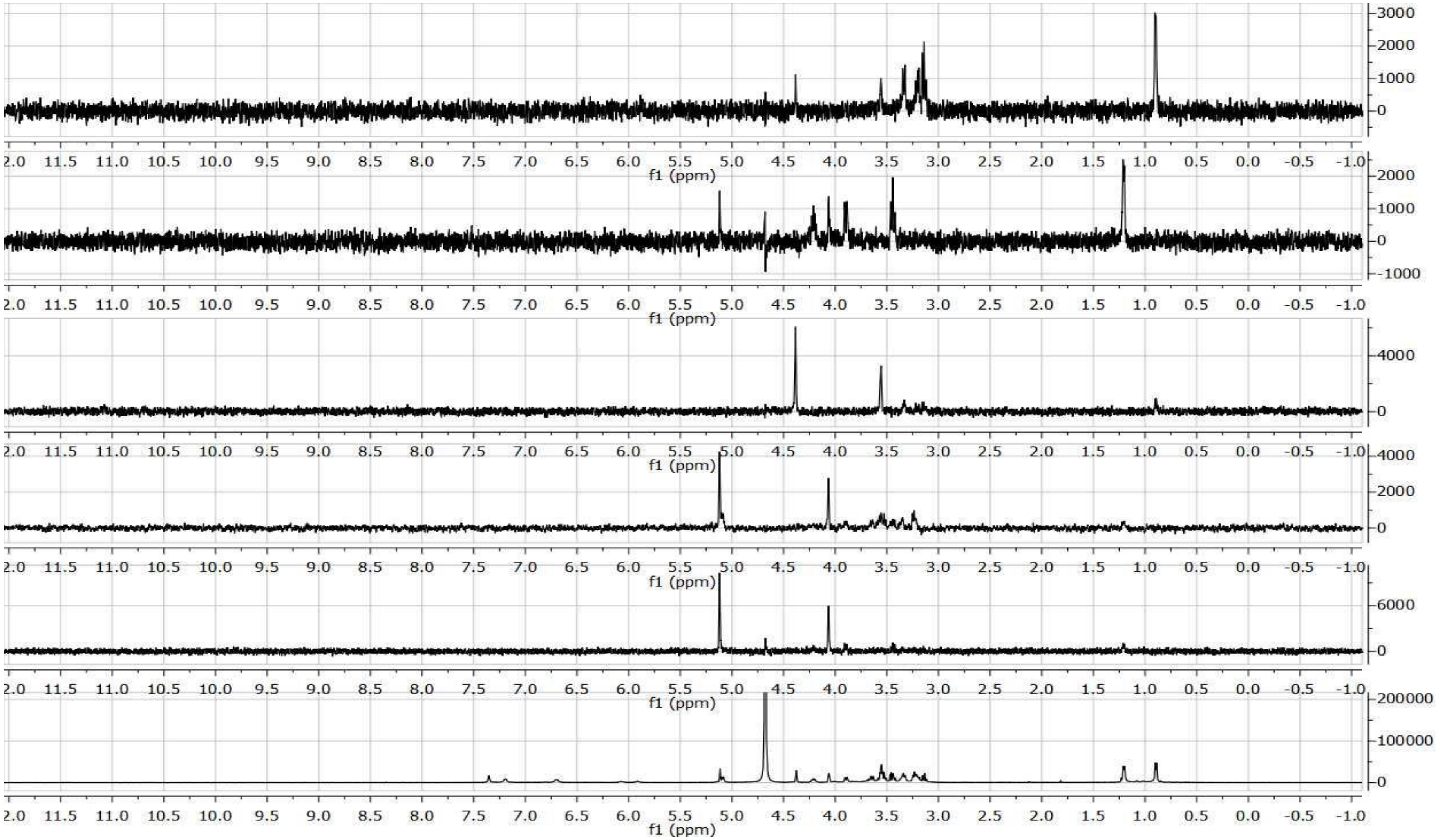

Figure S44. TOCSY spectrum of compound LR 24 – 57 or gaiadendrin in deuterium oxide.

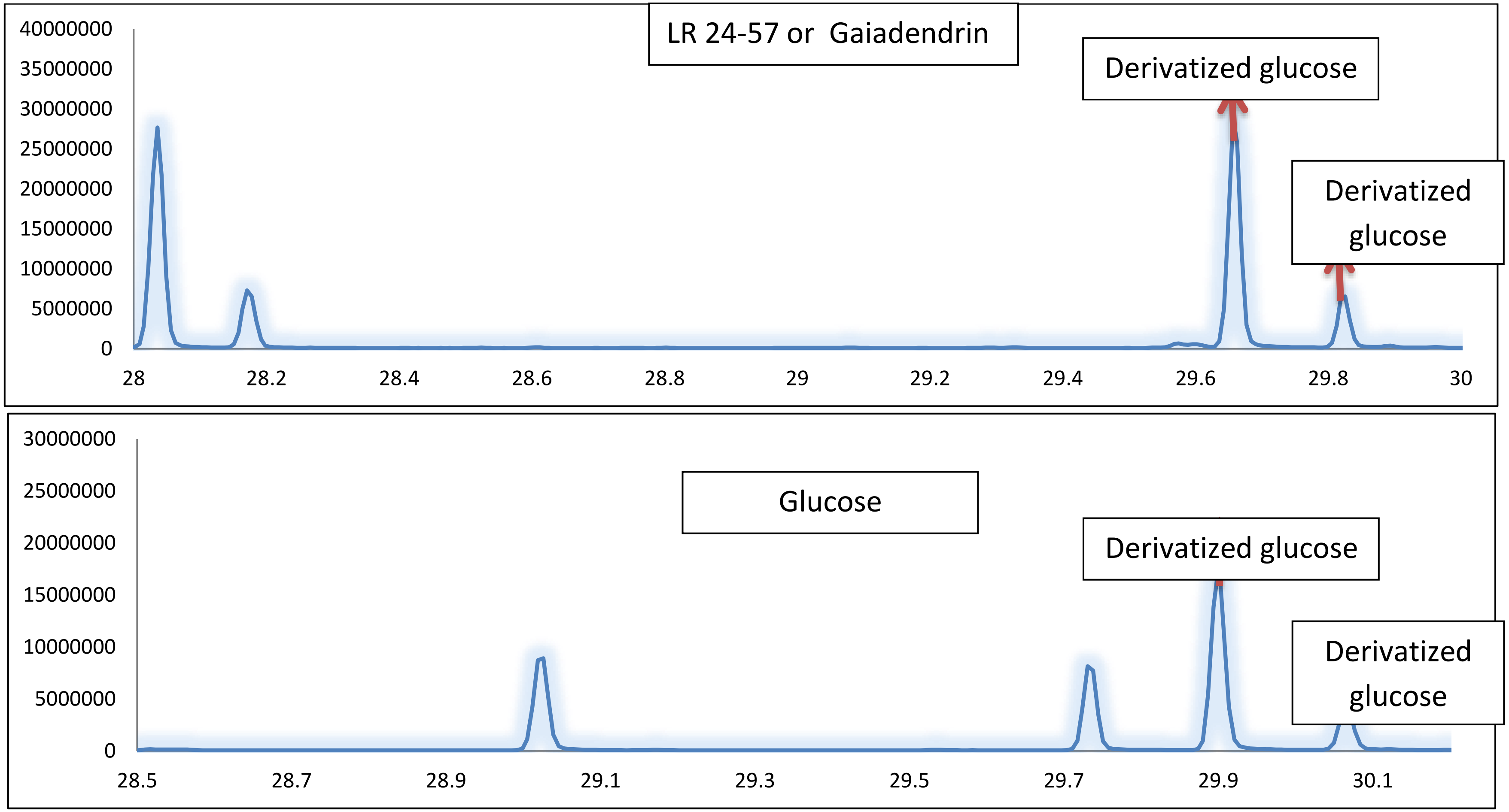

Figure S45. Cromatogram of compound LR 24 – 57 or gaiadendrin.

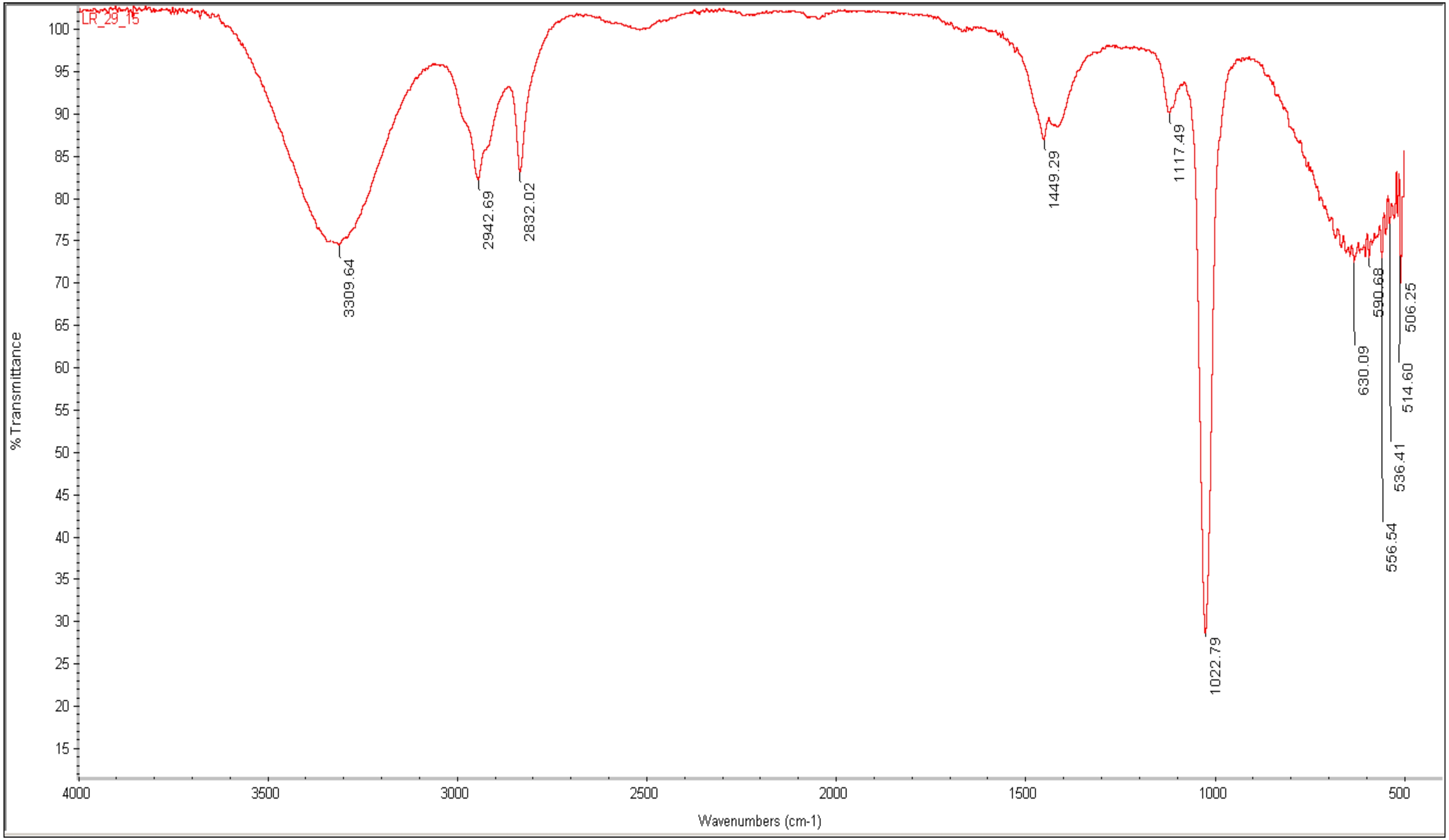

Figure S46. IR spectrum of compound LR 29-15 or puchikrin.

Mass Spectrum Deconvolution Report

Analysis Info

Analysis Name	D:\Data\bruker enero18\Metodo 2018\PAULO CEDENO\LR_29_15_MS.d	Acquisition Date	4/8/2019 1:46:24 PM
Method	paulo.m	Operator	BDAL@DE
Sample Name	LR_29_15_MS	Instrument	amaZon speed
Comment			

Acquisition Parameter

Ion Source Type	ESI	Ion Polarity	Positive	Alternating Ion Polarity	off
Mass Range Mode	UltraScan	Scan Begin	100 m/z	Scan End	1000 m/z
Accumulation Time	2782 µs	RF Level	63 %	Trap Drive	54.1
SPS Target Mass	400 m/z	Averages	5 Spectra	n/a	n/a

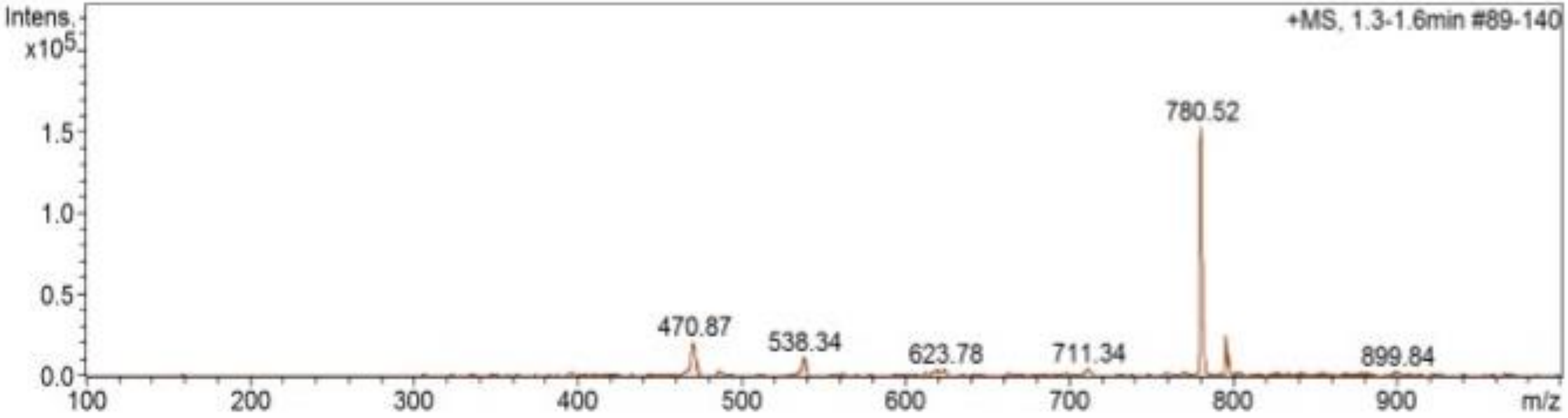

Figure S47. MS spectrum of compound LR 29 – 15 or puchikrin.

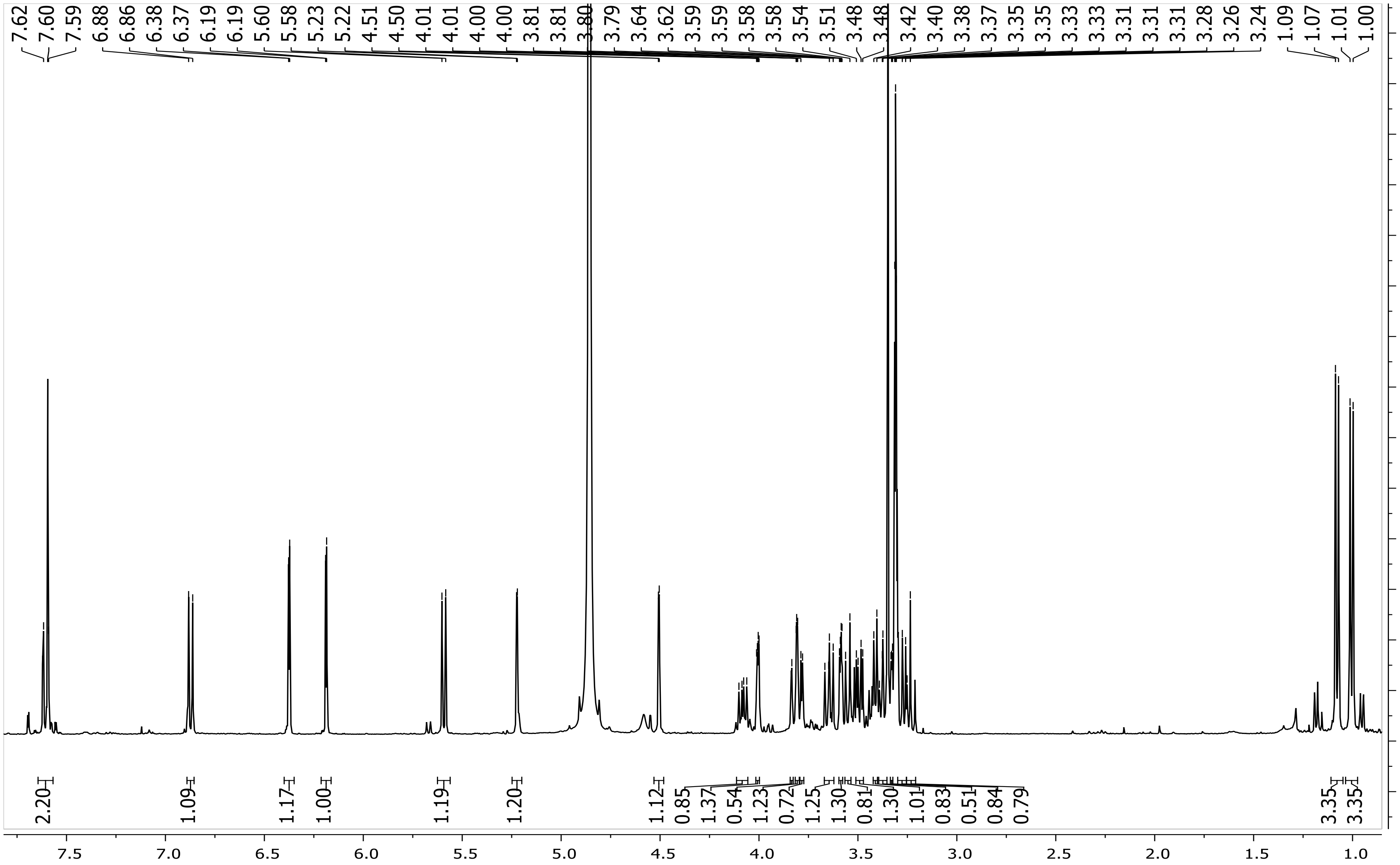

Figure S48. ^{1}H NMR spectrum of compound LR 29 – 15 or puchikrin in methanol – *d*4.

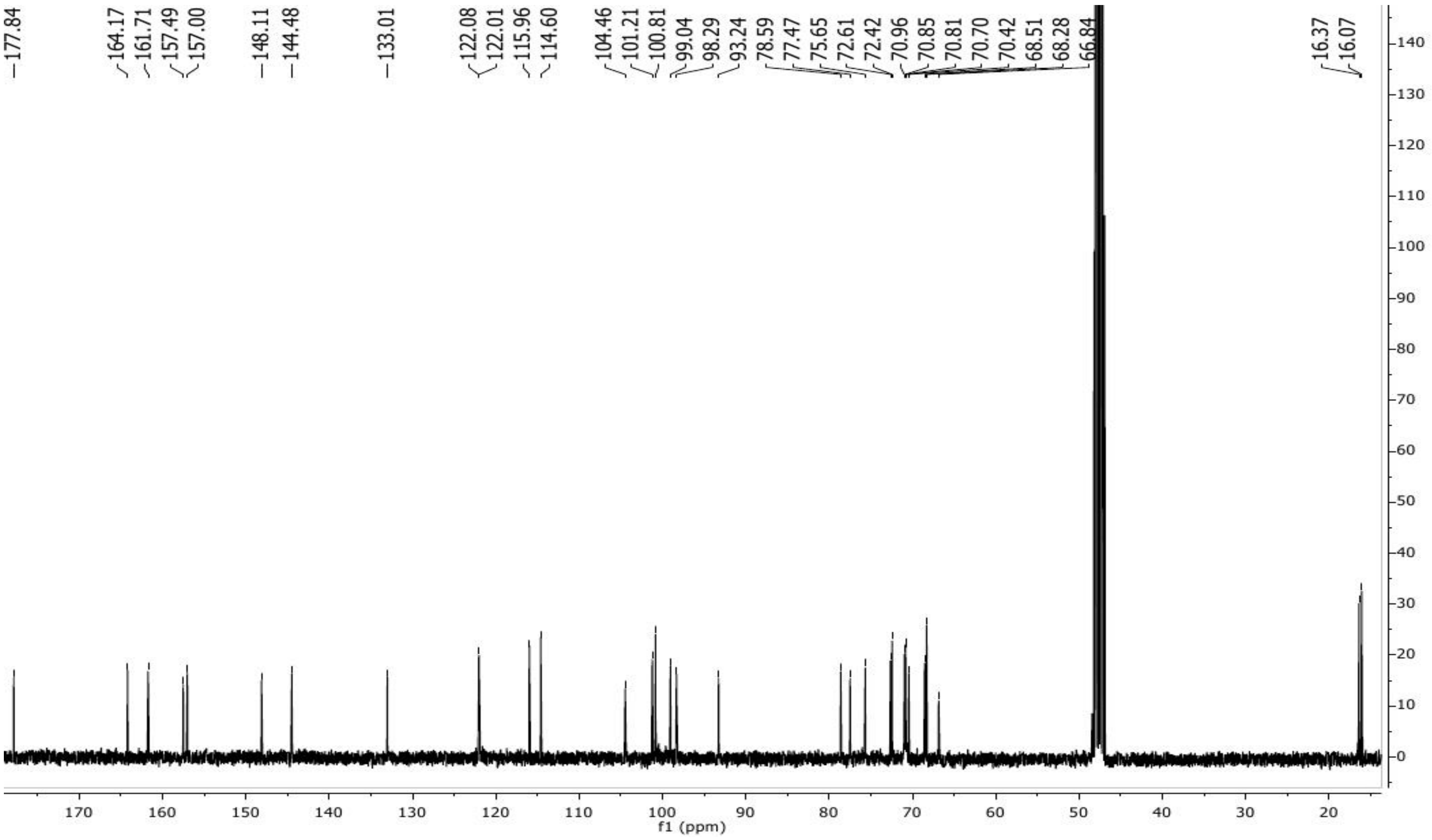

Figure S49. ^{13}C NMR spectrum of compound LR 29 – 15 or puchikrin in methanol – d_4.

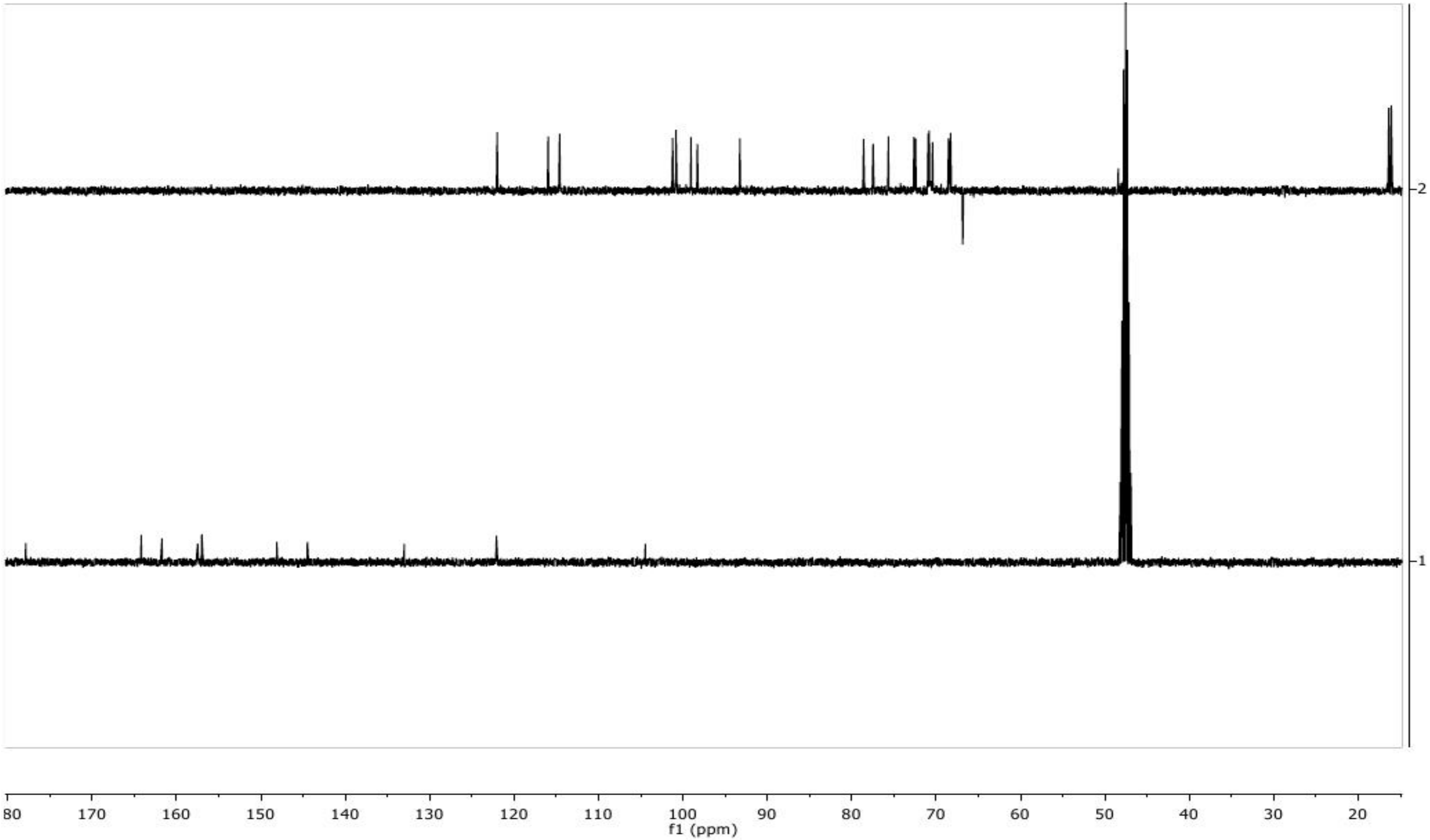

Figure S50. DEPT NMR spectrum of compound LR 29 – 15 or puchikrin in methanol – d_4.

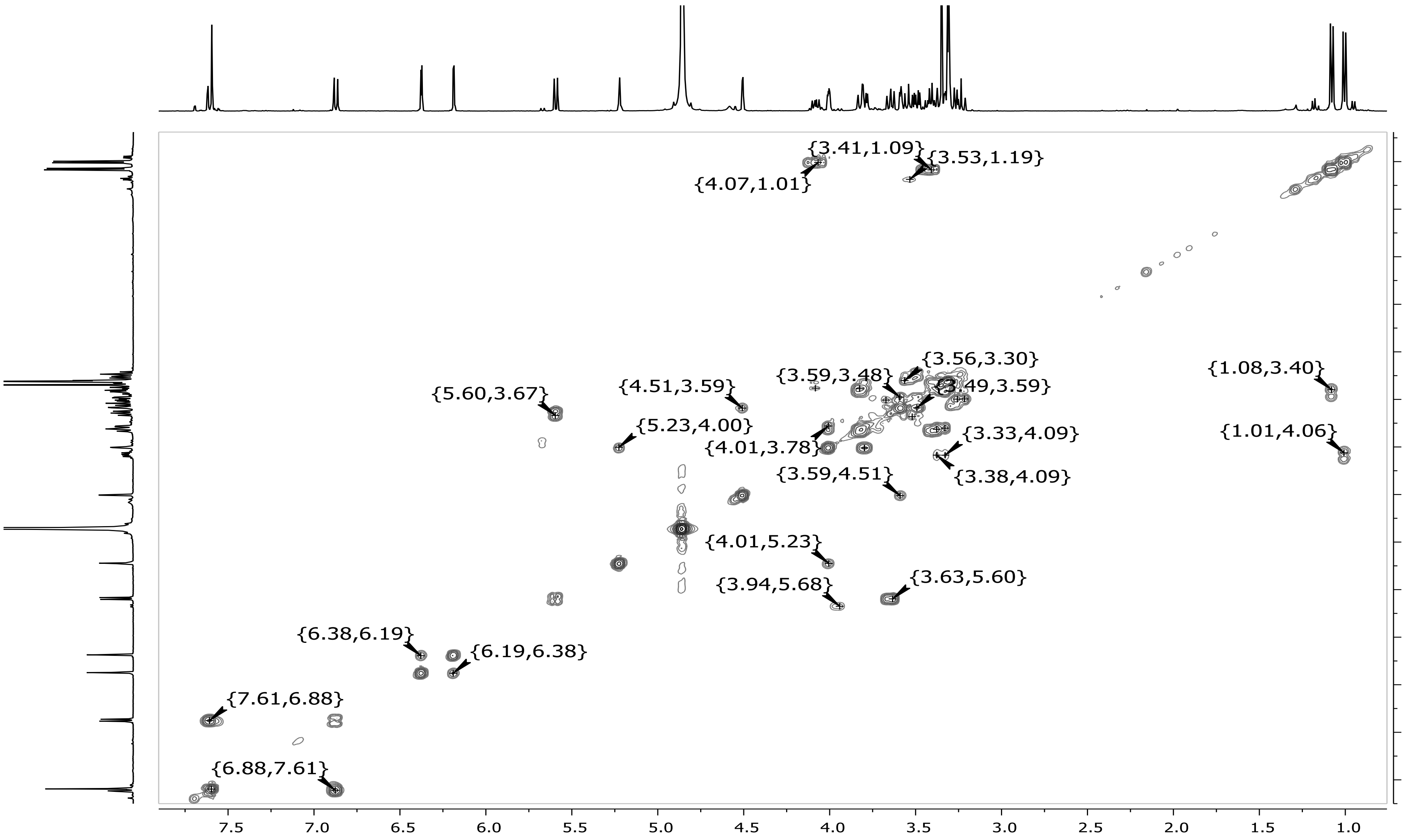

Figure S51. COSY NMR spectrum of compound LR 29 – 15 or puchikrin in methanol – d_4.

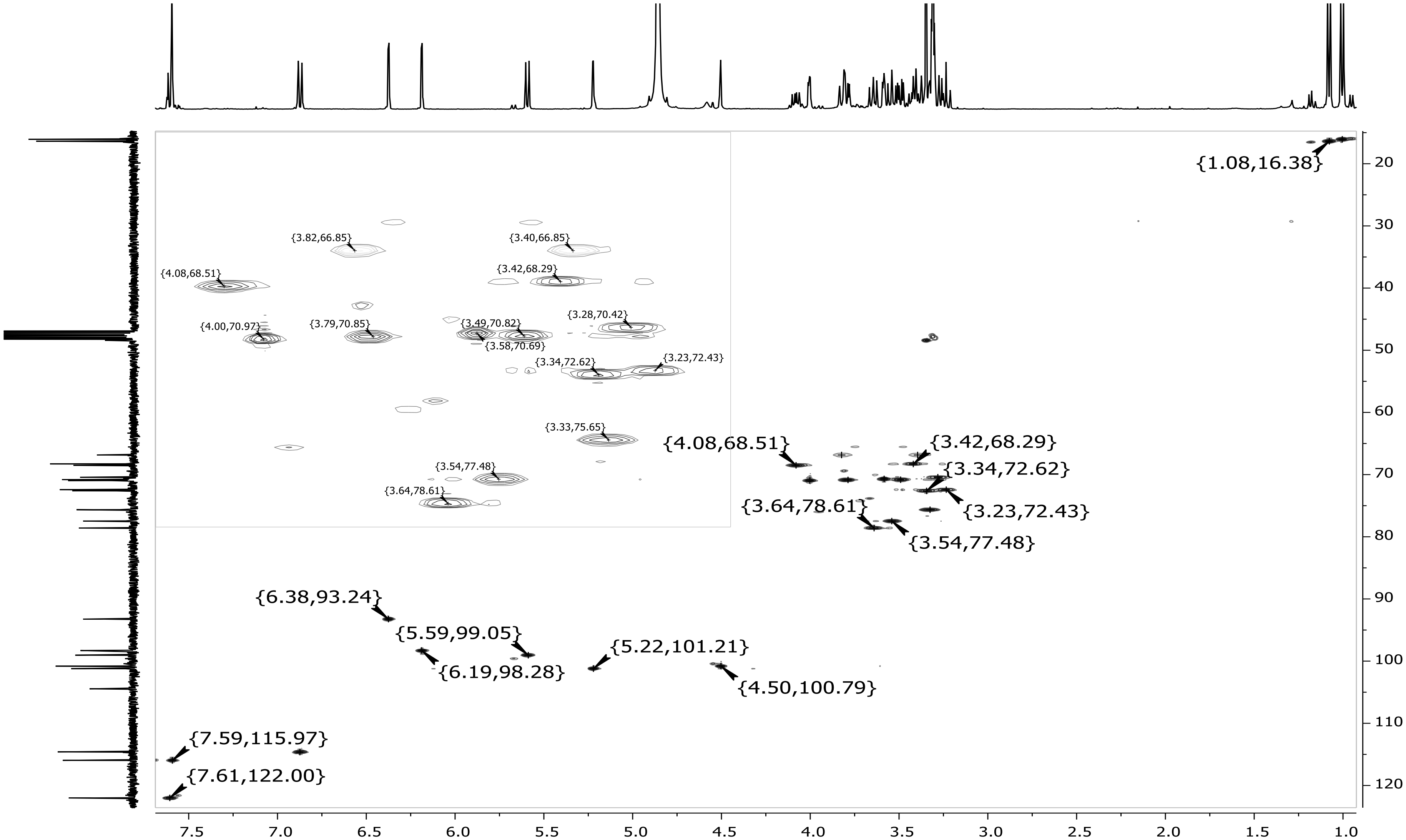

Figure S52. HSQC NMR spectrum of compound LR 29 – 15 or puchikrin in methanol – d_4.

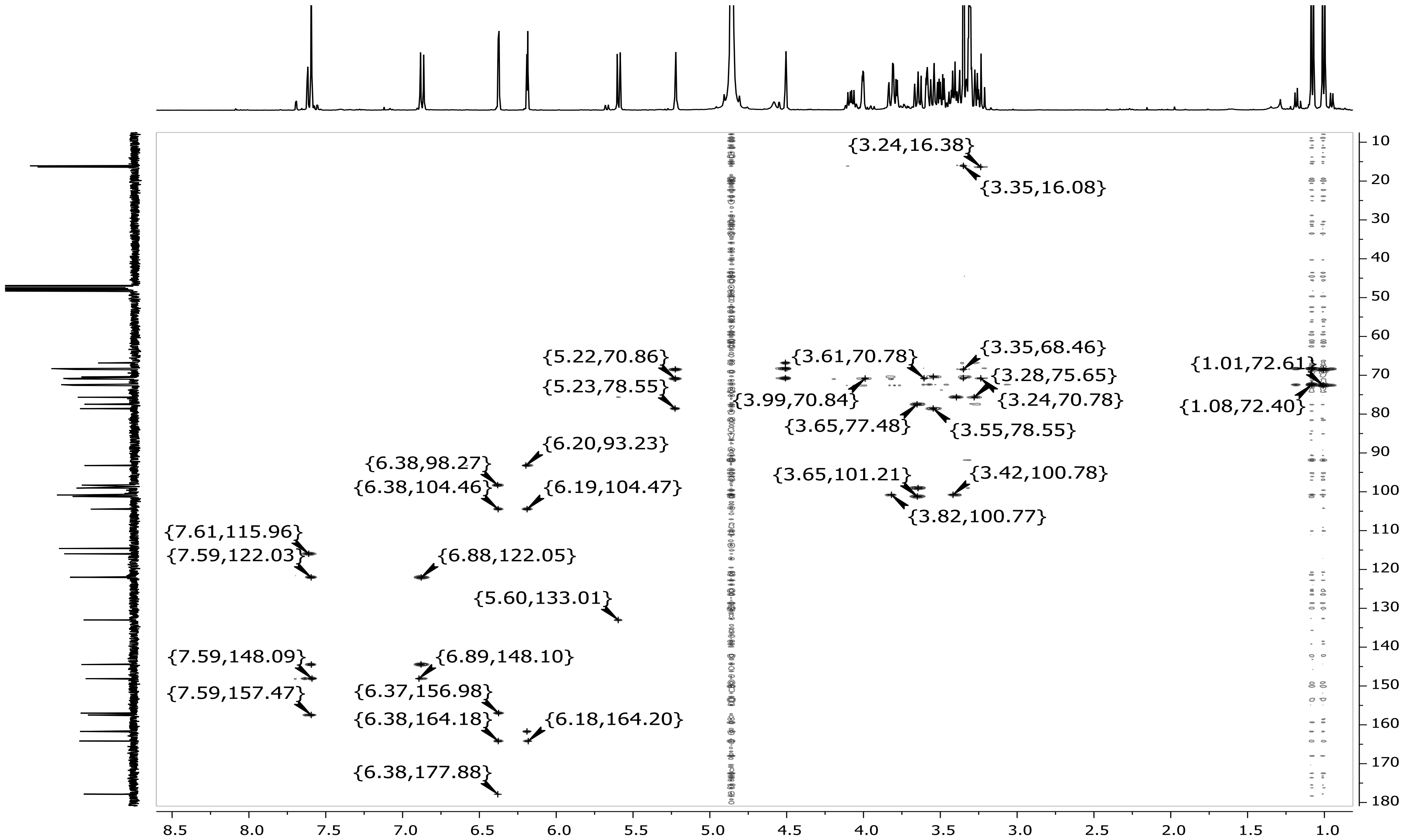

Figure S53. HMBC NMR spectrum of compound LR 29 – 15 or puchikrin in methanol – d_4.

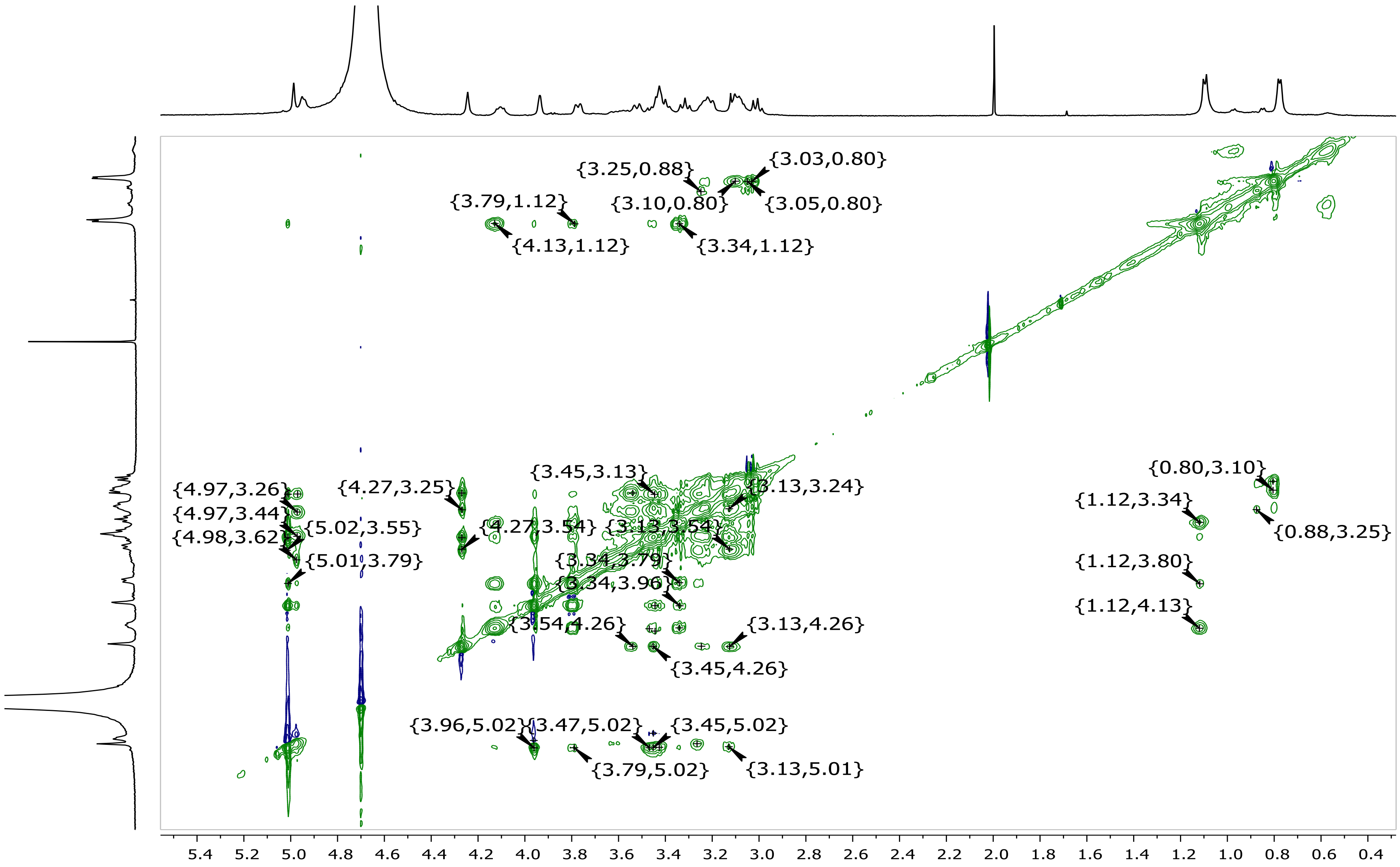

Figure S54. NOESY NMR spectrum of compound LR 29 – 15 or puchikrin in deuterium oxide.

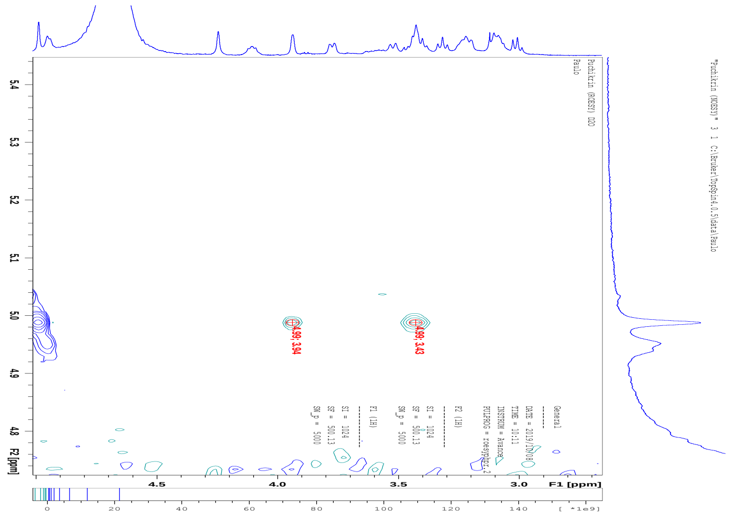

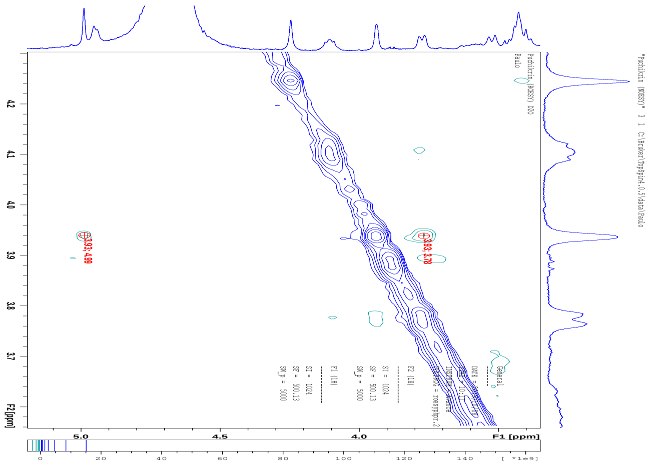

Figure S55. ROESY NMR spectrum of compound LR 29 – 15 or puchikrin in deuterium oxide.

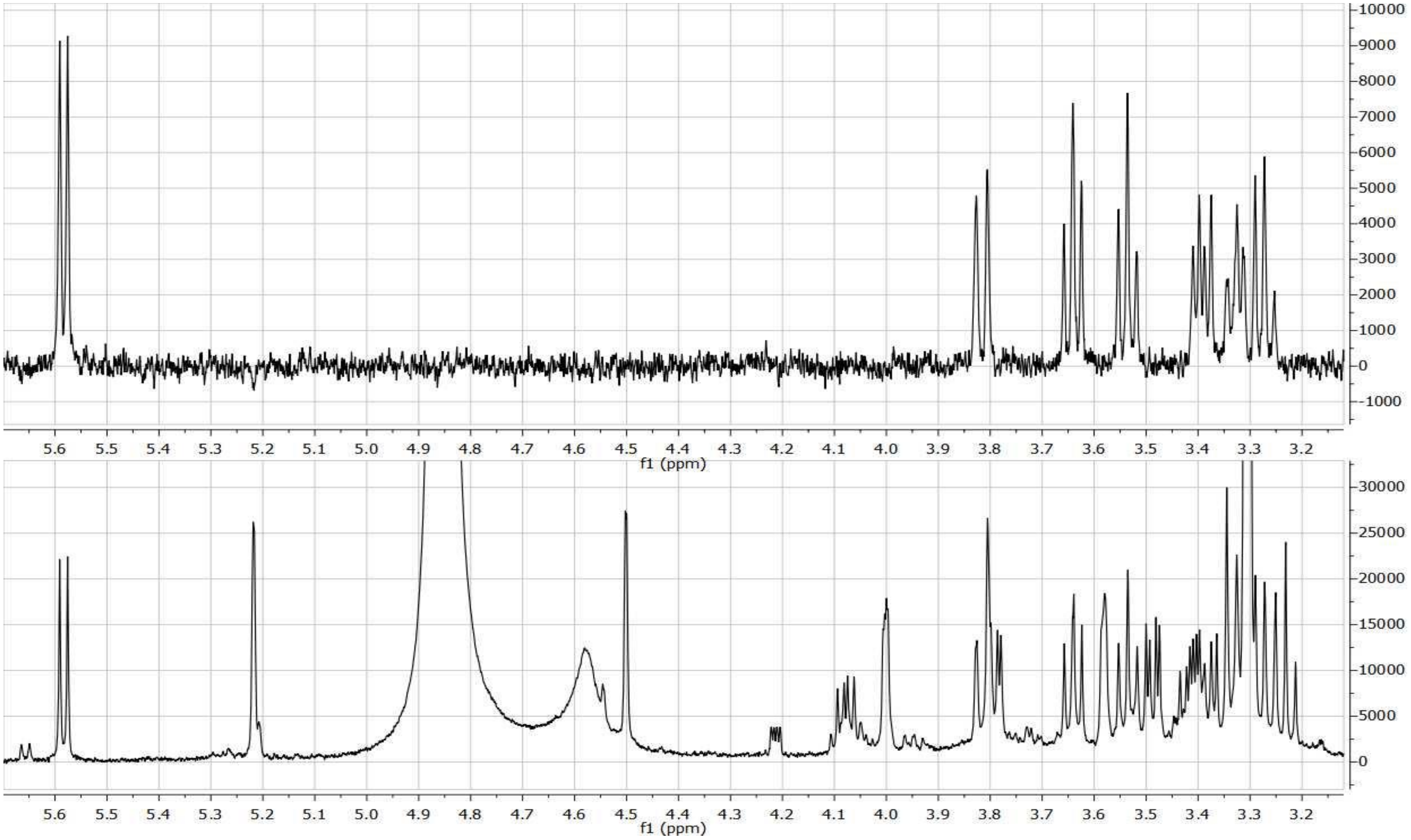

Figure S56. TOCSY NMR spectrum of compound LR 29 – 15 or puchikrin in deuterium oxide.

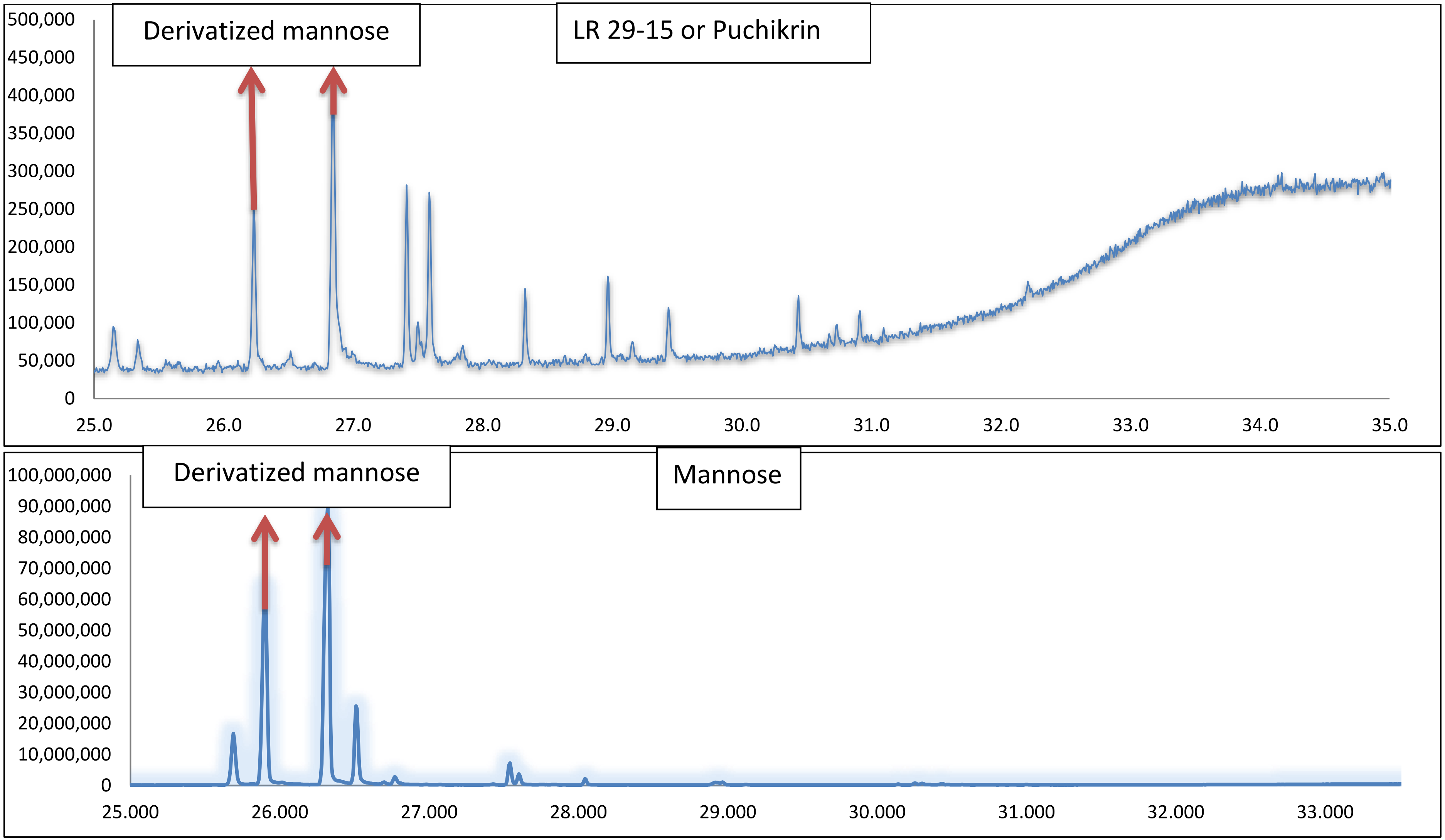

Figure S57. Cromatogram of compound LR 29 – 15 or puchikrin.

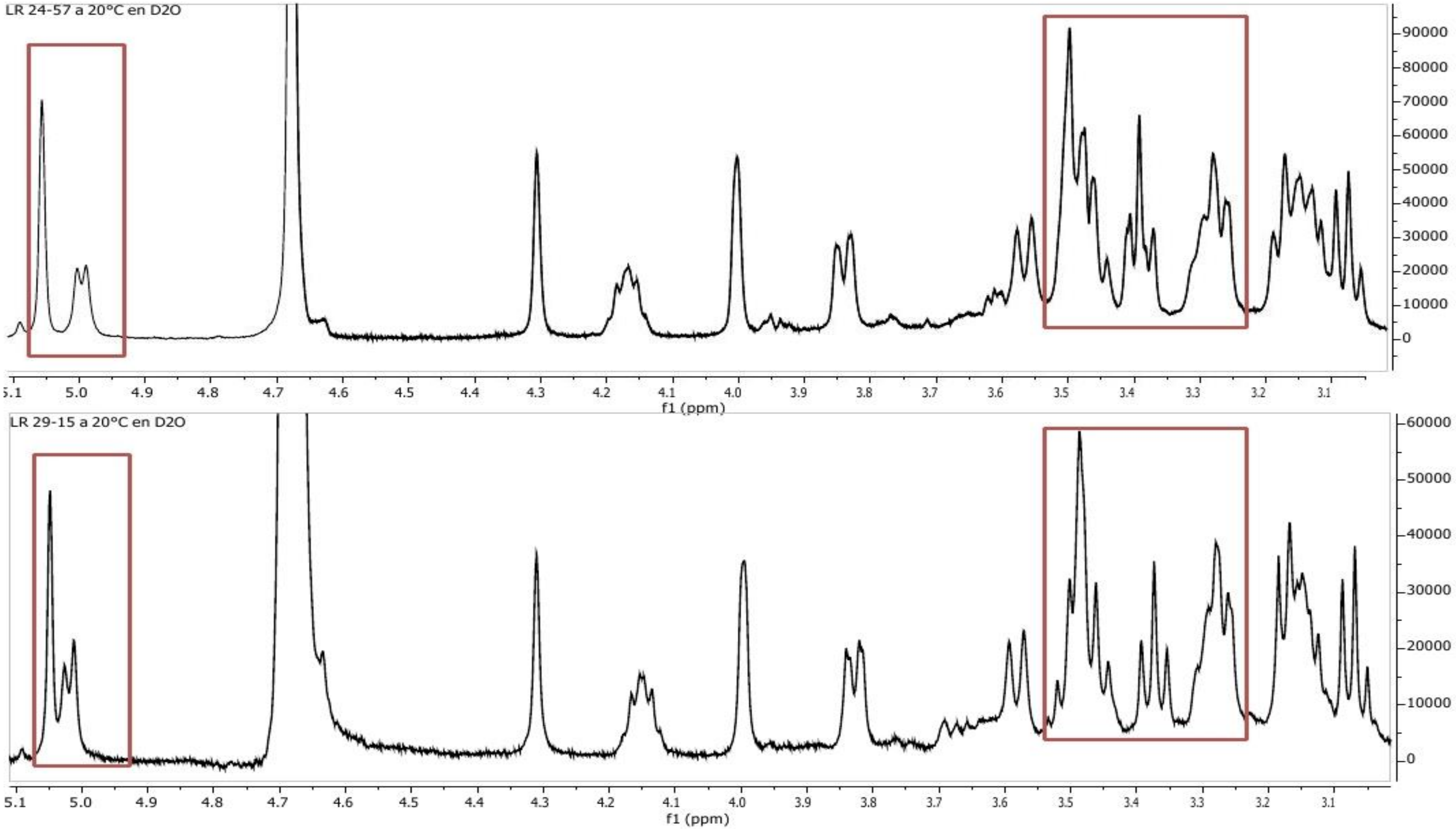

Figure S58. Comparison of ^{1}H NMR spectrum of compound LR 24 – 57 and LR 29- 15 in deuterium oxide at 20°C.

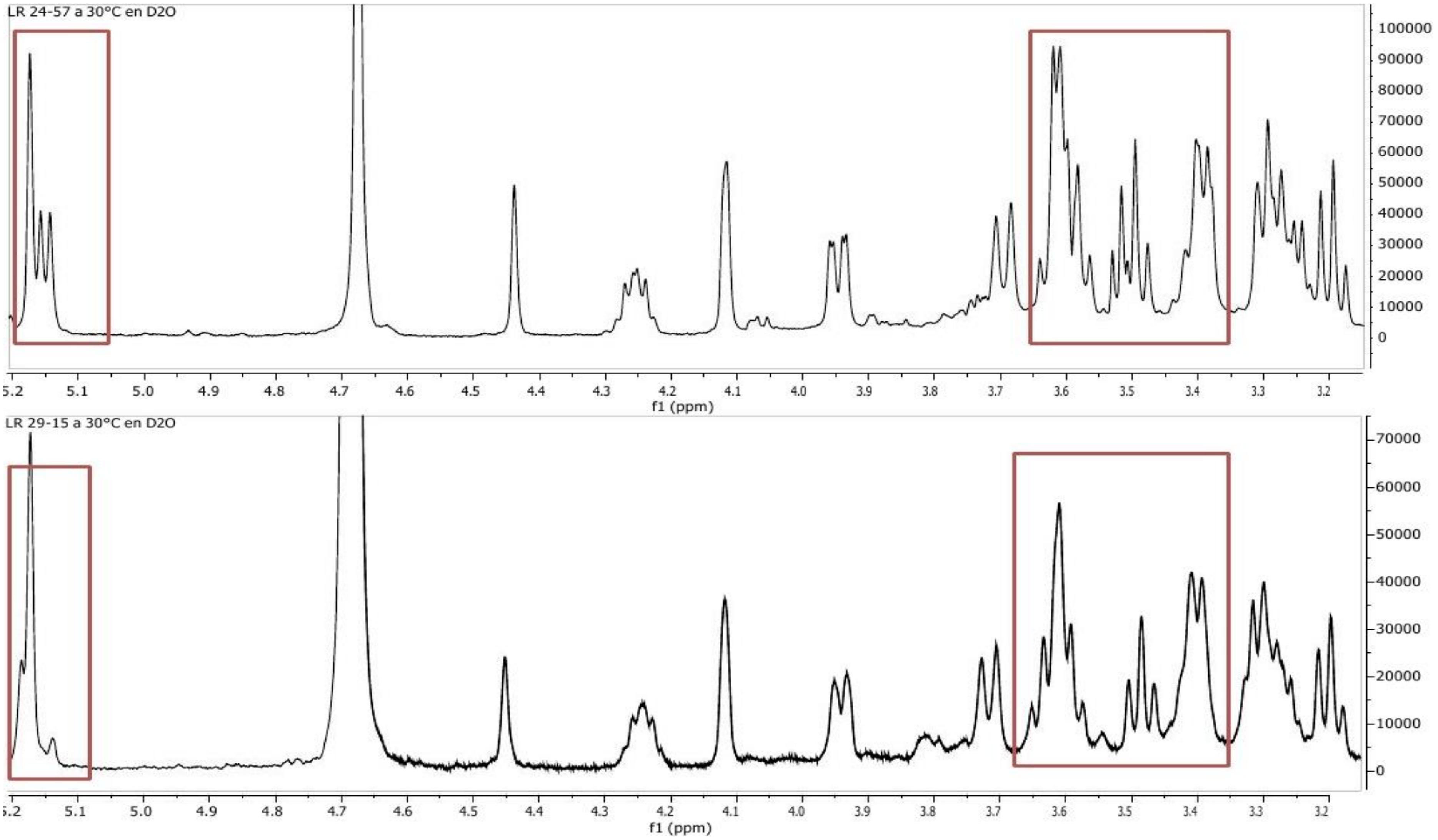

Figure S59. Comparison of ^{1}H NMR spectrum of compound LR 24 – 57 and LR 29- 15 in deuterium oxide at 30°C.

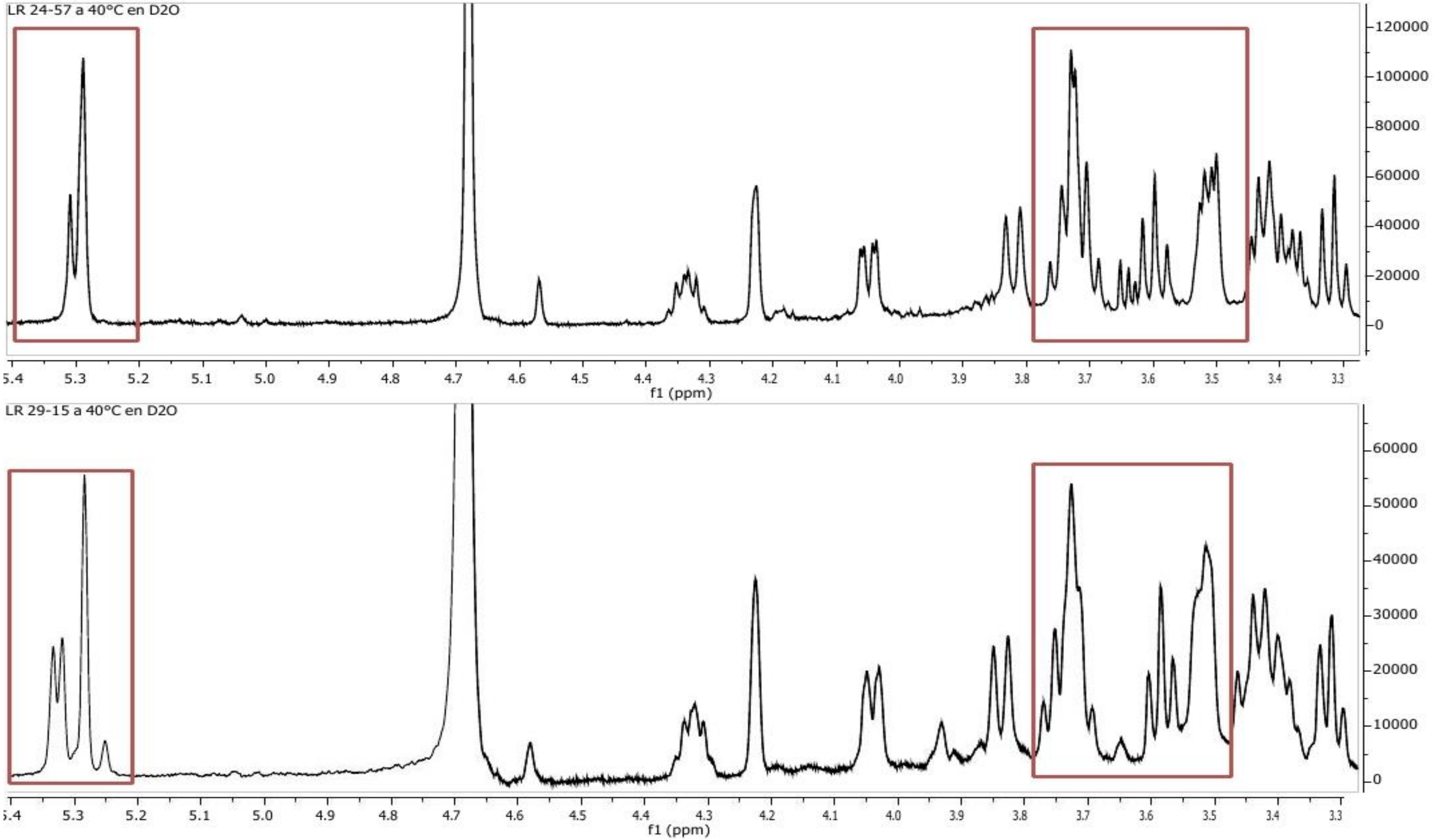

Figure S60. Comparison of ^{1}H NMR spectrum of compound LR 24 – 57 and LR 29- 15 in deuterium oxide at 20°C.
